# A Divergent Asymmetric Total Synthesis of Coprophilin and Four Trichodermic Acids via a [1,5]-Hydride Shift–Aldol Cascade

**DOI:** 10.1021/jacs.5c17359

**Published:** 2025-12-18

**Authors:** Edward Smith, Timothy C. Jenkins, Charles S. Yeung, Timothy J. Donohoe

**Affiliations:** a Department of Chemistry, Chemistry Research Laboratory, 6396 University of Oxford, Mansfield Road, Oxford OX1 3TA, U.K.; b Merck & Co., Inc., 33 Avenue Louis Pasteur, Boston, Massachusetts 02115, United States

## Abstract

The asymmetric syntheses of coprophilin and four members of the trichodermic acid family of natural products are disclosed. Our work employs a number of key transformations, including an aluminum-promoted [1,5]-hydride shift–aldol cascade reaction, an *exo*-selective Diels–Alder cycloaddition, and a late-stage Fleming–Tamao oxidation. These key steps efficiently construct the bicyclic core of the natural products, which can then be readily functionalized in a divergent manner, allowing the synthesis of a wide range of natural product targets.

## Graphical abstract

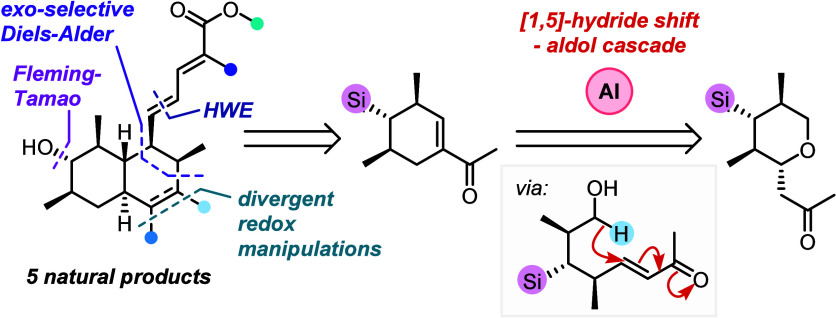

## Introduction

Natural products have long served as inspiration for the discovery of new medicines^1^. For instance, *trans*-decalin-derived natural products bearing a C-2 hydroxyl group, a motif found in steroidal structures as well as **1–6**, are of considerable medicinal interest (Figure 1A). (+)-Coprophilin (**1**), first isolated by MSD in 1998 from a fungus designated MF 5773, was shown to inhibit the growth of *E. tenella*, a harmful protozoan associated with coccidosis in poultry^2^. (+)-Trichodermic acid (**2**) and its synthetic derivative AMF-26 have been investigated as inhibitors of the Golgi system, with potential applications in cancer treatment.^3,4^ Since the isolation of (+)-trichodermic acid (**2**) in 2005, eight new derivatives have been isolated from the endophytic fungus *Trichoderma spirale*: A and B in 2012, C and D in 2021 and E, F, G and H in 2023.^5–7^ Their structures have been elucidated using NMR analysis, and their absolute configurations have been proposed by computational methods and by analogy to the known compound **2**, though not conclusively proven by chemical methods. These compounds have subsequently been investigated as antitumor agents as well as antifungals. Each member of this family possesses a unique oxidation pattern, primarily on the right-hand ring (as drawn). A synthetic route which offers access to a number of natural products from one late-stage intermediate would be very valuable and would contribute to their continued study.

**Figure 1:**
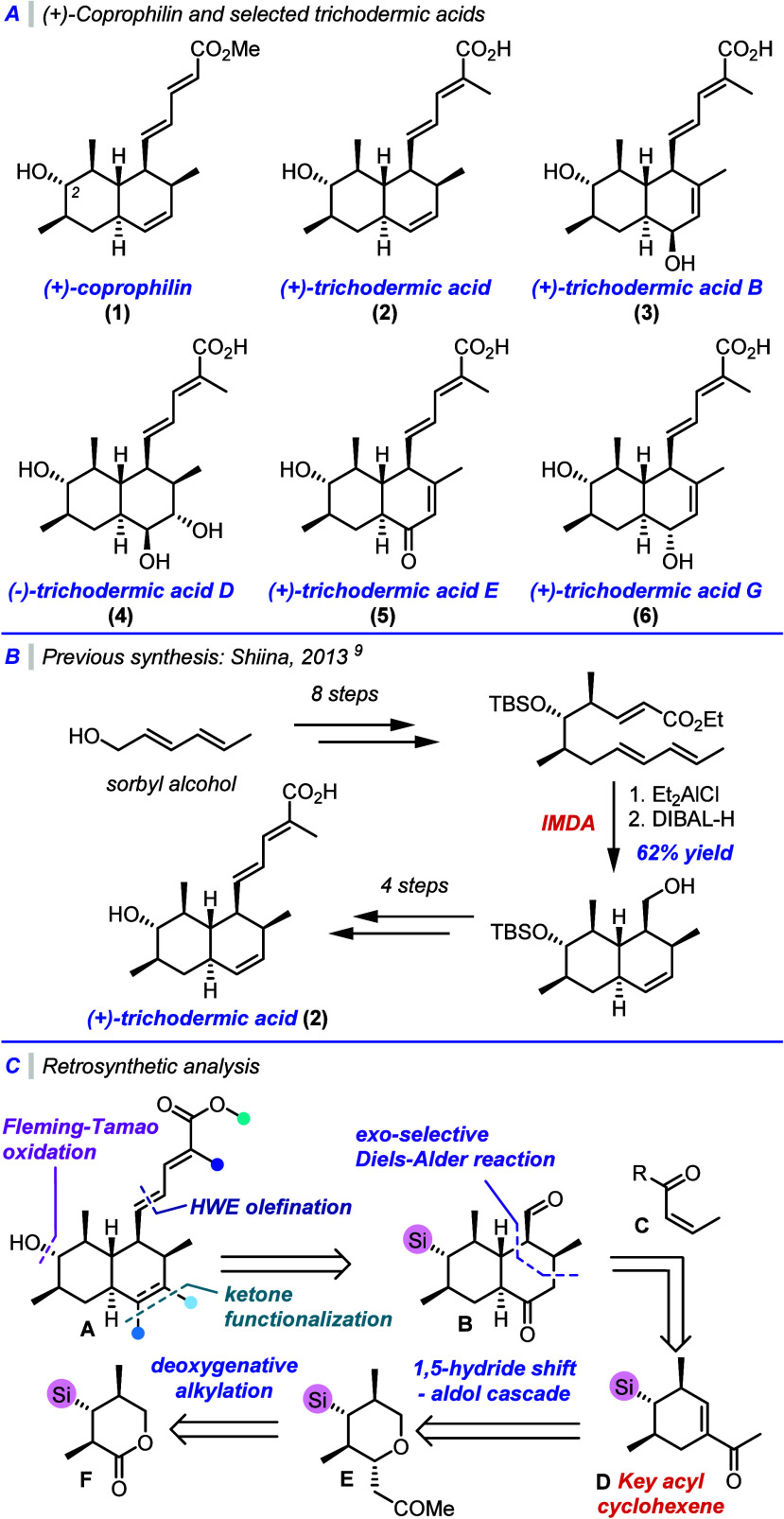
(A) (+)-Coprophilin and selected trichodermic acid natural products. (B) Previous synthesis of (+)-trichodermic acid. (C) Retrosynthesis.

The molecular structures of these compounds make them interesting synthetic targets for a number of reasons. First, their high stereochemical density, with up to nine contiguous stereogenic centers, poses a significant challenge in their stereoselective synthesis. Additionally, the high degree of unsaturation in many of the compounds requires careful synthetic strategies and the use of selective reagents to avoid unwanted side reactions. The diverse range of oxidation patterns on the right-hand rings of these compounds also presents an opportunity for a divergent synthesis of this class of natural product. As will be detailed later, we considered these targets to be ideal candidates on which to expand, and test the limits of, our recently developed synthesis of acyl cyclohexenes via a [1,5]-hydride shift – aldol cascade reaction^8^.

Previously, Shiina and co-workers reported a total synthesis of trichodermic acid, employing a biomimetic intramolecular Diels–Alder strategy (Figure 1B)^9^. The requisite linear precursor was prepared in 8 steps from sorbyl alcohol, which cyclized upon treatment with diethylaluminum chloride. The bicyclic intermediate was then converted into (+)-trichodermic acid (**2**) in 5 further steps. In total, this sequence gave (+)-trichodermic acid (**2**) in 14 linear steps, which was, along with some analogues, investigated for anticancer activity.

These analogues were made by functionalization of the unsaturated side chain, but no derivatization on the central octahydronaphthalene core was investigated, presumably owing to the synthetic challenge of doing so. This route therefore offers limited scope for core diversification and access to other members of the broader trichodermic acid family. More recently, Shiina and co-workers used a similar route to synthesize (+)-coprophilin (**1**), confirming its structure and absolute stereochemistry.^10^

## Results and Discussion

Our retrosynthetic strategy revolves around an efficient synthesis of the *trans*-decalin core of the natural product family (**A**, Figure 1C) enabled through our recently reported [1,5]-hydride shift chemistry.^8^ A single common intermediate **B** could then be transformed into a variety of natural products by employing different synthetic strategies.

In all cases, a HWE olefination would be employed to install the unsaturated side chain onto **B**, and we chose a common silyl precursor to the alcohol functionality in **A** in order to avoid lengthy protection/deprotection sequences. Finally, a late-stage Fleming-Tamao oxidation would be used to unmask the alcohol on the backbone. In this scenario, the diverse oxidation pattern on the second ring of the natural products could be accessed by differential ketone functionalization reactions in a divergent approach.

The *trans*-decalin core **B** would be constructed using two key cyclization steps. First, we envisaged an *exo*-selective Diels–Alder reaction employing a *cis*-configured dienophile **C** to construct the right-hand ring. The required diene could then be prepared from the corresponding methyl ketone **D**, prepared from **E** using an aluminum promoted [1,5]-hydride shift – aldol cascade reaction, thereby efficiently constructing the left-hand ring.^8,11^ In this case, it was unknown as to whether the stereochemical integrity of substrate **E** would be retained through this key step, as there are substantial opportunities for epimerization during this cascade.

We hoped that tetrahydropyran **E** could be accessed from lactone **F**, *via* a deoxygenative alkylation approach. In doing so, we would be able to efficiently construct the desired cyclohexene **D** from lactone **F** in just two steps.

This novel route is uniquely enabled by our method; the ability to rapidly access versatile acyl cyclohexenes from lactones (of which there are many known examples in the literature) with high levels stereo- and regiocontrol offers a new method for six-membered carbocycle synthesis, *via* a [1,5]-hydride shift.

Our work began on the synthesis of substituted tetrahydropyran **11** (Scheme 1A). Following a modified literature procedure, (*S*)-Roche ester **7** was protected and reduced in one pot, and subsequently treated with the lithium enolate of ethyl acetate and then heated with TsOH overnight to afford lactone **8**.^12^ Stereoselective installation of a dimethylphenylsilyl group *via* cuprate addition, and *in situ* alkylation using methyl iodide then afforded lactone **9** as a single *trans* diastereomer.^13^

**Scheme 1:**
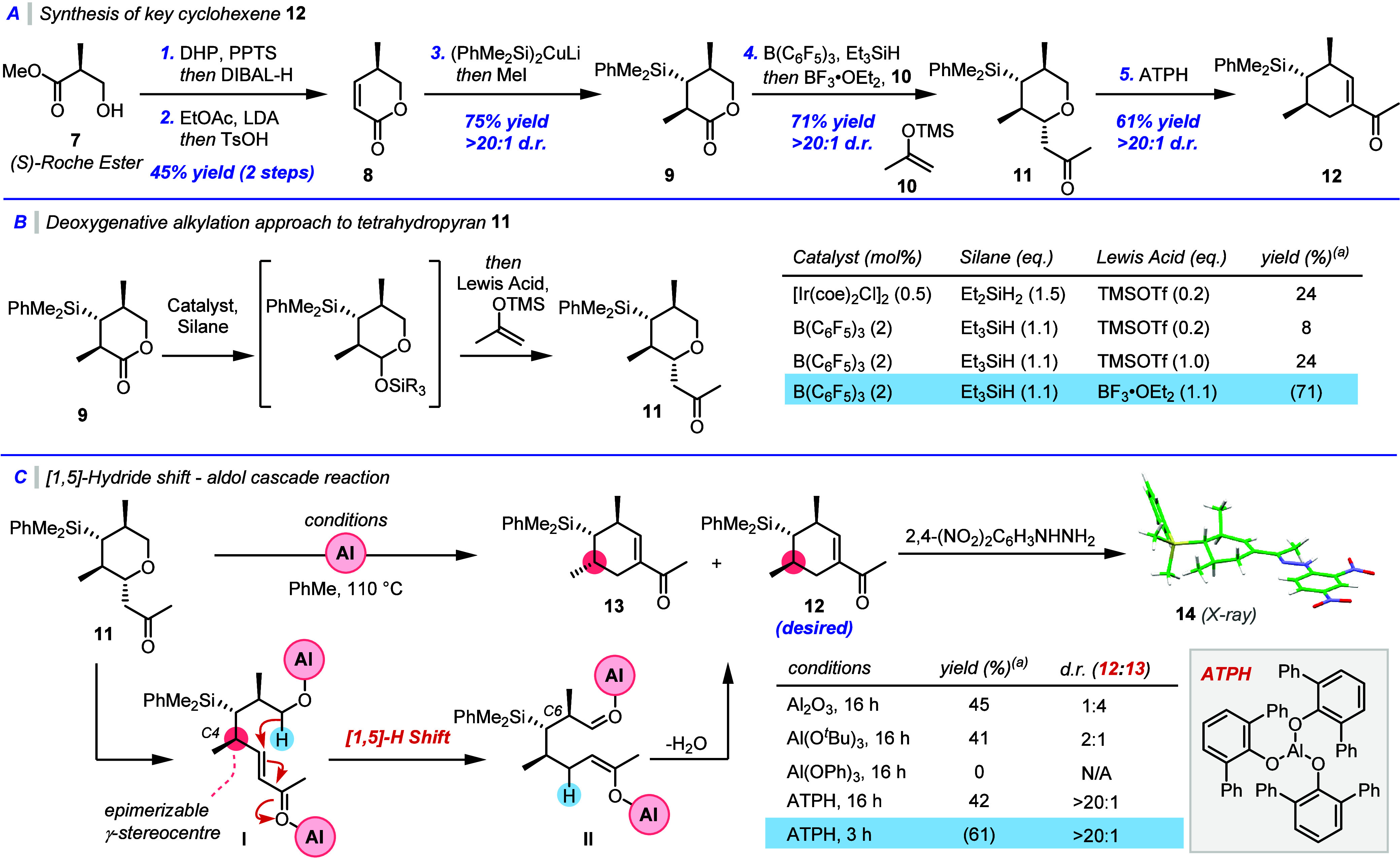
(A) Synthetic Route to Cyclohexene **12**; (B) Optimization of Deoxygenative Alkylation Reaction; (C) Optimization of [1,5]-Hydride Shift–Aldol Cascade Reaction

a Yield refers to quantitative ^1^H NMR yield using 1,1,2,2-tetrachloroethane as an internal standard. Isolated yield in parentheses.

In order to access the desired tetrahydropyran **11**, we employed a new deoxygenative alkylation protocol (Scheme 1B). We envisaged that this could proceed *via* initial hydrosilylation of lactone **9**, and subsequent Mukaiyama aldol reaction of the silyl acetal thus formed with nucleophile **10**. Initially, we employed the iridium-catalyzed hydrosilylation reported by Brookhart and co-workers and advanced in reductive alkylation reactions by Dixon and co-workers.^14,15^ Unfortunately, these conditions gave poor yields, primarily owing to overreduction from the excess hydride present. We next turned to the hydrosilylation protocol developed by Soos and co-workers.^16^ This usually requires bespoke borane catalysts, but we were pleased to find that in this case hydrosilylation occurred smoothly using commercially available tris­(pentafluorophenyl)­borane. Disappointingly, TMSOTf – promoted Mukaiyama aldol reaction then gave poor yields of the desired product, but when BF_3_ was employed as a Lewis acid we were able to isolate **11** in a 71% yield from **9** as a single diastereomer. The relative stereochemistry of **11** was confirmed by NOESY analysis; here, the incorporation of the silyl enol ether nucleophile as an equatorial substituent is interesting. Addition of nucleophiles to half-chair six-membered oxocarbenium ions is well-documented in the literature, with axial attack typically being preferred.^17^ In this case, the presence of the large silyl group may well distort the conformation of the lactone and any oxocarbenium ion derived from it, thus complicating analysis.^18^ Of course, it is also possible that the all-equatorial **11** is the thermodynamic product, arising from reversible ring-opening of the tetrahydropyran.^19^

Next, we moved to investigate the key [1,5]-hydride shift – aldol cascade reaction^8^. Our previously reported conditions, employing alumina or aluminum *tert*-butoxide as Lewis acids, gave an interesting result (Scheme 1C). While the desired cyclohexene product **12** was formed in both cases, substantial epimerization was observed at C4 to give **13**; here it arises from γ-deprotonation/reprotonation of the ring-opened enone form **I** of the THP starting material. The high level of epimerization observed when using alumina as a promoter suggests that the undesired epimer undergoes the [1,5]-hydride shift more rapidly than the desired epimer. The lack of any epimerization at C6 is also interesting, and we presume that this is because of the rapid nature of the subsequent intramolecular aldol reaction of the otherwise vulnerable aldehyde **II**.

We hypothesized that increasing the rate of the hydride shift reaction would reduce the extent of any C4 epimerization by rapidly removing the ring-open form from the mixture before it had time to lose a proton. Investigating phenol-based ligands for aluminum, we found that aluminum phenoxide displayed no reactivity. However, using a bulky phenoxide ligand on the aluminum, forming known complex ATPH, gave rapid reactivity to form the product *and* completely suppressed epimerization under the reaction conditions, forming **12** as a single diastereomer in which all stereogenic centers had been retained.^20,21^ We suggest that the stark difference in reactivity is owed to the speciation of the Lewis acids involved. Aluminum *tert*-butoxide and aluminum phenoxide have been shown to be dimeric or oligomeric in solution, while ATPH is reported to be monomeric, and so is a much more reactive Lewis Acid under these conditions.^20–22^ As a result, we suggest that ATPH is particularly effective in promoting this reaction, and thus the desired product was obtained as a single diastereomer before any epimerization could occur. The failure of other phenol-based ligands to suppress epimerization (see Supporting Information for details) suggests that the relatively low p*K*_a_ of phenoxide is not a key factor in reducing epimerization. Pleasingly the relative stereochemistry of cyclohexene **12** was confirmed by single crystal X-ray analysis of the corresponding 2,4-dinitrophenylhydrazone **14**.^23^

With the key cyclohexene in hand, we began our investigation into construction of the second ring using an *exo*-selective Diels–Alder strategy (Scheme 2). Formation of TBS enol ether **15** followed by treatment with dienophile (*R*)-**16** and an alkylaluminum Lewis acid resulted in a Diels–Alder annulation which exhibited perfect facial- and *exo*-selectivity. However, this reaction was impeded by an unwanted *Z* to *E* isomerization of the dienophile *in situ*, leading to isolation of *cis* and *trans* epimers of adduct **17**.^24^

**Scheme 2:**
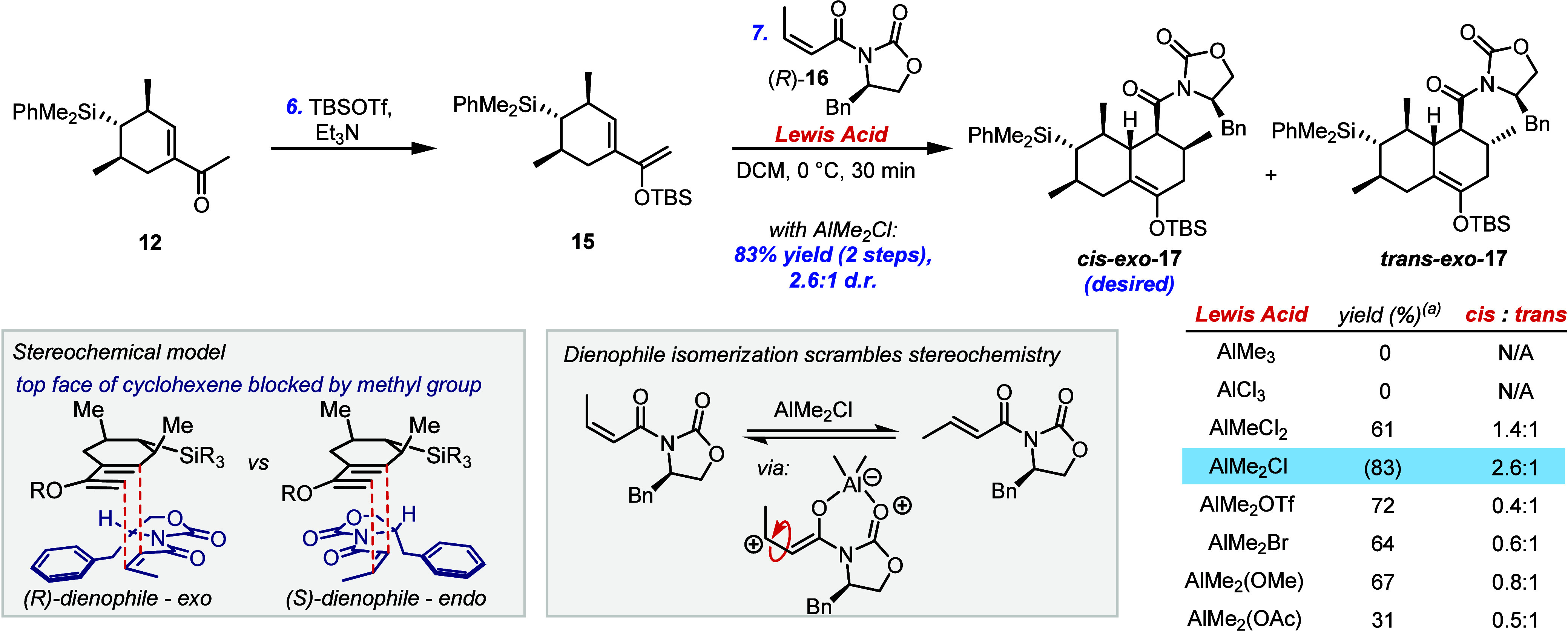
*Exo*-Selective Diels–Alder Reaction

a Yield refers to quantitative ^1^H NMR yield using 1,1,2,2-tetrachloroethane as an internal standard. Isolated yield in parentheses.

The structures of these Diels–Alder adducts were assigned by comprehensive NOESY analysis; additionally, the structure of *trans-exo-* **17** was confirmed by comparison of its NMR data to that of an analogue bearing a different oxazolidinone auxiliary, whose structure was confirmed by X-ray crystallography (see Supporting Information for details). The structure of *cis-exo-* **17** was further confirmed by completion of the total syntheses of (+)-coprophilin (**1**) and (+)-trichodermic acid (**2**), whose structures have previously been confirmed through their total syntheses.

In all cases where the cycloaddition took place, any excess unreacted dienophile present had completely isomerized to the *E* isomer. Screening a range of aluminum Lewis acids failed to completely suppress this isomerization, although by using dimethylaluminum chloride we were able to obtain the desired *cis* diastereomer in a good yield.

The failure of dimethylaluminum triflate to suppress the dienophile isomerization suggests that it does not take place via a nucleophilic or base-promoted pathway enabled by the free halide counterion, but by bond rotation, following substantial lowering of the double-bond character of the C=C bond in the dienophile-Lewis acid complex.

This undesired reactivity has previously been observed by Evans and co-workers, who concluded that *cis* substituted dienophiles were not suitable for these types of Diels–Alder reactions.^24a^ The success of our reaction herein is presumably owed to the high rate of cycloaddition relative to the undesired isomerization, which appears to be highly dependent on the Lewis acid used.

It was necessary to use a chiral dienophile to achieve good *exo* selectivity; using the opposite dienophile enantiomer gave a mixture of *cis-* and *trans-endo* adducts (see Supporting Information for details), which can be rationalized when Evans’ stereochemical model is applied to this system, and assuming that the top face of the diene is sterically inaccessible.^24a,25^

Fortunately, the two diastereomeric products *cis-exo-* **17** and *trans-exo-* **17** were separable by column chromatography, allowing the desired *cis* isomer to be cleanly isolated in 60% yield, and this material was used going forward (Scheme 3). Attempts at reductive cleavage of the oxazolidinone were made, but unfortunately the sterically hindered carbonyl group was unreactive.

**Scheme 3:**
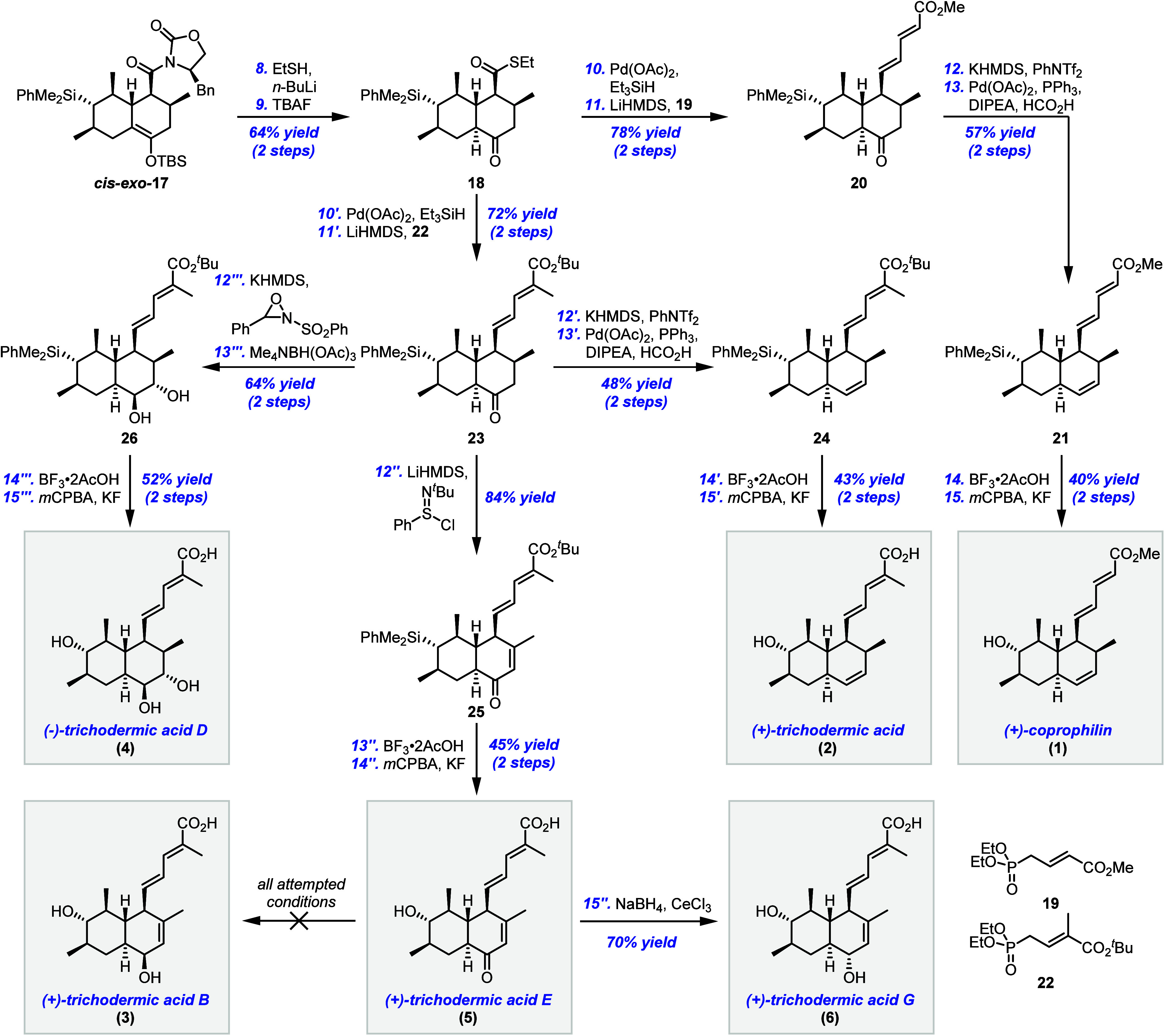
Total Synthesis of (+)-Coprophilin and Four Trichodermic Acids

Therefore, we opted for cleavage of the auxiliary with ethanethiol. Treatment of *cis-exo*-**17** with ethanethiol in the presence of *n*-BuLi cleanly removed the auxiliary. Subsequent cleavage of the silyl enol ether using TBAF in THF at low temperature afforded the desired *trans* decalone **18** as a single diastereomer, presumably via kinetic (axial) protonation of the enolate formed *in situ*.

Our first objective was the total synthesis of (+)-coprophilin (**1**), and the unsaturated side chain was attached using a Fukuyama reduction - HWE olefination sequence. Here, it was found that the use of catalytic palladium­(II) acetate instead of palladium on carbon was beneficial to ensure the reproducibility of this key reduction. The subsequent HWE olefination using phosphonate **19** pleasingly gave exclusively the *E* isomer of **20**.

Next, ketone **20** was deoxygenated through formation of the corresponding vinyl triflate, followed by palladium mediated reduction to furnish **21**. Finally, a two-step Fleming-Tamao oxidation,^26^ via the corresponding fluorosilane, cleanly afforded (+)-coprophilin (**1**), whose spectroscopic data and specific rotation matched that in the literature.^2,10^ We were pleased to find that this oxidative step proceeded with retention of configuration, and also that none of the three alkenes in the molecule were oxidized.

Our focus then turned to the synthesis of (+)-trichodermic acid (**2**), the parent compound of its family. This compound was prepared via a similar route to (+)-coprophilin (**1**), but using methyl substituted allylic phosphonate **22** in the HWE olefination, along with the aforementioned ketone deoxygenation procedure to afford **24**. Pleasingly, the strongly acidic conditions of the first step of the Fleming-Tamao oxidation of **24** also cleaved the *tert*-butyl ester to the corresponding carboxylic acid, and (+)-trichodermic acid (**2**) was thus obtained; again the spectroscopic data and specific rotation matched that reported previously^9^.

Our third target was (+)-trichodermic acid E (**5**), which retains the ketone moiety installed in our route. So, desaturation of ketone **23** using Mukaiyama’s protocol afforded enone **25**,^27^ after which a Fleming-Tamao oxidation then gave (+)-trichodermic acid E (**5**), with our data matching the literature and thus confirming the structure and absolute stereochemistry of this compound through this first reported synthesis.

Next, Luche reduction of the ketone moiety in trichodermic acid E (**5**) gave (+)-trichodermic acid G (**6**), with the relative stereochemistry confirmed by NOESY analysis, as a single diastereomer. Interestingly, its epimer, (+)-trichodermic acid B (**3**), was not observed in this reduction and despite numerous attempts, we were unable to obtain (+)-trichodermic acid B (**3**) by the reduction of (+)-trichodermic acid E (**5**). A number of different reducing agents were screened (see Supporting Information for details), but whenever reduction was observed, only (+)-trichodermic acid G (**6**) was obtained.

Finally, we resolved to synthesize (−)-trichodermic acid D (**4**), which possesses 9 contiguous stereocenters and would require us to selectively install an *anti*-1,2-diol moiety in place of the ketone. This was achieved through α-hydroxylation of ketone **23**, followed by directed 1,2-reduction using tetramethylammonium triacetoxyborohydride. We were pleased to find that this sequence afforded diol **26** as a single diastereomer, with the stereochemistry confirmed by NOESY analysis. Pleasingly, diol **26** successfully underwent a Fleming-Tamao oxidation to give (−)-trichodermic acid D (**4**), again whose data matched that reported in the literature^6^.

## Conclusions

In conclusion, we have completed total syntheses of (+)-coprophilin (**1**) and four members of the trichodermic acid family of natural products, successfully establishing a new synthetic approach to this class of natural product. The structures and absolute configurations of trichodermic acid D, E, and G were confirmed through completion of the first total syntheses of these compounds, showing that this family of natural products all possess the same absolute configuration in the common scaffold. The synthesis was achieved by employing a key [1,5]-hydride shift – aldol cascade reaction followed by an *exo*-selective Diels–Alder reaction to sequentially construct the *trans*-decalin core. Subsequent ketone functionalization enabled the synthesis of a diverse range of trichodermic acid derivatives, which were synthetically inaccessible by previously reported methods.

## Supplementary Material



## Notes

Experimental details, spectroscopic data, and copies of ^1^H/^13^C NMR spectra for all compounds (PDF)
