# Supplementary material for: A Divergent Asymmetric Total Synthesis of Coprophilin and Four Trichodermic Acids via a [1,5]-Hydride Shift–Aldol Cascade
Source: J Am Chem Soc. 2025 Dec 18;148(1):1252–8. doi: 10.1021/jacs.5c17359 (PMC12814181; doi:10.1021/jacs.5c17359)
Supplement: Supplementary file 1 [file ja5c17359_si_001.pdf]

A Divergent Asymmetric Total Synthesis of Coprophilin and Four Trichodermic Acids *via* a [1,5]-hydride shift – aldol cascade: Supporting information.

Edward Smith,[a] Timothy C. Jenkins,[a¥] Charles S. Yeung,[b] and Timothy J. Donohoe[a]*

[a] Department of Chemistry, University of Oxford, Chemistry Research Laboratory, Mansfield Road, Oxford, OX1 3TA, UK. [b] Merck & Co., Inc, 33, Avenue Louis Pasteur, Boston, MA 02115, USA. [¥]Author to whom correspondence regarding the X-ray crystal structures should be addressed.

1. **Additional reaction optimization** **S2**

2. **General Experimental** **S5**

3. **Preparation and characterisation of compounds** **S6**

4. **NMR tables of natural products** **S36**

5. **NMR spectra of novel compounds** **S41**

6. **X-Ray Crystallography Data** **S69**

7. **References** **S73**

1. Additional reaction optimization

1.i Aluminium phenoxides screened in [1,5]-hydride shift – aldol cascade reaction.

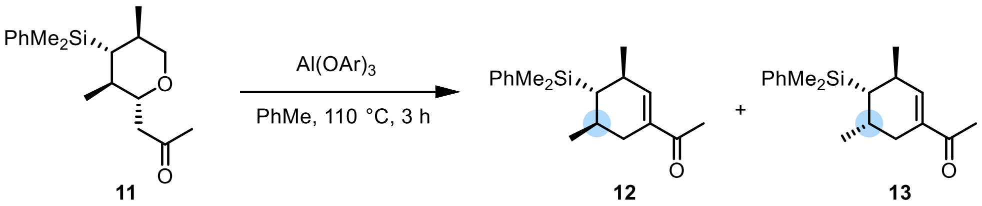

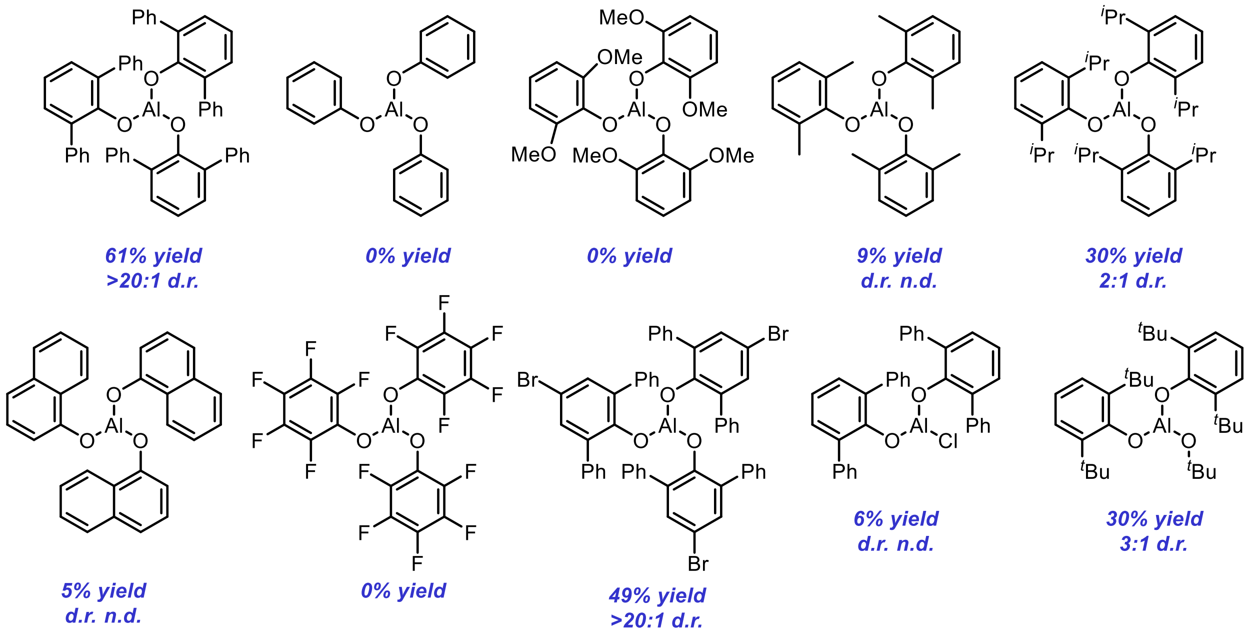

1.ii Diels-Alder reaction with different chiral auxiliaries.

(R)-evans:

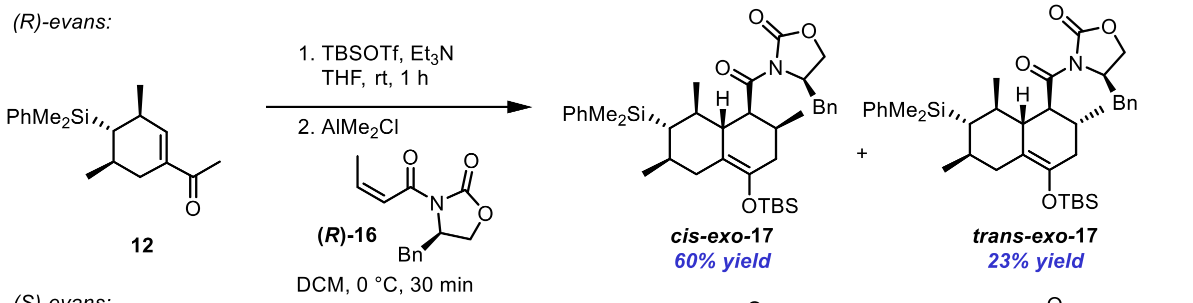

(S)-evans:

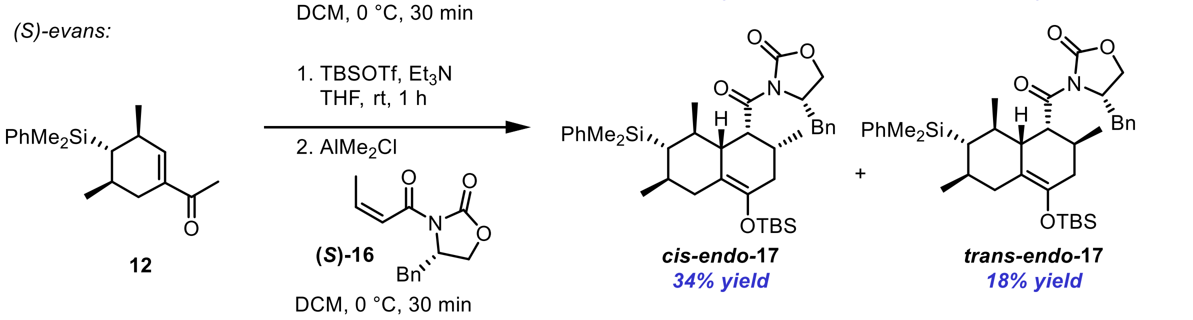

(R)-superquat:

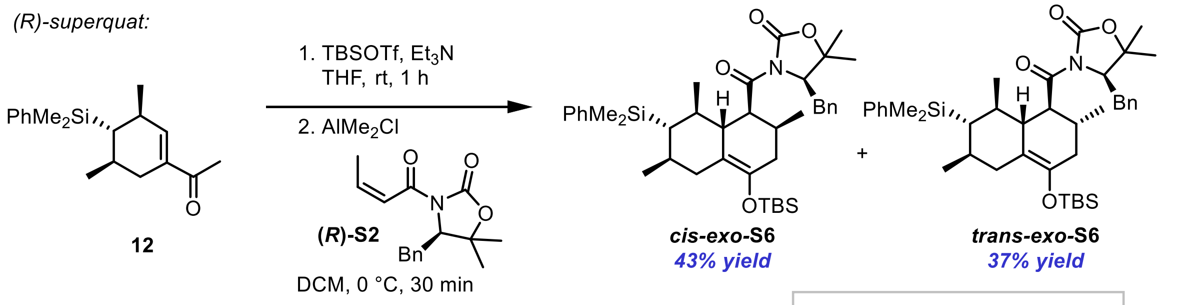

x-ray structure of trans-exo-S6

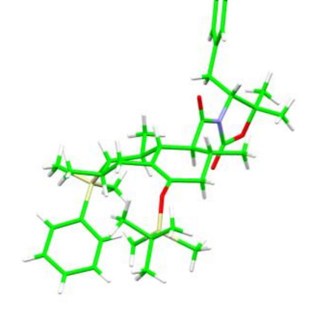

1.iii Reduction of Trichodermic Acid E.

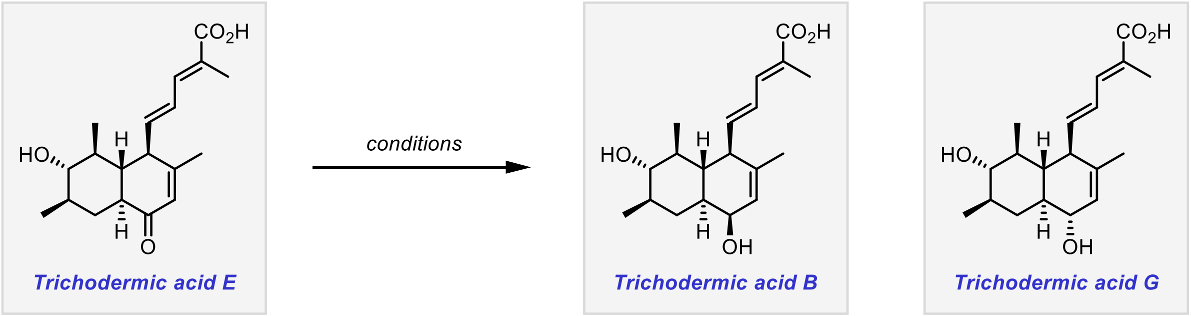

conditions	*E (%)*	*B (%)*	*G (%)*
$NaBH_4$, $CeCl_3$, MeOH, 0 °C, 30 min	0	0	70
$LiAlH(O^tBu)_3$, THF, 0 °C, 1 h	16	0	37
$KBH(O^iPr)_3$, THF, 0 °C, 1 h	6	0	65
$Al(O^iPr)_3$, ^{i}PrOH, 80 °C, 4 h	0	0	49
RuCl(p-cymene)[(S,S)-Ts-DPEN], *i*PrOH, rt, 16 h	75	0	0
RuCl(p-cymene)[(S,S)-Ts-DPEN], HCO_2H, Et_3N, 60 °C, 4 h	30	0	0
(-)-ipc_2BCl, THF, 0 °C, 1 h	75	0	0

2. General Experimental

Unless otherwise stated, all commercial chemicals and solvents were used without further purification. All air/moisture sensitive procedures were carried out in flame-dried glassware under an argon/nitrogen atmosphere. Reactions at 0 °C were performed using an ice-water bath; reactions at -78 °C were performed using a dry ice-acetone bath. Other temperatures below 0 °C were obtained using a Julabo FT902 immersion cooler; temperatures above 25 °C were achieved using a hot plate and oil bath. FCC used Merck Geduran silica gel (40-63 µm or 15-40 µm particle size). The removal of solvents *in vacuo* was achieved using a Büchi rotary evaporator with an oil pump (0.1 mmHg) or diaphragm pump (15 mmHg) at bath temperatures up to 60 °C.

^{1}H, ^{13}C and ^{31}P NMR experiments were carried out at room temperature, using Bruker NMR spectrometers (400, 500, or 600 MHz) in the deuterated solvent stated, and referenced to residual solvent peaks. Chemical shifts are quoted in parts per million (ppm) and to the nearest 0.01 ppm for ^{1}H and ^{31}P spectra, and to the nearest 0.1 ppm for ^{13}C spectra. Coupling constants (*J*) are quoted to the nearest 0.5 Hz. Resonances are described as s (singlet), d (doublet), t (triplet), q (quartet), (pentet) and m (multiplet). Assignments were made with the assistance of COSY, HSQC and HMBC NMR spectra. Quantitative NMR experiments were run on a Bruker AVIII400 spectrometer using a 25 second relaxation time between scans.

Infrared spectra were recorded with a Tensor 27 FT-IR with internal calibration in the range 600-4000 cm-1 and absorption maxima (vmax) are quoted in wavenumbers (cm-1). Flow injection analysis was performed on an ACQUITY I-Class PLUS UPLC System (Waters, Milford, MA, USA) coupled to an ACQUITY RDa mass spectrometer (Waters, Milford, MA, USA) equipped with an ESI probe, in positive ion mode. The flow rate was set to 0.300 mL/min using a 50% methanol(aq) + 0.1% formic acid eluent. Scan parameters were set as follows: analyser mode, full scan; scan range, 50 2000 m/z; scan rate, 2 Hz; cone voltage, 30 V; capillary voltage, 1.5 kV; desolvation temperature, 550 °C; and intelligent data capture, on. Melting points were determined using a Griffin melting point apparatus and are uncorrected.

Single crystal X-ray data collection and structure determination were performed by Timothy C. Jenkins in the Chemistry Research Laboratory, University of Oxford. Crystals were mounted on MiTeGen loops using perfluoropolyether oil and rapidly transferred to a goniometer head on a diffractometer fitted with an Oxford CryoSystems CryoStream open-flow nitrogen cooling device.[1] Data collections were carried out at 150 K using an (Rigaku) Oxford Diffraction Supernova A diffractometer using mirror-monochromated Cu Kα radiation (λ = 1.54184 Å) and data were processed using CryAlisPro. The structure was solved using charge-flipping algorithm (SUPERFLIP) and refined by full-matrix least squares using CRYSTALS.[2-4]

3. Preparation and characterisation of compounds

3.i Procedures and intermediates for the synthesis of Coprophilin and four Trichodermic acids.

(*R*)-5-Methyl-5,6-dihydro-2H-pyran-2-one **8**

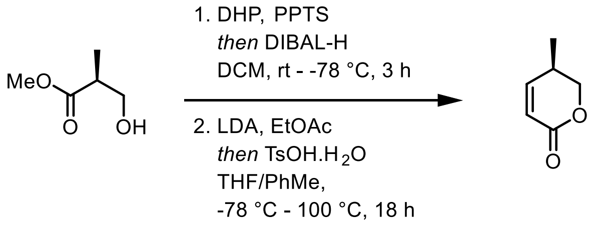

A 1 L RBF was charged with methyl (*S*)-3-hydroxy-2-methylpropanoate (10.0 g, 84.7 mmol, 1.00 equiv.) in dry DCM (300 mL). 3,4-Dihydro-2H-pyran (7.7 mL, 85 mmol, 1.0 equiv.) was then added followed by pyridinium p-toluenesulfonate (1.06 g, 4.24 mmol, 0.0500 equiv.) and the mixture was stirred at rt for 2 h, before being cooled to -78 °C. DIBAL-H (1.0 M in hexanes, 93 mL, 93 mmol, 1.1 equiv.) was added dropwise and the mixture was stirred at -78 °C for 15 min, and then quenched by addition of saturated aqueous Rochelle's salt (300 mL). The mixture was warmed to rt and stirred vigorously for 1 h. The layers were separated and the aqueous phase was extracted with DCM (2 x 300 mL). The combined organic phases were dried over Na_2SO_4 and concentrated *in vacuo* to afford the crude aldehyde.

A 1 L RBF was charged with *N,N*-diisopropylamine (14 mL, 100 mmol, 1.2 equiv.) in dry THF (300 mL) and cooled to -78 °C. *N*-BuLi (2.5 M in hexanes , 40 mL, 100 mmol, 1.2 equiv.) was then added dropwise and the reaction was stirred at rt for 30 min. The mixture was then cooled to -78 °C and ethyl acetate (10 mL, 100 mmol, 1.2 equiv.) was added dropwise and stirred for 1 h. The crude aldehyde was then added and the mixture stirred at -78 °C for 15 min. A suspension of $TsOH.H_2O$ (58.0. 305 mmol, 3.60 equiv.) in PhMe (300 mL) was then added followed by Na_2SO_4 (50 g) and the mixture was heated at 100 °C for 16 h. Upon cooling, the mixture was filtered through a plug of silica, washing with DCM (500 mL), and concentrated *in vacuo*. Purification by FCC (50-100% DCM/Pentane) afforded the title compound as a colourless oil (4.30 g, 38.4 mmol, 45% yield).

^{1}H NMR (400 MHz, $CDCl_3$) **δ** 6.78 (dd, *J* = 9.8, 3.5 Hz, 1H), 5.95 (dd, *J* = 9.5, 2.0 Hz, 1H), 4.39 (dd, *J* = 11.0, 5.0 Hz, 1H), 4.04 (dd, *J* = 11.0, 8.5 Hz, 1H), 2.75 – 2.60 (m, 1H), 1.13 (d, *J* = 7.0 Hz, 3H).

^{13}C NMR (101 MHz, $CDCl_3$) **δ** 163.8, 151.7, 120.4, 72.2, 29.0, 15.6.

$[\alpha]_D^{20}$ = -39.6 (c = 1.0, $CHCl_3$). **Lit $[\alpha]_D^{23}$** = -42.0 (c = 1.0, $CHCl_3$).[5]

HRMS (ESI+): Found [M+Na]+ = 135.0410; $C_6H_8O_2Na$ requires 135.0417, Δ -4.85 ppm.

IR (film) vmax/cm^{-1}: 2942, 1732, 1469, 1264, 1229, 828.

The spectroscopic data matched that previously reported in the literature.[5]

(3*R*,4*R*,5*S*)-4-(Dimethyl(phenyl)silyl)-3,5-dimethyltetrahydro-2H-pyran-2-one **9**

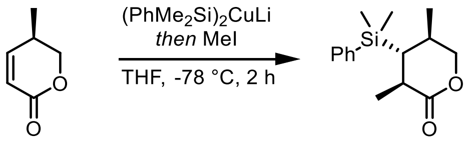

A 500 mL RBF was charged with Li wire (2.64 g, 381 mmol, 10.0 equiv.) in dry THF (100 mL) at 0 °C under nitrogen. Chloro(dimethyl)phenylsilane (15 mL, 91 mmol, 2.4 equiv.) was added dropwise and the mixture was allowed to warm to rt and stirred overnight, forming a dark red solution. After this time, this solution was transferred *via* syringe to a 500 mL RBF containing copper (I) iodide (8.71 g, 45.7 mmol, 1.20 equiv.) at -78 °C, and the resulting mixture was stirred for 1 h. Unsaturated lactone **8** (4.27 g, 38.1 mmol, 1.00 equiv.) was then added dropwise, and the mixture was stirred at -78 °C for 2 h. MeI (12 mL, 190 mmol, 5.0 equiv.) was then added and the reaction was stirred for 2 h at -78 °C before being warmed to rt and stirred for 1 h. The reaction was quenched by addition of saturated aqueous ammonium chloride solution (100 mL), and diluted with concentrated aqueous ammonia solution (300 mL) and EtOAc (300 mL). The layers were separated and the aqueous phase was extracted with EtOAc (2 x 300 mL). The combined organic layers were dried over Na_2SO_4 and concentrated in vacuo. Purification by FCC (20% Et_2O/Pentane) afforded the title compound as a colourless oil (7.53 g, 28.7 mmol, 75% yield.).

^{1}H NMR (400 MHz, $CDCl_3$) **δ** 7.55 – 7.46 (m, 2H), 7.44 – 7.31 (m, 3H), 4.01 (dd, *J* = 11.5, 3.5 Hz, 1H), 3.94 (dd, *J* = 11.5, 3.5 Hz, 1H), 2.53 – 2.40 (m, 1H), 2.11 – 1.98 (m, 1H), 1.14 (d, *J* = 7.0 Hz, 3H), 0.94 (d, *J* = 7.0 Hz, 3H), 0.69 (dd, *J* = 10.5, 5.5 Hz, 1H), 0.44 – 0.36 (m, 6H).

^{13}C NMR (101 MHz, $CDCl_3$) **δ** 176.8, 137.0, 133.9, 129.7, 128.2, 71.6, 34.7, 33.3, 30.3, 21.1, 17.8, -3.5, -3.7.

$[\alpha]_D^{20}$ = +26.3 (c = 1.0, $CHCl_3$).

HRMS (ESI+): Found [M+H]+ = 263.1453; $C_{15}H_{23}O_2Si$ requires 263.1462, Δ -3.38 ppm.

IR (film) vmax/cm^{-1}: 2960, 1746, 1428, 1263, 834, 704.

1-((2*R*,3*R*,4*R*,5*S*)-4-(Dimethyl(phenyl)silyl)-3,5-dimethyltetrahydro-2H-pyran-2-yl)propan-2-one **11**

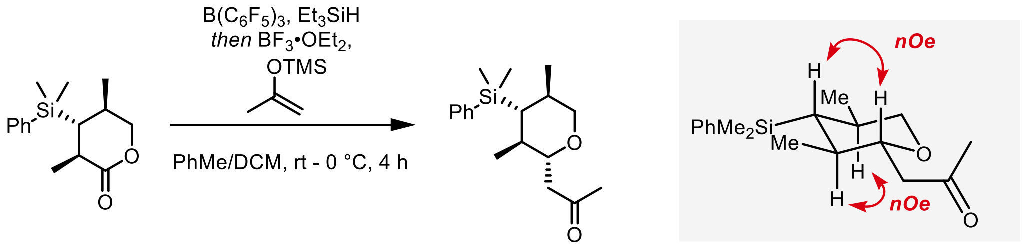

A 50 mL RBF was charged with tris(pentafluorophenyl)borane (41.0 mg, 0.0800 mmol, 0.0200 equiv.) in dry PhMe (2.0 mL). Triethylsilane (0.70 mL, 4.4 mmol, 1.1 equiv.) was added followed by lactone **9** (1.05 g, 4.00 mmol, 1.00 equiv.) in PhMe (2.0 mL). The resulting mixture was then stirred for 2 h at rt before being cooled to 0 °C. Isopropenyloxytrimethylsilane (1.0 mL, 6.0 mmol, 1.5 equiv.) in dry DCM (12 mL) was added followed by boron trifluoride diethyl etherate (0.54 mL, 4.4 mmol, 1.1 equiv.) and the resulting mixture was stirred at 0 °C for 2 h. The reaction was quenched with saturated aqueous sodium bicarbonate and extracted with DCM (3 x 20 mL). The combined organic layers were dried over Na_2SO_4 and concentrated *in vacuo*. Purification by FCC (15% Et_2O/Pentane) afforded the title compound as a colourless oil (858 mg, 2.82 mmol, 71% yield).

^{1}H NMR (500 MHz, $CDCl_3$) **δ** 7.55 – 7.47 (m, 2H), 7.38 – 7.30 (m, 3H), 3.77 (dd, *J* = 11.5, 5.0 Hz, 1H), 3.30 (td, *J* = 9.5, 3.0 Hz, 1H), 2.90 (t, *J* = 11.0 Hz, 1H), 2.58 (dd, *J* = 15.0, 3.0 Hz, 1H), 2.48 (dd, *J* = 15.0, 9.0 Hz, 1H), 2.17 (s, 3H), 1.83 – 1.70 (m, 1H), 1.52 – 1.40 (m, 1H), 0.72 (d, *J* = 6.5 Hz, 3H), 0.68 – 0.59 (m, 4H), 0.36 (s, 6H).

^{13}C NMR (126 MHz, $CDCl_3$) **δ** 208.4, 140.4, 133.8, 128.9, 128.0, 81.0, 75.3, 48.3, 36.8, 35.5, 31.4, 31.1, 19.0, 18.1, -0.8, -0.9.

$[\alpha]_D^{20}$ = +21.7 (c = 1.0, $CHCl_3$).

HRMS (ESI+): Found [M+H]+ = 305.1920; $C_{18}H_{29}O_2Si$ requires 305.1931, Δ -3.73 ppm.

IR (film) vmax/cm^{-1}: 2955, 1718, 1428, 1124, 837, 703.

1-((3*S*,4*S*,5*R*)-4-(Dimethyl(phenyl)silyl)-3,5-dimethylcyclohex-1-en-1-yl)ethan-1-one **12**

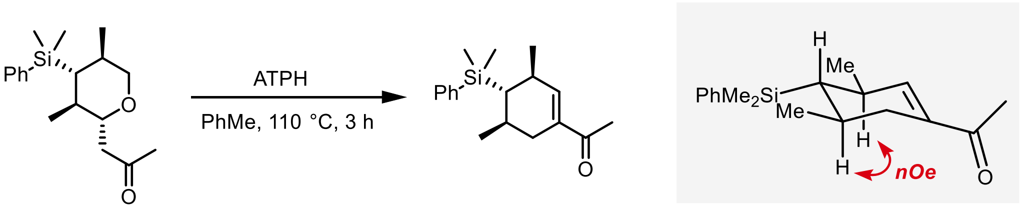

A 100 mL two-necked RBF, fitted with a reflux condenser, was charged with 2,6-diphenylphenol (4.85 g, 19.7 mmol, 3.04 equiv.) in dry PhMe (33 mL). Trimethylaluminium (2.0 M in PhMe, 3.3 mL, 6.6 mmol, 1.0 equiv.) was added and the mixture was stirred at rt for 30 minutes. Tetrahydropyran **11** (1.97 g, 6.47 mmol, 1.00 equiv.) was then added and the mixture was heated at 110 °C for 3 h. The mixture was then filtered through a plug of silica, washing with Et_2O, and concentrated *in vacuo*. Purification by MPLC (PuriFlash® 0-10% Et_2O/Pentane) afforded the title compound as a pale yellow oil (1.13 g, 3.94 mmol, 61% yield).

^{1}H NMR (400 MHz, $CDCl_3$) **δ** 7.56 – 7.46 (m, 2H), 7.40 – 7.29 (m, 3H), 6.73 (dd, *J* = 4.0, 1.5 Hz, 1H), 2.56 – 2.43 (m, 1H), 2.32 – 2.23 (m, 4H), 1.90 – 1.74 (m, 2H), 1.03 (d, *J* = 7.0 Hz, 3H), 0.94 (d, *J* = 6.5 Hz, 3H), 0.75 – 0.66 (m, 1H), 0.35 – 0.27 (m, 6H).

^{13}C NMR (101 MHz, $CDCl_3$) **δ** 199.5, 146.1, 139.3, 137.6, 133.9, 129.0, 127.9, 34.6, 32.0, 30.4, 29.4, 25.3, 23.8, 23.6, -2.7, -3.0.

$[\alpha]_D^{20}$ = -13.0 (c = 1.0, $CHCl_3$).

HRMS (ESI+): Found [M+H]+ = 287.1823; $C_{18}H_{27}OSi$ requires 287.1826, Δ -0.95 ppm.

IR (film) vmax/cm^{-1}: 2956, 1660, 1428, 1257, 1111, 835.

(*R*)-4-Benzyl-3-((1*S*,2*S*,6*R*,7*S*,8*S*,8a*S*)-4-((*tert*-butyldimethylsilyl)oxy)-7-(dimethyl(phenyl)silyl)-2,6,8-trimethyl-1,2,3,5,6,7,8,8a-octahydronaphthalene-1-carbonyl)oxazolidin-2-one ***cis-exo*-17** and (*R*)-4-Benzyl-3-((1*S*,2*R*,6*R*,7*S*,8*S*,8a*S*)-4-((*tert*-butyldimethylsilyl)oxy)-7-(dimethyl(phenyl)silyl)-2,6,8-trimethyl-1,2,3,5,6,7,8,8a-octahydronaphthalene-1-carbonyl)oxazolidin-2-one ***trans*-exo-17**

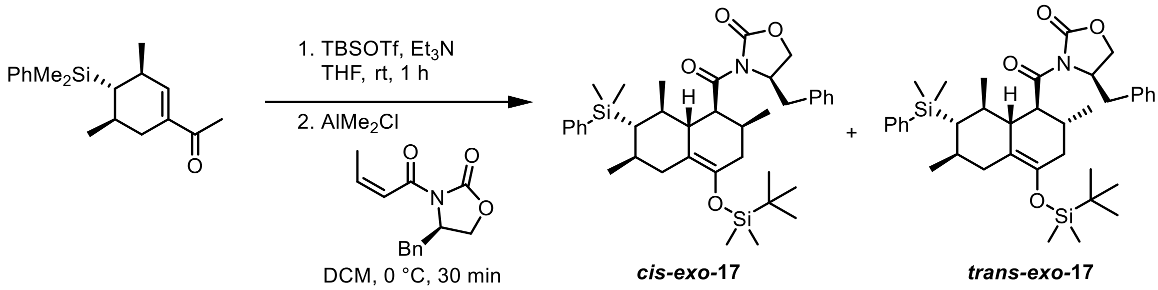

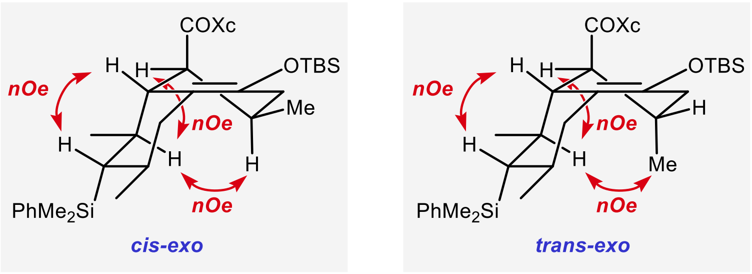

A 25 mL RBF was charged with cyclohexene **12** (200 mg, 0.698 mmol, 1.00 equiv.) in dry THF (3.5 mL). Triethylamine (0.25 mL, 1.8 mmol, 2.5 equiv.) was added followed by *tert*-butyldimethylsilyl trifluoromethanesulfonate (0.21 mL, 0.91 mmol, 1.3 equiv.) and the mixture was stirred for 1 h at rt. After this time, the mixture was diluted with pentane (50 mL) and washed with saturated aqueous sodium bicarbonate (25 mL) and water (25 mL). The combined organic layers were dried over Na_2SO_4 and concentrated *in vacuo*.

A 1.0 M solution of $AlMe_2Cl$ in PhMe was prepared by the addition of $AlMe_3$ (2.0 M in PhMe, 1.0 mL, 2.0 mmol) to a suspension of $AlCl_3$ (133 mg, 1.0 mmol) in dry PhMe (2 mL) at 0 °C. The solution was allowed to stir for 1 h at rt and used directly in the Diels-Alder reaction.

The crude silyl enol ether and oxazolidinone **(*R*)-16** (206 mg, 0.838 mmol, 1.20 equiv.) were dissolved in dry DCM (3.5 mL) and cooled to 0 °C. $AlMe_2Cl$ (1.0 M in PhMe, 1.4 mL, 2.0 equiv.) was added and the mixture was stirred at 0 °C for 30 min. Pyridine (0.5 mL) was then added and the mixture was filtered through a plug of silica, flushing with Et_2O (30 mL), and concentrated *in vacuo*. Purification by FCC (6-8% Et_2O/Pentane) afforded ***cis-exo*-17** as a colourless oil (269 mg, 0.417 mmol, 60% yield), and ***trans-exo*-17** as a colourless oil (104 mg, 0.161 mmol, 23% yield).

Data for (*R*)-4-Benzyl-3-((1*S*,2*S*,6*R*,7*S*,8*S*,8a*S*)-4-((*tert*-butyldimethylsilyl)oxy)-7-(dimethyl(phenyl)silyl)-2,6,8-trimethyl-1,2,3,5,6,7,8,8a-octahydronaphthalene-1-carbonyl)oxazolidin-2-one ***cis-exo*-17**

^{1}H NMR (400 MHz, $CDCl_3$) **δ** 7.55 – 7.46 (m, 2H), 7.37 – 7.16 (m, 8H), 4.72 – 4.62 (m, 1H), 4.19 – 4.07 (m, 2H), 3.94 (dd, *J* = 5.5, 4.0 Hz, 1H), 3.30 (dd, *J* = 13.5, 3.5 Hz, 1H), 2.80 (dd, *J* = 13.0, 5.0 Hz, 1H), 2.59 (dd, *J* = 13.0, 10.0 Hz, 1H), 2.32 – 2.19 (m, 2H, H_5), 2.19 – 2.09 (m, 1H), 2.01 – 1.90 (m, 1H), 1.70 – 1.59 (m, 1H), 1.50 – 1.39 (m, 1H), 1.39 – 1.29 (m, 1H), 0.97 – 0.84 (m, 18H), 0.74 (t, *J* = 10.5 Hz, 1H), 0.36 – 0.28 (m, 6H), 0.17 – 0.09 (m, 6H).

^{13}C NMR (101 MHz, $CDCl_3$) **δ** 174.9, 153.4, 141.2, 139.2, 135.7, 133.8, 129.6, 129.1, 128.6, 127.8, 127.4, 115.6, 65.9, 55.6, 45.4, 44.8, 40.5, 38.9, 37.9, 36.8, 36.4, 33.4, 29.4, 26.0, 25.0, 23.3, 18.4, 16.7, -0.7, -0.9, -3.7, -3.8.

$[\alpha]_D^{20}$ = +4.6 (c = 0.69, $CHCl_3$).

HRMS (ESI+): Found [M+H]+ = 646.3748; $C_{38}H_{56}NO_4Si_2$ requires 646.3742, Δ 0.86 ppm.

IR (film) vmax/cm^{-1}: 2956, 1779, 1692, 1457, 1208, 837, 702.

The relative stereochemistry was determined by NOESY analysis

Data for (*R*)-4-Benzyl-3-((1*S*,2*R*,6*R*,7*S*,8*S*,8a*S*)-4-((*tert*-butyldimethylsilyl)oxy)-7-(dimethyl(phenyl)silyl)-2,6,8-trimethyl-1,2,3,5,6,7,8,8a-octahydronaphthalene-1-carbonyl)oxazolidin-2-one ***trans-exo*-17**

^{1}H NMR (400 MHz, $CDCl_3$) **δ** 7.55 – 7.46 (m, 2H), 7.37 – 7.22 (m, 8H), 4.72 – 4.62 (m, 1H), 4.17 – 4.05 (m, 2H), 3.70 – 3.61 (m, 1H), 3.41 (dd, *J* = 13.0, 3.5 Hz, 1H), 2.71 (dd, *J* = 13.5, 4.5 Hz, 1H), 2.55 – 2.44 (m, 2H), 2.04 – 1.89 (m, 3H), 1.66 – 1.55 (m, 1H), 1.55 – 1.45 (m, 1H), 1.45 – 1.34 (m, 1H), 0.96 – 0.91 (m, 12H), 0.90 – 0.84 (m, 6H), 0.67 (dd, *J* = 11.0, 9.5 Hz, 1H), 0.36 – 0.28 (m, 6H), 0.14 – 0.06 (m, 6H).

^{13}C NMR (101 MHz, $CDCl_3$) **δ** 174.9, 150.4, 137.9, 136.7, 132.7, 130.7, 126.4, 126.0, 125.5, 124.7, 124.3, 113.8, 62.8, 53.2, 46.7, 45.3, 37.8, 35.7, 35.3, 34.5, 32.4, 31.8, 29.6, 22.8, 21.8, 20.1, 15.6, 15.2, -4.0, -4.1, -6.7, -7.1.

$[\alpha]_D^{20}$ = -24.8 (c = 1.0, $CHCl_3$).

HRMS (ESI+): Found [M+H]+ = 646.3733; $C_{38}H_{56}NO_4Si_2$ requires 646.3742, Δ -1.46 ppm.

IR (film) vmax/cm^{-1}: 2956, 1780, 1697, 1456, 1383, 1207, 703.

S-Ethyl (1*S*,2*S*,4a*S*,6*R*,7*S*,8*S*,8a*R*)-7-(dimethyl(phenyl)silyl)-2,6,8-trimethyl-4-oxodecahydronaphthalene-1-carbothioate **18**

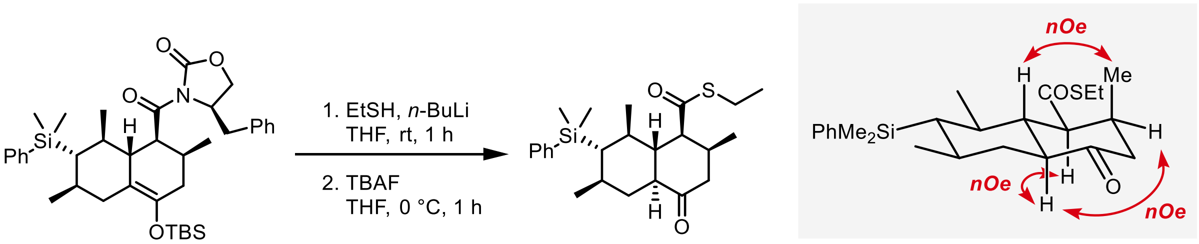

A 50 mL RBF was charged with ethanethiol (0.31 mL, 4.1 mmol, 3.0 equiv.) in dry THF (10 mL). *N*-BuLi (2.5 M in hexanes, 1.1 mL, 2.8 mmol, 2.0 equiv.) was added and the mixture was stirred at rt for 15 min. ***Cis-exo*-17** (891 mg, 1.38 mmol, 1.00 equiv.) in THF (4 mL) was added, and the mixture was stirred at rt for 1 h. The mixture was then filtered through a plug of silica, flushing with 1:1 DCM/Pentane (50 mL) and concentrated *in vacuo* to afford the crude thioester.

The crude thioester was dissolved in dry THF (14 mL) at 0 °C. TBAF (1.0 M in THF, 1.7 mL, 1.7 mmol, 1.2 equiv.) was added and the mixture was stirred at 0 °C for 1 h. The reaction was quenched with H_2O (5 mL) and extracted with DCM (3 x 10 mL). The combined organic layers were dried over Na_2SO_4 and concentrated *in vacuo*. Purification by FCC (10% Et_2O/Pentane) afforded the title compound as a colourless oil (370 mg, 0.888 mmol, 64% yield).

^{1}H NMR (600 MHz, $CDCl_3$) **δ** 7.53 – 7.47 (m, 2H), 7.37 – 7.30 (m, 3H), 2.85 (q, *J* = 7.5 Hz, 2H), 2.77 (t, *J* = 5.0 Hz, 1H), 2.54 – 2.44 (m, 1H), 2.35 (dd, *J* = 18.0, 12.0 Hz, 1H), 2.25 (dd, *J* = 18.0, 4.5 Hz, 1H), 2.17 – 2.07 (m, 2H), 1.59 – 1.45 (m, 3H), 1.22 (t, *J* = 7.5 Hz, 3H), 1.04 (d, *J* = 7.0 Hz, 3H), 0.93 (d, *J* = 5.5 Hz, 3H), 0.90 – 0.83 (m, 4H), 0.55 (t, *J* = 11.0 Hz, 1H), 0.35 (s, 6H).

^{13}C NMR (151 MHz, $CDCl_3$) **δ** 212.3, 202.3, 140.9, 133.8, 128.8, 127.9, 58.5, 49.1, 48.3, 43.3, 41.0, 39.3, 35.7, 32.3, 30.2, 24.2, 23.9, 23.0, 18.6, 14.8, -0.47, -0.54.

$[\alpha]_D^{20}$ = +37.5 (c = 0.87, $CHCl_3$).

HRMS (ESI+): Found [M+Na]+ = 439.2098; $C_{24}H_{36}O_2SSiNa$ requires 439.2098, Δ 0.10 ppm.

IR (film) vmax/cm^{-1}: 2964, 1779, 1716, 1679, 1250, 989, 702.

Methyl (2*E*,4*E*)-5-((1*R*,2*S*,4a*S*,6*R*,7*S*,8*S*,8a*R*)-7-(dimethyl(phenyl)silyl)-2,6,8-trimethyl-4-oxodecahydronaphthalen-1-yl)penta-2,4-dienoate **20**

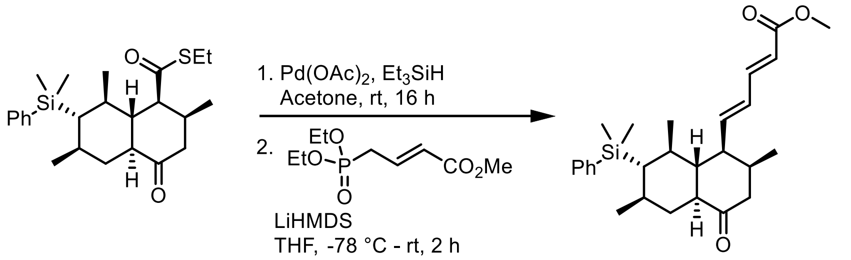

A 2-5 mL microwave vial was charged with thioester **18** (41.7 mg, 0.100 mmol, 1.00 equiv.) in acetone (1.0 mL). Palladium (II) acetate (4.5 mg, 0.020 mmol, 0.20 equiv.) was added and the mixture was purged with argon for 5 min. Triethylsilane (0.048 mL, 0.30 mmol, 3.0 equiv.) was added and the mixture was stirred at rt for 16 h, before being filtered through a plug of silica, washing with Et_2O, and concentrated *in vacuo* to afford the crude aldehyde.

A 2-5 mL microwave vial was charged with phosphonate **19** (71.0 mg, 0.300 mmol, 3.0 equiv.) in dry THF (0.5 mL) at -78 °C. LiHMDS (1.0 M in THF, 0.25 mL, 0.25 mmol, 2.5 equiv.) was added and the mixture was stirred at -78 °C for 30 min. The crude aldehyde in dry THF (0.5 mL) was added and the mixture was warmed to rt and stirred for 2 h. The reaction was quenched with saturated aqueous ammonium chloride and extracted with DCM (3 x 5 mL). The combined organic layers were dried over Na_2SO_4 and concentrated *in vacuo*. Purification by FCC (20% Et_2O/Pentane) afforded the title compound as a colourless oil (34.2 mg, 0.0780 mmol, 78% yield).

^{1}H NMR (400 MHz, $CDCl_3$) **δ** 7.55 – 7.45 (m, 2H), 7.37 – 7.28 (m, 3H), 7.21 (dd, *J* = 15.5, 11.0 Hz, 1H), 6.11 (dd, *J* = 15.0, 11.0 Hz, 1H), 5.94 (dd, *J* = 15.0, 10.0 Hz, 1H), 5.80 (d, *J* = 15.5 Hz, 1H), 3.73 (s, 3H), 2.50 – 2.28 (m, 3H), 2.20 (td, *J* = 11.5, 2.5 Hz, 1H), 2.09 – 1.96 (m, 2H), 1.65 – 1.41 (m, 2H), 1.01 (ddd, *J* = 12.0, 10.0, 5.0 Hz, 1H), 0.92 – 0.76 (m, 10H), 0.53 (t, *J* = 11.5 Hz, 1H), 0.39 – 0.31 (m, 6H).

^{13}C NMR (101 MHz, $CDCl_3$) **δ** 213.3, 167.6, 145.3, 144.7, 140.8, 133.8, 129.6, 128.8, 127.9, 119.9, 51.7, 50.6, 49.7, 48.6, 44.1, 40.2, 39.5, 35.5, 32.4, 31.6, 24.3, 23.3, 18.0, -0.6, -0.7.

$[\alpha]_D^{20}$ = +109.1 (c = 0.066, $CHCl_3$).

HRMS (ESI+): Found [M+Na]+ = 461.2484; $C_{27}H_{38}O_3SiNa$ requires 461.2482, Δ 0.33 ppm.

IR (film) vmax/cm^{-1}: 2955, 1717, 1640, 1262, 1004, 737, 703.

Methyl (2*E*,4*E*)-5-((1*S*,2*S*,4a*R*,6*R*,7*S*,8*S*,8a*S*)-7-(dimethyl(phenyl)silyl)-2,6,8-trimethyl-1,2,4a,5,6,7,8,8a-octahydronaphthalen-1-yl)penta-2,4-dienoate **21**

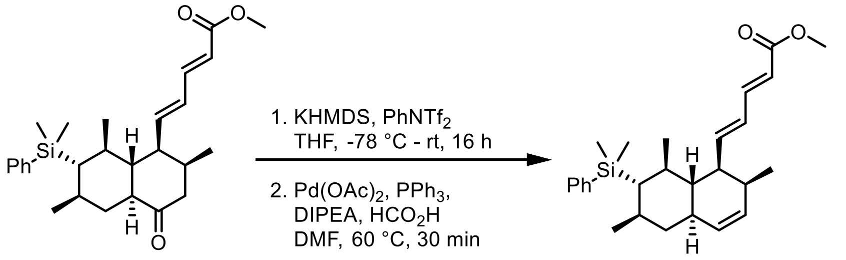

A 2-5 mL microwave vial was charged with ketone **20** (34.0 mg, 0.0775 mmol, 1.00 equiv.) in dry THF (0.5 mL) at -78 °C. KHMDS (0.5 M in PhMe, 0.31 mL, 0.16 mmol, 2.0 equiv.) was added and the mixture was stirred for 30 min, before $PhNTf_2$ (55.4 mg, 0.155 mmol, 2.00 equiv.) in THF (0.5 mL) was added, and the mixture was allowed to warm to rt and stirred for 16 h. The reaction was quenched with saturated aqueous ammonium chloride and extracted with DCM (3 x 5 mL). The combined organic layers were dried over Na_2SO_4 and concentrated *in vacuo* to afford the crude vinyl triflate.

The crude enol triflate was dissolved in DMF (0.2 mL) and a solution of Palladium (II) acetate (1.7 mg, 0.0078 mmol, 0.10 equiv), triphenylphosphine (4.1 mg, 0.016 mmol, 0.20 equiv.) and DIPEA (0.054 mL, 0.31 mmol, 4.0 equiv.) in DMF (0.2 mL) was added, followed by Formic acid (0.009 mL, 0.2 mmol, 3 equiv.) in DMF (0.1 mL). The resulting mixture was warmed to 60 °C and stirred for 30 min, after which time the mixture was diluted with Et_2O (1 mL) and filtered through a plug of silica, then concentrated *in vacuo*. Purification by FCC (10% Et_2O/Pentane) afforded the title compound as a colourless oil (18.8 mg, 0.0445 mmol, 57% yield).

^{1}H NMR (400 MHz, $CDCl_3$) **δ** 7.53 – 7.47 (m, 2H), 7.34 – 7.29 (m, 3H), 7.26 (dd, *J* = 15.5, 10.5 Hz, 1H), 6.18 (dd, *J* = 15.0, 10.5 Hz, 1H), 6.08 (dd, *J* = 15.0, 10.5 Hz, 1H), 5.79 (d, *J* = 15.5 Hz, 1H), 5.56 (ddd, *J* = 9.5, 4.5, 2.5 Hz, 1H), 5.43 (dt, *J* = 9.5, 2.0 Hz, 1H), 3.74 (s, 3H), 2.37 – 2.27 (m, 1H), 2.26 – 2.15 (m, 1H), 1.72 – 1.53 (m, 3H), 1.51 – 1.38 (m, 1H), 0.98 – 0.87 (m, 4H), 0.84 – 0.77 (m, 7H), 0.55 (t, *J* = 11.0 Hz, 1H), 0.31 (d, *J* = 6.5 Hz, 6H).

^{13}C NMR (101 MHz, $CDCl_3$) **δ** 168.0, 149.4, 145.5, 141.1, 133.9, 132.7, 132.6, 128.6, 127.8, 127.3, 118.8, 51.6, 50.8, 48.9, 43.2, 41.7, 40.0, 36.4, 36.0, 33.7, 25.9, 24.4, 16.8, -0.7, -1.2.

$[\alpha]_D^{20}$ = +120.0 (c = 0.040, $CHCl_3$).

HRMS (ESI+): Found [M+H]+ = 423.2721; $C_{27}H_{39}O_2Si$ requires 423.2714, Δ 1.68 ppm.

IR (film) vmax/cm^{-1}: 2883, 1618, 1519, 1335, 1312, 737, 702.

Methyl (2*E*,4*E*)-5-((1*S*,2*S*,4a*R*,6*R*,7*S*,8*S*,8a*S*)-7-hydroxy-2,6,8-trimethyl-1,2,4a,5,6,7,8,8a-octahydronaphthalen-1-yl)penta-2,4-dienoate **(+)-Coprophilin (1)**

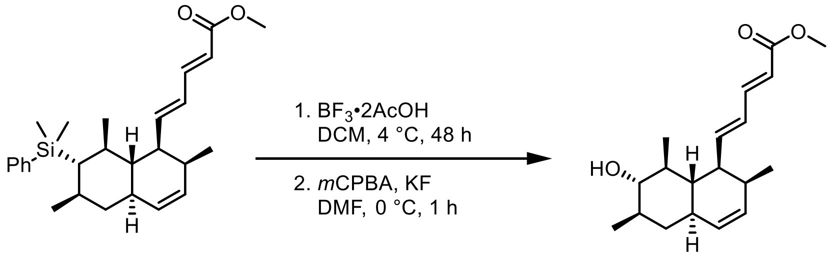

A 2 mL vial was charged with triene **21** (10.7 mg, 0.0253 mmol, 1.00 equiv.) in DCM (0.5 mL). BF_3.2AcOH (0.018 mL, 0.125 mmol, 5.0 equiv.) was added at 0 °C and the mixture was stirred for 48 h at 4 °C. The reaction was quenched with saturated aqueous sodium bicarbonate and extracted with DCM (3 x 1 mL). The combined organic layers were dried over Na_2SO_4 and concentrated *in vacuo* to afford the crude silyl fluoride.

The crude silyl fluoride was dissolved in DMF (0.2 mL) at 0 °C. KF (8.8 mg, 0.152 mmol, 6.0 equiv.) was added followed by *m*CPBA (77 wt.%, 5.7 mg, 0.025 mmol, 1.0 equiv.) in DMF (0.1 mL) and the mixture was stirred at 0 °C for 1 h, after which the reaction mixture was directly loaded onto a column of silica. Purification by FCC (20% EtOAc/Pentane) afforded the title compound (3.1 mg, 0.010 mmol, 40% yield).

^{1}H NMR (600 MHz, CDCl3) **δ** 7.29 (dd, *J* = 15.5, 11.0 Hz, 1H), 6.19 (dd, *J* = 15.0, 10.5 Hz, 1H), 6.10 (dd, *J* = 15.0, 11.0 Hz, 1H), 5.79 (d, *J* = 15.5 Hz, 1H), 5.58 (ddd, *J* = 9.5, 4.5, 2.5 Hz, 1H), 5.46 (dt, *J* = 9.5, 2.0 Hz, 1H), 3.74 (s, 3H), 2.73 (t, *J* = 9.5 Hz, 1H), 2.45 (ddd, *J* = 10.5, 9.0, 5.5 Hz, 1H), 2.25 – 2.16 (m, 1H), 1.90 – 1.82 (m, 1H), 1.76 (dt, *J* = 13.0, 3.5 Hz, 1H), 1.53 – 1.43 (m, 1H), 1.35 – 1.26 (m, 1H), 1.11 – 1.02 (m, 7H), 1.00 – 0.93 (m, 4H).

^{13}C NMR (151 MHz, $CDCl_3$) **δ** 167.9, 149.8, 145.4, 132.8, 131.7, 127.0, 118.9, 82.3, 51.6, 49.6, 46.1, 44.0, 41.8, 39.7, 39.3, 36.5, 19.2, 18.0, 16.7.

$[\alpha]_D^{20}$ = +95.0 (c = 0.20, MeOH). **Lit $[\alpha]_D^{22}$** = +96.0 (c = 0.92, MeOH).[6]

HRMS (ESI+): Found [M+H]+ = 305.2102; $C_{19}H_{29}O_3$ requires 305.2111, Δ -3.03 ppm.

IR (film) vmax/cm^{-1}: 3484 (broad), 2954, 1719, 1639, 1268, 1007, 736.

The spectroscopic data matched that previously reported in the literature (see Table 1).[6, 7]

tert-Butyl (2*E*,4*E*)-5-((1*R*,2*S*,4a*S*,6*R*,7*S*,8*S*,8a*R*)-7-(dimethyl(phenyl)silyl)-2,6,8-trimethyl-4-oxodecahydronaphthalen-1-yl)-2-methylpenta-2,4-dienoate **23**

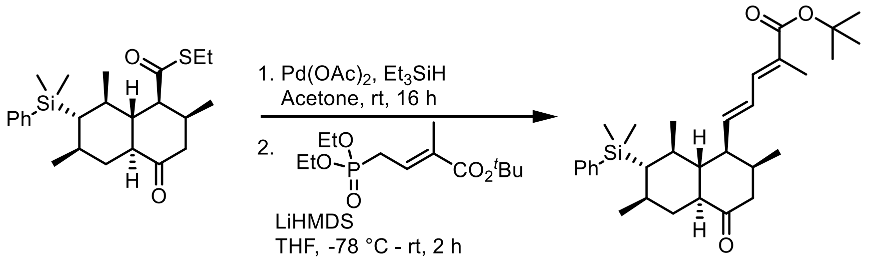

A 2-5 mL microwave vial was charged with thioester **18** (178 mg, 0.427 mmol, 1.00 equiv.) in acetone (4.0 mL). Palladium (II) acetate (19.2 mg, 0.0854 mmol, 0.200 equiv.) was added and the mixture was degassed with Argon. Triethylsilane (0.20 mL, 1.3 mmol, 3.0 equiv.) was added and the mixture was stirred at rt for 16 h, before being filtered through a plug of silica, washing with Et_2O, and concentrated *in vacuo* to afford the crude aldehyde.

A 2-5 mL microwave vial was charged with phosphonate **22** (374 mg, 1.28 mmol, 3.00 equiv.) in dry THF (3.0 mL) at -78 °C. LiHMDS (1.0 M in THF, 1.1 mL, 1.1 mmol, 2.5 equiv.) was added and the mixture was stirred at -78 °C for 30 min. The crude aldehyde in dry THF (1.0 mL) was added and the mixture was slowly warmed to rt and stirred for 2 h. The reaction was quenched with saturated aqueous ammonium chloride and extracted with DCM (3 x 10 mL). The combined organic layers were dried over Na_2SO_4 and concentrated *in vacuo*. Purification by FCC (15% Et_2O/Pentane) afforded the title compound as a colourless oil (152 mg, 0.307 mmol, 72% yield).

^{1}H NMR (400 MHz, $CDCl_3$) **δ** 7.54 – 7.46 (m, 2H), 7.37 – 7.30 (m, 3H), 7.01 (d, *J* = 11.0 Hz, 1H), 6.25 (dd, *J* = 15.0, 11.0 Hz, 1H), 5.84 (dd, *J* = 15.0, 10.0 Hz, 1H), 2.50 – 2.29 (m, 3H), 2.21 (td, *J* = 11.5, 2.5 Hz, 1H), 2.11 – 1.98 (m, 2H), 1.87 (d, *J* = 1.5 Hz, 3H), 1.66 – 1.58 (m, 1H), 1.55 – 1.45 (m, 10H), 1.07 – 0.96 (m, 1H), 0.93 – 0.79 (m, 10H), 0.53 (t, *J* = 11.5 Hz, 1H), 0.39 – 0.31 (m, 6H).

^{13}C NMR (101 MHz, $CDCl_3$) **δ** 213.7, 167.9, 142.8, 140.9, 137.1, 133.8, 128.8, 127.9, 127.8, 127.4, 80.3, 51.0, 49.8, 48.7, 44.2, 40.3, 39.5, 35.6, 32.4, 31.5, 28.3, 24.3, 23.3, 18.0, 12.8, -0.6, -0.7.

$[\alpha]_D^{20}$ = +52.9 (c = 0.51, $CHCl_3$).

HRMS (ESI+): Found [M+Na]+ = 517.3125; $C_{31}H_{46}O_3SiNa$ requires 517.3108, Δ 3.20 ppm.

IR (film) vmax/cm^{-1}: 2974, 1699, 1636, 1254, 1067, 701.

tert-Butyl (2*E*,4*E*)-5-((1*S*,2*S*,4a*R*,6*R*,7*S*,8*S*,8a*S*)-7-(dimethyl(phenyl)silyl)-2,6,8-trimethyl-1,2,4a,5,6,7,8,8a-octahydronaphthalen-1-yl)-2-methylpenta-2,4-dienoate **24**

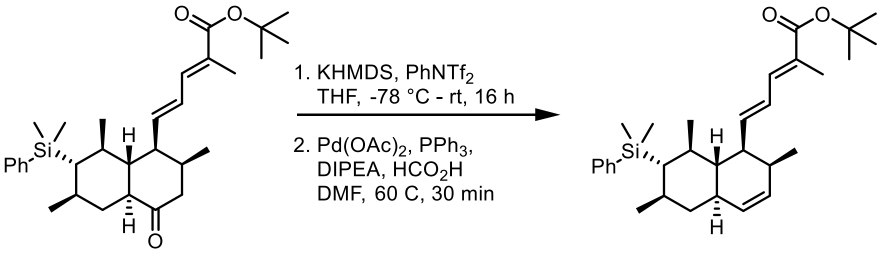

A 2 mL vial was charged with ketone **23** (20.0 mg, 0.0404 mmol, 1.00 equiv.) in dry THF (0.3 mL) at -78 °C. KHMDS (0.5 M in PhMe, 0.16 mL, 0.080 mmol, 2.0 equiv.) was added and the mixture was stirred for 30 min, before $PhNTf_2$ (28.9 mg, 0.808 mmol, 2.00 equiv.) in THF (0.2 mL) was added, and the mixture was allowed to warm to rt and stirred for 16 h. The reaction was quenched with saturated aqueous ammonium chloride and extracted with DCM (3 x 5 mL). The combined organic layers were dried over Na_2SO_4 and concentrated *in vacuo* to afford the crude vinyl triflate.

The crude enol triflate was dissolved in DMF (0.2 mL) and a solution of palladium (II) acetate (0.9 mg, 0.008 mmol, 0.1 equiv), triphenylphosphine (2.1 mg, 0.0081 mmol, 0.20 equiv.) and DIPEA (0.028 mL, 0.16 mmol, 4.0 equiv.) in DMF (0.2 mL) was added, followed by formic acid (0.005 mL, 0.1 mmol, 3 equiv.) in DMF (0.1 mL). The resulting mixture was warmed to 60 °C and stirred for 30 min, after which time the mixture was diluted with Et_2O (1 mL) and filtered through a plug of silica, then concentrated *in vacuo*. Purification by FCC (3% Et_2O/Pentane) afforded the title compound as a colourless oil (9.2 mg, 0.019 mmol, 48% yield).

^{1}H NMR (500 MHz, $CDCl_3$) **δ** 7.54 – 7.47 (m, 2H), 7.35 – 7.29 (m, 3H), 7.05 (d, *J* = 11.0 Hz, 1H), 6.22 (dd, *J* = 15.0, 11.0 Hz, 1H), 6.08 (dd, *J* = 15.0, 10.5 Hz, 1H), 5.57 (ddd, *J* = 9.5, 4.5, 2.5 Hz, 1H), 5.42 (dt, *J* = 9.5, 2.0 Hz, 1H), 2.39 – 2.30 (m, 1H), 2.24 – 2.18 (m, 1H), 1.89 (s, 3H), 1.72 – 1.54 (m, 3H), 1.53 – 1.44 (m, 10H), 0.99 – 0.91 (m, 4H), 0.87 – 0.75 (m, 7H), 0.56 (t, *J* = 11.0 Hz, 1H), 0.36 – 0.28 (m, 6H).

^{13}C NMR (126 MHz, $CDCl_3$) **δ** 168.2, 146.9, 141.2, 137.8, 133.9, 132.8, 132.6, 128.6, 127.7, 126.7, 125.1, 80.2, 51.0, 48.9, 43.3, 41.8, 40.0, 36.6, 36.0, 33.7, 28.3, 26.0, 24.4, 16.8, 12.8, -0.6, -1.2.

$[\alpha]_D^{20}$ = +66.2 (c = 0.55, $CHCl_3$).

HRMS (ESI+): Found [M+Na]+ = 501.3163; $C_{31}H_{46}O_2SiNa$ requires 501.3159, Δ 0.74 ppm.

IR (film) vmax/cm^{-1}: 2974, 1698, 1634, 1255, 1161, 759.

(2*E*,4*E*)-5-((1*S*,2*S*,4a*R*,6*R*,7*S*,8*S*,8a*S*)-7-Hydroxy-2,6,8-trimethyl-1,2,4a,5,6,7,8,8a-octahydronaphthalen-1-yl)-2-methylpenta-2,4-dienoic acid **(+)-Trichodermic acid (2)**

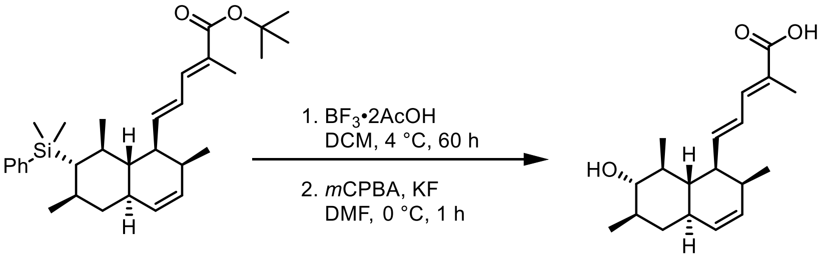

A 2 mL vial was charged with triene **24** (7.8 mg, 0.016 mmol, 1.0 equiv.) in DCM (0.5 mL). BF_3.2AcOH (0.012 mL, 0.080 mmol, 5.0 equiv.) was added at 0 °C and the mixture was stirred for 60 h at 4 °C. The reaction was quenched with water and extracted with $CHCl_3$ (3 x 1 mL). The combined organic layers were dried over Na_2SO_4 and concentrated *in vacuo* to afford the crude silyl fluoride.

The crude silyl fluoride was dissolved in DMF (0.2 mL) at 0 °C. KF (5.8 mg, 0.10 mmol, 6.0 equiv.) was added followed by *m*CPBA (77 wt.%, 3.7 mg, 0.016 mmol, 1.0 equiv.) in DMF (0.1 mL) and the mixture was stirred at 0 °C for 1 h, after which the reaction was quenched with saturated aqueous sodium thiosulfate and 3M aqueous HCl. The mixture was extracted with $CHCl_3$ (3 x 1 mL), dried over Na_2SO_4 and concentrated *in vacuo*. Purification by FCC (2% $HCO_2H/CHCl_3$) afforded the title compound (2.1 mg, 0.069 mmol, 43% yield).

^{1}H NMR (600 MHz, $CDCl_3$) **δ** 7.30 (dq, *J* = 11.0, 1.5 Hz, 1H), 6.28 (dd, *J* = 15.0, 11.0 Hz, 1H), 6.20 (dd, *J* = 15.0, 10.5 Hz, 1H), 5.59 (ddd, *J* = 9.5, 4.5, 3.0 Hz, 1H), 5.46 (dt, *J* = 9.5, 2.0 Hz, 1H), 2.74 (t, *J* = 9.5 Hz, 1H), 2.50 (ddd, *J* = 10.5, 9.0, 5.5 Hz, 1H), 2.22 (qt, *J* = 7.0, 5.0 Hz, 1H), 1.94 (d, *J* = 1.5 Hz, 3H), 1.88 (tq, *J* = 12.5, 2.5 Hz, 1H), 1.76 (dt, *J* = 13.0, 3.5 Hz, 1H), 1.55 – 1.44 (m, 1H), 1.37 – 1.28 (m, 1H), 1.12 – 1.06 (m, 4H), 1.04 (d, *J* = 6.5 Hz, 3H), 1.01 – 0.92 (m, 4H).

^{13}C NMR (151 MHz, $CDCl_3$) **δ** 172.9, 149.5, 140.9, 132.9, 131.6, 124.6, 124.2, 82.3, 50.0, 46.0, 44.0, 42.0, 39.7, 39.4, 36.7, 19.2, 18.2, 16.7, 12.5.

$[\alpha]_D^{20}$ = +50.8 (c = 0.12, MeOH). **Lit $[\alpha]_D^{24}$** = +56.4 (c = 0.59, MeOH).[8]

HRMS (ESI+): Found [M+H]+ = 305.2113; $C_{19}H_{29}O_3$ requires 305.2111, Δ 0.59 ppm.

IR (film) vmax/cm^{-1}: 3374 (broad), 2923, 1682, 1635, 1541, 1258, 988.

The spectroscopic data matched that previously reported in the literature (see Table 2).[8]

tert-Butyl (2*E*,4*E*)-5-((1*R*,4a*S*,6*R*,7*S*,8*S*,8a*R*)-7-(dimethyl(phenyl)silyl)-2,6,8-trimethyl-4-oxo-1,4,4a,5,6,7,8,8a-octahydronaphthalen-1-yl)-2-methylpenta-2,4-dienoate **25**

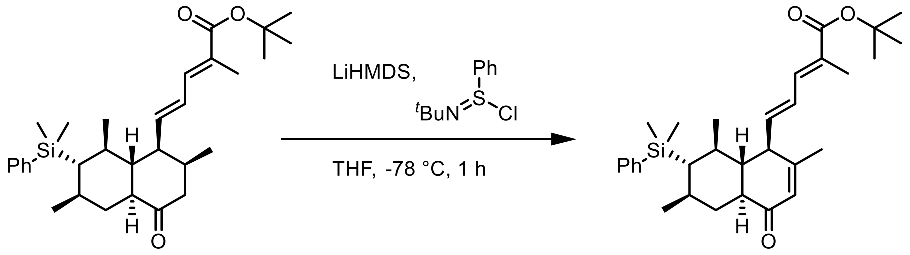

A 2-5 mL microwave vial was charged with ketone **23** (19.6 mg, 0.0396 mmol, 1.00 equiv.) in dry THF (0.3 mL) at -78 °C. LiHMDS (1.0 M in THF, 0.079 mL, 0.079 mmol, 2.0 equiv.) was added and the mixture was stirred for 30 min. N-tert-Butylbenzenesulfinimidoyl chloride (17.1 mg, 0.0792 mmol, 2.00 equiv.) in THF (0.2 mL) was added and the mixture was stirred for 30 min at -78 °C. The reaction was quenched with saturated aqueous ammonium chloride and extracted with Et_2O (3 x 2 mL). The combined organic layers were dried over Na_2SO_4 and concentrated *in vacuo*. Purification by FCC (15% Et_2O/Pentane) afforded the title compound as a colourless oil (16.3 mg, 0.0331 mmol, 84% yield).

^{1}H NMR (400 MHz, $CDCl_3$) **δ** 7.55 – 7.47 (m, 2H), 7.36 – 7.29 (m, 3H), 7.03 (d, *J* = 11.5 Hz, 1H), 6.35 (dd, *J* = 15.0, 11.5 Hz, 1H), 5.93 (q, *J* = 1.5 Hz, 1H), 5.78 (dd, *J* = 15.0, 10.0 Hz, 1H), 2.81 (t, *J* = 9.5 Hz, 1H), 2.34 – 2.24 (m, 1H), 1.98 – 1.85 (m, 4H), 1.79 (s, 3H), 1.69 – 1.52 (m, 2H), 1.50 (s, 9H), 1.44 – 1.30 (m, 1H), 0.93 – 0.87 (m, 6H), 0.77 (dt, *J* = 13.5, 11.5 Hz, 1H), 0.56 (dd, *J* = 11.0, 9.5 Hz, 1H), 0.38 – 0.30 (m, 6H).

^{13}C NMR (101 MHz, $CDCl_3$) **δ** 200.4, 167.7, 159.4, 143.2, 140.5, 136.3, 133.9, 128.9, 128.8, 127.9, 126.8, 80.5, 53.0, 50.8, 48.8, 39.1, 38.1, 34.6, 32.1, 28.3, 26.9, 24.8, 23.6, 13.0, -1.1.

$[\alpha]_D^{20}$ = +179.6 (c = 1.0, $CHCl_3$).

HRMS (ESI+): Found [M+H]+ = 493.3136; $C_{31}H_{45}O_3Si$ requires 493.3132, Δ 0.71 ppm.

IR (film) vmax/cm^{-1}: 2976, 1699, 1674, 1252, 1109, 702.

(2*E*,4*E*)-5-((1*R*,4a*S*,6*R*,7*S*,8*S*,8a*S*)-7-Hydroxy-2,6,8-trimethyl-4-oxo-1,4,4a,5,6,7,8,8a-octahydronaphthalen-1-yl)-2-methylpenta-2,4-dienoic acid **(+)-Trichodermic acid E (5)**

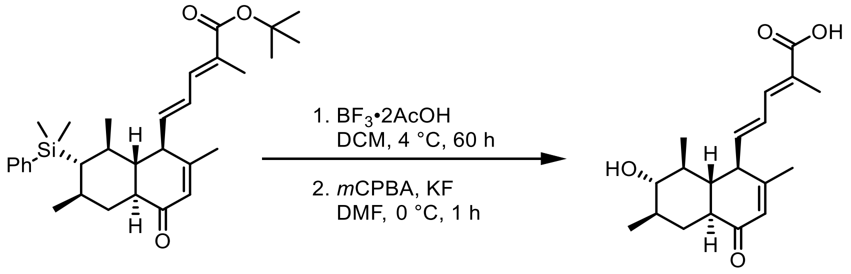

A 2 mL vial was charged with enone **25** (11.5 mg, 0.0233 mmol, 1.0 equiv.) in DCM (0.5 mL). BF_3.2AcOH (0.016 mL, 0.12 mmol, 5.0 equiv.) was added at 0 °C and the mixture was stirred for 60 h at 4 °C. The reaction was quenched with water and extracted with $CHCl_3$ (3 x 1 mL). The combined organic layers were dried over Na_2SO_4 and concentrated *in vacuo* to afford the crude silyl fluoride.

The crude silyl fluoride was dissolved in DMF (0.2 mL) at 0 °C. KF (8.1 mg, 0.14 mmol, 6.0 equiv.) was added followed by *m*CPBA (77 wt.%, 5.2 mg, 0.023 mmol, 1.0 equiv.) in DMF (0.1 mL) and the mixture was stirred at 0 °C for 1 h, after which the reaction was quenched with saturated aqueous sodium thiosulfate and 3M aqueous HCl. The mixture was extracted with $CHCl_3$ (3 x 1 mL), dried over Na_2SO_4 and concentrated *in vacuo*. Purification by FCC (3% $HCO_2H/CHCl_3$) followed by preparative TLC (2% MeOH, 2% HCO_2H in $CHCl_3$) afforded the title compound (3.3 mg, 0.0104 mmol, 45% yield).

^{1}H NMR (600 MHz, MeOD) **δ** 7.20 (d, *J* = 11.5 Hz, 1H), 6.62 (dd, *J* = 15.0, 11.5 Hz, 1H), 5.97 (dd, *J* = 15.0, 9.5 Hz, 1H), 5.94 (dd, *J* = 2.0, 1.5 Hz, 1H), 3.21 (t, *J* = 8.5 Hz, 1H), 2.61 (t, *J* = 10.0 Hz, 1H), 2.25 (td, *J* = 12.0, 3.5 Hz, 1H), 2.18 (dt, *J* = 14.0, 3.5 Hz, 1H), 1.95 (d, *J* = 1.5 Hz, 3H), 1.86 (t, *J* = 1.0 Hz, 3H), 1.61 (ddd, *J* = 12.5, 10.5, 8.5 Hz, 1H), 1.51 (tq, *J* = 10.0, 6.5 Hz, 1H), 1.41 (dddd, *J* = 12.5, 10.0, 6.5, 3.5 Hz, 1H), 1.12 (d, J = 6.5 Hz, 3H), 1.06 (d, *J* = 6.5 Hz, 3H), 0.94 (q, *J* = 12.5 Hz, 1H).

^{13}C NMR (151 MHz, MeOD) **δ** 202.4, 172.0, 162.4, 146.2, 138.8, 130.4, 128.4, 127.0, 81.3, 52.2, 49.8, 49.6, 47.9, 39.4, 33.3, 23.6, 19.6, 18.9, 12.9.

$[\alpha]_D^{20}$ = +122.3 (c = 0.25, MeOH). **Lit $[\alpha]_D$** = +126 (c = 0.1, MeOH).[9]

HRMS (ESI+): Found [M+H]+ = 319.1897; $C_{19}H_{27}O_4$ requires 319.1904, Δ -2.15 ppm.

IR (film) vmax/cm^{-1}: 3408 (broad), 2876, 1670, 1252, 1023, 981.

The spectroscopic data matched that previously reported in the literature (see Table 3).[9]

(2*E*,4*E*)-5-((1*R*,4*R*,4a*S*,6*R*,7*S*,8*S*,8a*S*)-4,7-Dihydroxy-2,6,8-trimethyl-1,4,4a,5,6,7,8,8a-octahydronaphthalen-1-yl)-2-methylpenta-2,4-dienoic acid **(+)-Trichodermic acid G (6)**

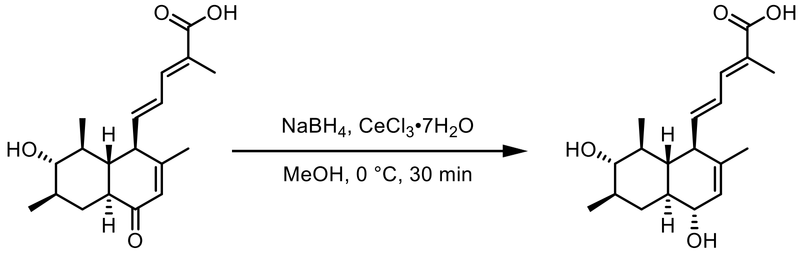

A 2 mL vial was charged with **(+)-Trichodermic acid E (5)** (2.0 mg, 0.0063 mmol, 1.0 equiv.) in MeOH (0.3 mL) at 0 °C. Cerium (III) chloride heptahydrate (12 mg, 0.032 mmol, 5.0 equiv.) was added followed by $NaBH_4$ (1.2 mg, 0.032 mmol, 5.0 equiv.) and the mixture was stirred at 0 °C for 30 min. The reaction was directly purified by preparative TLC (2% MeOH, 2% HCO_2H in $CHCl_3$) to afford the title compound (1.4 mg, 0.0044 mmol, 70% yield).

^{1}H NMR (600 MHz, MeOD) **δ** 7.15 (d, *J* = 11.5 Hz, 1H), 6.45 (dd, *J* = 15.0, 11.5 Hz, 1H), 5.79 (dd, *J* = 15.0, 9.5 Hz, 1H), 5.53 (q, *J* = 1.5 Hz, 1H), 3.69 – 3.62 (m, 1H), 2.68 (t, *J* = 8.0 Hz, 1H), 2.64 (t, *J* = 10.0 Hz, 1H), 2.22 (dt, *J* = 13.0, 3.5 Hz, 1H), 1.91 (d, J = 1.5 Hz, 3H), 1.60 (t, *J* = 2.0 Hz, 3H), 1.42 – 1.27 (m, 2H), 1.19 – 1.09 (m, 2H), 1.07 (d, *J* = 6.5 Hz, 3H), 1.03 (d, *J* = 6.5 Hz, 3H), 0.78 (td, *J* = 13.0, 11.0 Hz, 1H).

^{13}C NMR (151 MHz, MeOD) **δ** 172.2, 149.0, 139.7, 135.5, 130.7, 128.5, 126.8, 82.1, 72.1, 51.7, 48.8, 48.1, 46.9, 39.9, 37.4, 22.0, 19.6, 18.3, 12.7.

$[\alpha]_D^{20}$ = +226.0 (c = 0.10, MeOH). **Lit $[\alpha]_D$** = +235 (c = 0.1, MeOH).[9]

HRMS (ESI+): Found [M+Na]+ = 343.1878; $C_{19}H_{28}O_4Na$ requires 343.1880, Δ -0.53 ppm.

IR (film) vmax/cm^{-1}: 3380 (broad), 2927, 2875, 1681, 1376, 1262, 1048, 979.

The spectroscopic data matched that previously reported in the literature (see Table 4).[9]

tert-Butyl (2*E*,4*E*)-5-((1*R*,2*R*,3*S*,4*S*,4a*S*,6*R*,7*S*,8*S*,8a*R*)-7-(dimethyl(phenyl)silyl)-3,4-dihydroxy-2,6,8-trimethyldecahydronaphthalen-1-yl)-2-methylpenta-2,4-dienoate **26**

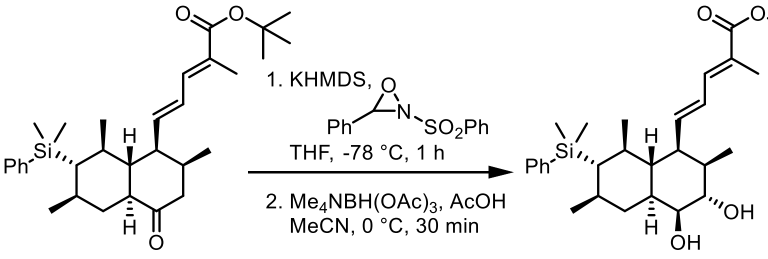

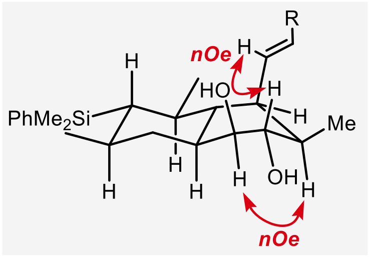

A 2 mL vial was charged with ketone **23** (10.0 mg, 0.0202 mmol, 1.00 equiv.) in dry THF (0.3 mL) at -78 °C. KHMDS (0.5 M in PhMe, 0.080 mL, 0.040 mmol, 2.0 equiv.) was added and the mixture was stirred for 30 min. 3-Phenyl-2-(phenylsulfonyl)-1,2-oxaziridine (10.6 mg, 0.0404 mmol, 2.00 equiv.) in dry THF (0.2 mL) was added and the mixture was stirred at -78 °C for 1 h. The reaction was quenched with saturated aqueous ammonium chloride and extracted with EtOAc (3 x 1 mL), dried over Na_2SO_4 and concentrated *in vacuo* to afford the crude hydroxy ketone.

The crude hydroxy ketone was dissolved in dry MeCN (0.2 mL) at 0 °C. Tetramethylammonium triacetoxyborohydride (21.3 mg, 0.0808 mmol, 4.00 equiv.) in dry MeCN (0.1 mL) was added followed by AcOH (0.030 mL) and the mixture was stirred at 0 °C for 30 min. The reaction was quenched with saturated aqueous sodium bicarbonate and extracted with EtOAc (3 x 1 mL), dried over Na_2SO_4 and concentrated *in vacuo*. Purification by FCC (30% EtOAc/Pentane) afforded the title compound as a colourless oil (6.5 mg, 0.013 mmol, 64% yield).

^{1}H NMR (600 MHz, $CDCl_3$) **δ** 7.55 – 7.47 (m, 2H), 7.38 – 7.29 (m, 3H), 7.04 (dd, *J* = 11.0, 1.5 Hz, 1H), 6.23 (dd, *J* = 15.0, 11.0 Hz, 1H), 6.10 (dd, *J* = 15.0, 10.0 Hz, 1H), 3.70 – 3.66 (m, 1H), 3.60 – 3.55 (m, 1H), 2.28 (dt, *J* = 10.5, 5.5 Hz, 1H), 1.87 (d, *J* = 1.5 Hz, 3H), 1.81 – 1.72 (m, 1H), 1.65 – 1.51 (m, 3H), 1.49 (s, 9H), 1.47 – 1.39 (m, 1H), 1.23 – 1.15 (m, 1H), 1.07 – 0.92 (m, 4H), 0.91 – 0.76 (m, 6H), 0.53 (t, *J* = 11.0 Hz, 1H), 0.37 – 0.29 (m, 6H).

^{13}C NMR (151 MHz, $CDCl_3$) **δ** 168.1, 145.5, 141.1, 137.7, 134.0, 128.7, 127.8, 127.0, 126.3, 80.2, 77.9, 77.6, 48.5, 44.1, 39.8, 39.6, 39.5, 39.0, 37.8, 33.5, 28.3, 24.7, 24.5, 15.3, 12.8, -0.6, -1.0.

$[\alpha]_D^{20}$ = +30.7 (c = 0.43, $CHCl_3$).

HRMS (ESI+): Found [M+Na]+ = 535.3211; $C_{31}H_{48}O_4SiNa$ requires 535.3214, Δ -0.58 ppm.

IR (film) vmax/cm^{-1}: 3447 (broad), 2953, 1698, 1256, 1108, 771.

(2*E*,4*E*)-2-Methyl-5-((1*R*,2*R*,3*S*,4*S*,4a*S*,6*R*,7*S*,8*S*,8a*S*)-3,4,7-trihydroxy-2,6,8-trimethyldecahydronaphthalen-1-yl)penta-2,4-dienoic acid **(-)-Trichodermic acid D (4)**

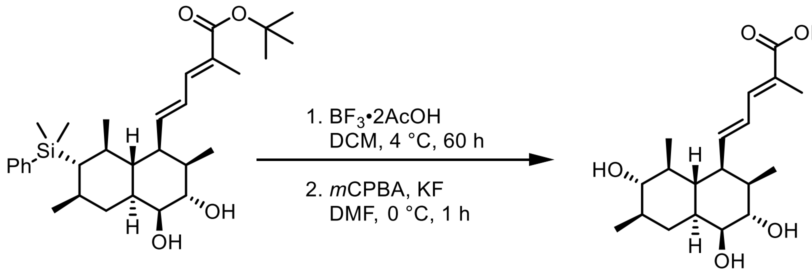

A 2 mL vial was charged with diol **26** (6.0 mg, 0.012 mmol, 1.0 equiv.) in DCM (0.5 mL). BF_3.2AcOH (0.007 mL, 0.06 mmol, 5 equiv.) was added at 0 °C and the mixture was stirred for 60 h at 4 °C. The reaction was quenched with water and extracted with $CHCl_3$ (3 x 1 mL). The combined organic layers were dried over Na_2SO_4 and concentrated *in vacuo* to afford the crude silyl fluoride.

The crude silyl fluoride was dissolved in DMF (0.2 mL) at 0 °C. KF (4.2 mg, 0.072 mmol, 6.0 equiv.) was added followed by *m*CPBA (77 wt.%, 2.7 mg, 0.012 mmol, 1.0 equiv.) in DMF (0.1 mL) and the mixture was stirred at 0 °C for 1 h, after which the reaction was quenched with saturated aqueous sodium thiosulfate and 3M aqueous HCl. The mixture was extracted with $CHCl_3$ (3 x 1 mL), dried over Na_2SO_4 and concentrated *in vacuo*. Purification by preparative TLC (3% MeOH, 2% HCO_2H in $CHCl_3$) afforded the title compound (2.1 mg, 0.062 mmol, 52% yield).

This material obtained contained an impurity (<10%) which we believe to be a mono-acetate of the title compound. This impurity could be removed further preparative TLC purification, but this in turn led to decomposition/loss of the title compound, resulting in poorer quality NMR data. The NMR spectra of both samples are attached.

^{1}H NMR (600 MHz, MeOD) **δ** 7.19 (d, *J* = 11.0 Hz, 1H), 6.35 (dd, *J* = 15.0, 11.0 Hz, 1H), 6.23 (dd, J = 15.0, 10.5 Hz, 1H), 3.60 (t, *J* = 2.5 Hz, 1H), 3.47 (dd, *J* = 7.0, 2.5 Hz 1H), 2.58 (t, *J* = 9.5 Hz, 1H), 2.47 (ddd, *J* = 10.5, 6.5, 5.0 Hz, 1H), 1.90 (s, 3H), 1.77 (td, J = 7.0, 5.0 Hz, 1H), 1.66 (tt, *J* = 12.0, 3.0 Hz, 1H), 1.51 (dt, *J* = 13.0, 3.5 Hz, 1H), 1.46 – 1.41 (m, 1H), 1.37 (td, *J* = 11.0, 7.0 Hz, 1H), 1.32 – 1.27 (m, 1H), 1.25 – 1.19 (m, 1H), 1.07 (d, *J* = 6.0 Hz, 3H), 1.03 (d, *J* = 6.5 Hz, 3H), 1.07 (d, *J* = 7.0 Hz, 3H).

^{13}C NMR (151 MHz, MeOD) **δ** 172.1, 149.1, 140.3, 126.5, 126.1, 82.6, 78.2, 77.7, 49.0, 46.4, 42.6, 41.1, 40.5, 40.2, 37.2, 19.7, 17.9, 15.5, 12.6.

$[\alpha]_D^{20}$ = -4.5 (c = 0.13, MeOH). **Lit $[\alpha]_D^{25}$** = -8 (c = 0.05, MeOH).[10]

HRMS (ESI+): Found [M+H]+ = 339.2157; $C_{19}H_{31}O_5$ requires 339.2166, Δ -2.66 ppm.

IR (film) vmax/cm^{-1}: 3421 (broad), 2920, 1683, 1640, 1266, 1034.

The spectroscopic data matched that previously reported in the literature (see Table 5).[10]

3.ii Procedures for the synthesis of small fragments.

(*Z*)-But-2-enoic acid **S1**

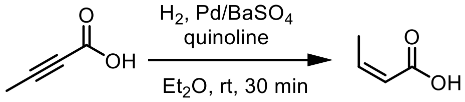

A 150 mL RBF was charged with 2-butynoic acid (2.00 g, 23.8 mmol, 1.00 equiv.) in dry Et_2O (80 mL). The mixture was degassed with N_2 and Rosenmund catalyst (5% Pd/$BaSO_4$, 500 mg) was added followed by quinoline (0.50 mL, 4.2 mmol, 0.18 equiv.). H_2 gas was bubbled through the mixture for 30 minutes, after which the mixture was filtered through a plug of silica and concentrated *in vacuo*. Purification by FCC (10% EtOAc/Pentane) afforded the title compound as a yellow oil (1.21 g, 14.1 mmol, 59% yield).

^{1}H NMR (400 MHz, $CDCl_3$) **δ** 11.8 (br s, 1H), 6.47 (dq, *J* = 11.5, 7.5 Hz, 1H), 5.83 (dd, *J* = 11.5, 2.0 Hz, 1H), 2.16 (dd, *J* = 7.5, 2.0 Hz, 3H).

^{13}C NMR (101 MHz, $CDCl_3$) **δ** 172.2, 148.1, 120.3, 15.8.

The spectroscopic data matched that previously reported in the literature.[11]

(*R*,*Z*)-4-Benzyl-3-(but-2-enoyl)oxazolidin-2-one **(*R*)-16**

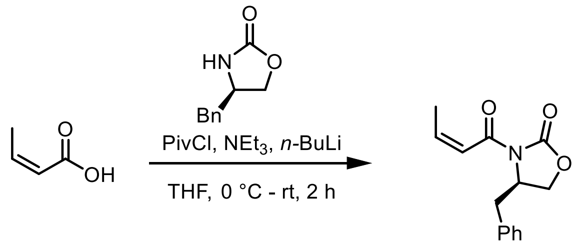

A 150 mL RBF was charged with (*Z*)-but-2-enoic acid **S1** (1.00 g,11.6 mmol, 1.00 equiv.) in dry THF (50 mL) at 0 °C. Triethylamine (2.4 mL, 17 mmol, 1.5 equiv.) was added followed by pivaloyl chloride (1.7 mL, 14 mmol, 1.2 equiv.) and the mixture was stirred at 0 °C for 45 minutes. Meanwhile, a separate 100 mL RBF was charged with (*R*)-4-benzyloxazolidin-2-one (2.06 g, 11.6 mmol, 1.00 equiv.) in dry THF (25 mL) at 0 °C. *N*-BuLi (2.5 M in hexanes, 4.6 mL, 12 mmol, 1.0 equiv.) was added dropwise and the mixture was stirred for 15 minutes at 0 °C. The oxazolidinone-containing mixture was then transferred *via* syringe into the acid-containing mixture at 0 °C. This was allowed to warm to rt and stirred for 2 h, after which the reaction was quenched with saturated aqueous ammonium chloride (20 mL). The mixture was then extracted with Et_2O (100 mL x 3), dried over Na_2SO_4 and concentrated *in vacuo*. Purification by MPLC (PuriFlash®, 0-20% EtOAc/Pentane) afforded the title compound as a colourless solid (1.90 g, 7.75 mmol, 67% yield). The (*S*)-enantiomer was prepared by the same method. Spectroscopic data were identical for both enantiomers.

^{1}H NMR (400 MHz, $CDCl_3$) **δ** 7.40 – 7.20 (m, 5H), 7.11 – 7.02 (m, 1H), 6.59 – 6.46 (m, 1H), 4.80 – 4.69 (m, 1H), 4.26 – 4.12 (m, 2H), 3.37 (dd, *J* = 13.5, 3.5 Hz, 1H), 2.82 (dd, *J* = 13.5, 9.5 Hz, 1H), 2.22 (dd, *J* = 7.0, 2.0 Hz, 3H).

^{13}C NMR (101 MHz, $CDCl_3$) δ 165.0, 153.4, 146.8, 135.5, 129.5, 129.0, 127.4, 120.2, 66.1, 55.2, 38.0, 16.3.

(*S*) $[\alpha]_D^{20}$ = +62.7 (c = 1.0, $CHCl_3$).

(*R*) $[\alpha]_D^{20}$ = -67.0 (c = 1.0, $CHCl_3$).

m.p. = 55-56 °C

HRMS (ESI+): Found [M+H]+ = 246.1115; $C_{14}H_{16}NO_3$ requires 246.1125, Δ -3.96 ppm.

IR (film) vmax/cm^{-1}: 1779, 1683, 1390, 1352, 1213, 704.

(R,Z)-4-Benzyl-3-(but-2-enoyl)-5,5-dimethyloxazolidin-2-one **(*R*)-S2**

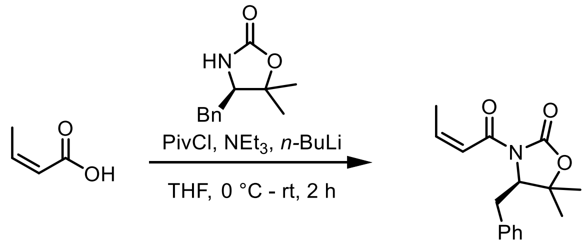

A 50 mL RBF was charged with (*Z*)-but-2-enoic acid **S1** (172 mg, 2.00 mmol, 1.00 equiv.) in dry THF (10 mL, 0.20 M at 0 °C. Triethylamine (0.42 mL, 3.0 mmol, 1.5 equiv.) was added followed by pivaloyl chloride (0.30 mL, 2.4 mmol, 1.2 equiv.) and the mixture was stirred at 0 °C for 45 minutes. Meanwhile, a separate 25 mL RBF was charged with (*R*)-4-benzyl-5,5-dimethyloxazolidin-2-one (411 mg, 2.00 mmol, 1.00 equiv.) in dry THF (5 mL) at 0 °C. *N*-BuLi (2.5 M in hexanes, 0.80 mL, 2.0 mmol, 1.0 equiv.) was added dropwise and the mixture was stirred for 15 minutes at 0 °C. The oxazolidinone-containing mixture was then transferred *via* syringe into the acid-containing mixture via syringe at 0 °C. This was allowed to warm to rt and stirred for 2 h, after which the reaction was quenched with saturated aqueous ammonium chloride (5 mL). The mixture was then extracted with Et_2O (20 mL x 3), dried over Na_2SO_4 and concentrated *in vacuo*. Purification by MPLC (PuriFlash®, 0-20% EtOAc/Pentane) afforded the title compound as a colourless solid (323 mg, 1.18 mmol, 59% yield).

^{1}H NMR (400 MHz, $CDCl_3$) **δ** 7.32 – 7.15 (m, 5H), 7.00 (dq, *J* = 11.5, 2.0 Hz, 1H), 6.42 (dq, *J* = 11.5, 7.0 Hz, 1H), 4.53 (dd, *J* = 9.5, 4.0 Hz, 1H), 3.20 (dd, *J* = 14.5, 4.0 Hz, 1H), 2.85 (dd, *J* = 14.5, 9.5 Hz, 1H), 2.08 (dd, *J* = 7.5, 2.0 Hz, 3H), 1.37 – 1.29 (m, 6H).

^{13}C NMR (101 MHz, $CDCl_3$) **δ** 165.4, 152.7, 146.5, 137.2, 129.2, 128.8, 126.9, 120.6, 82.2, 63.6, 35.5, 28.7, 22.5, 16.3.

m.p. = 64-65 °C

$[\alpha]_D^{20}$ = +33.0 (c = 0.66, $CHCl_3$).

HRMS (ESI+): Found [M+H]+ = 274.1435; $C_{16}H_{20}NO_3$ requires 274.1438, Δ -1.00 ppm.

IR (film) vmax/cm^{-1}: 2982, 1775, 1683, 1355, 1275, 1237, 736.

Methyl (*E*)-4-(diethoxyphosphoryl)but-2-enoate **19**

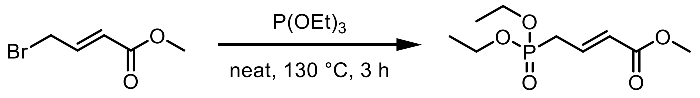

A 10 mL RBF fitted with a reflux condenser was charged with methyl 4-bromocrotonate (1.00 g, 5.59 mmol, 1.00 equiv.) and triethyl phosphite (0.96 mL, 5.6 mmol, 1.0 equiv.). The mixture was stirred at 130 °C for 3 h and then cooled to rt. Purification by FCC ($CHCl_3$) afforded the title compound as a colourless oil (1.07 g, 4.53 mmol, 81% yield).

^{1}H NMR (400 MHz, $CDCl_3$) **δ** 6.95 – 6.81 (m, 1H, H_4), 6.01 – 5.91 (m, 1H, H_3), 4.20 – 4.03 (m, 4H, H_6 x 4), 3.73 (s, 3H, H_1 x 3), 2.74 (ddt, *J* = 23.0, 8.0, 1.5 Hz, 2H, H_5 x 2), 1.42 – 1.26 (m, 6H, H_7 x 6).

^{13}C NMR (101 MHz, $CDCl_3$) **δ** 166.2 (d, *J* = 3.0 Hz, C_2), 138.0 (d, *J* = 11.0 Hz, C_4), 125.5 (d, *J* = 14.0 Hz, C_3), 62.42 (d, *J* = 6.5 Hz, C_6 x 2), 51.8 (C_1), 30.8 (d, *J* = 138.5 Hz, C_5), 16.5 (d, *J* = 6.0 Hz, C_7 x 2).

^{31}P NMR (162 MHz, $CDCl_3$) **δ** 24.29.

The spectroscopic data matched that previously reported in the literature.[6]

(*E*)-4-(*tert*-Butoxy)-3-methyl-4-oxobut-2-enoic acid **S3**

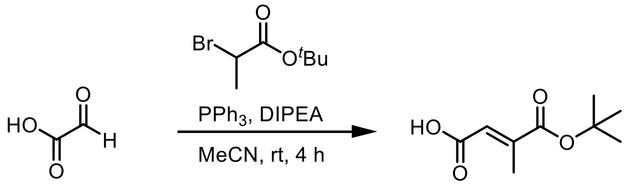

A 250 mL RBF was charged with *tert*-butyl 2-bromopropanoate (3.7 mL, 22 mmol, 1.1 equiv.) in MeCN (40 mL). Triphenylphosphine (5.25 g, 20.0 mmol, 1.00 equiv.) was added and the mixture was stirred at 65 °C for 16 h. The mixture was cooled to 0 °C and Glyoxylic acid hydrate (1.84 g, 20.0 mmol, 1.00 equiv.) and DIPEA (3.5 mL, 20 mmol, 1.0 equiv.) in MeCN (40 mL) were added. The mixture was warmed to rt and stirred for 4 h. The reaction mixture was transferred to a separatory funnel and saturated aqueous sodium bicarbonate (100 mL) was added followed by EtOAc (100 mL). The layers were separated and the aqueous phase was acidified using 3 M aqueous HCl. The acidified aqueous layer was extracted with EtOAc (3 x 100 mL) and the combined organic layers were dried over Na_2SO_4 and concentrated *in vacuo* to afford the title compound as a white solid (2.32 g, 12.5 mmol, 62% yield).

^{1}H NMR (400 MHz, $CDCl_3$) **δ** 6.71 (q, *J* = 1.5 Hz, 1H), 2.27 (d, *J* = 1.5 Hz, 3H), 1.52 (s, 9H).

^{13}C NMR (101 MHz, $CDCl_3$) **δ** 171.2, 166.1, 148.1, 124.9, 82.3, 28.1, 14.8.

The spectroscopic data matched that previously reported in the literature.[12]

tert-Butyl (*E*)-4-hydroxy-2-methylbut-2-enoate **S4**

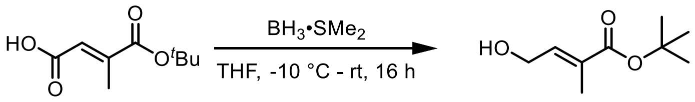

A 100 mL RBF was charged with carboxylic acid **S3** (2.32 g, 12.5 mmol, 1.00 equiv.) in dry THF (25 mL) at -10 °C. $BH_3.SMe_2$ (1.2 mL, 13 mmol, 1.0 equiv.) was added and the mixture was allowed to warm to rt overnight. The mixture was cooled to 0 °C and methanol (10 mL) was added and the mixture was concentrated *in vacuo*. This was repeated twice. Purification by MPLC (Puriflash®, 5-30% EtOAc/Pentane) afforded the title compound as a colourless oil (1.04 g, 6.02 mmol, 48% yield).

^{1}H NMR (400 MHz, $CDCl_3$) **δ** 6.77 – 6.68 (m, 1H), 4.34 (t, *J* = 6.0 Hz, 2H), 1.81 – 1.78 (m, 3H), 1.49 (s, 9H).

^{13}C NMR (101 MHz, $CDCl_3$) **δ** 167.1, 138.8, 129.0, 80.7, 60.0, 28.2, 12.8.

The spectroscopic data matched that previously reported in the literature.[12]

tert-Butyl (*E*)-4-bromo-2-methylbut-2-enoate **S5**

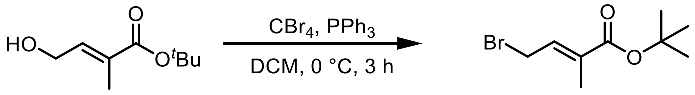

A 100 mL RBF was charged with alcohol **S4** (1.00 g, 5.81 mmol, 1.00 equiv.) in DCM (10 mL) at 0 °C. Tetrabromomethane (2.12 g, 6.39 mmol, 1.10 equiv.) in DCM (5 mL) was added followed by triphenylphosphine (1.60 g, 6.10 mmol, 1.05 equiv.) in DCM (5 mL) and the mixture was stirred at 0 °C for 3 h. The mixture was then filtered through a plug of silica and concentrated *in vacuo*. Purification by FCC (0-2% Et_2O/Pentane) afforded the title compound as a colourless oil (1.07 g, 4.57 mmol, 79% yield).

^{1}H NMR (400 MHz, $CDCl_3$) **δ** 6.82 (tq, *J* = 8.5, 1.5 Hz, 1H), 4.02 (d, *J* = 8.5 Hz, 2H), 1.87 (d, *J* = 1.5 Hz, 3H,), 1.49 (s, 9H).

^{13}C NMR (101 MHz, $CDCl_3$) **δ** 166.5, 134.0, 133.9, 81.1, 28.2, 26.5, 12.3.

The spectroscopic data matched that previously reported in the literature.[12]

tert-Butyl (*E*)-4-(diethoxyphosphoryl)-2-methylbut-2-enoate **22**

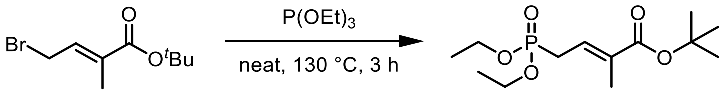

A 10 mL RBF fitted with a reflux condenser was charged with alkyl bromide **S5** (1.00 g, 4.27 mmol, 1.00 equiv.) and triethyl phosphite (0.66 mL, 3.9 mmol, 0.9 equiv.). The mixture was stirred at 130 °C for 3 h and then cooled to rt. Purification by MPLC (Puriflash®, 30-60% EtOAc/Pentane) afforded the title compound as a colourless oil (790 mg, 2.70 mmol, 70% yield).

^{1}H NMR (400 MHz, $CDCl_3$) **δ** 6.69 – 6.58 (m, 1H), 4.20 – 4.02 (m, 4H), 2.71 (dd, *J* = 23.5, 8.5 Hz, 2H), 1.84 (d, *J* = 4.5 Hz, 3H), 1.47 (s, 9H), 1.34 – 1.29 (m, 6H).

^{13}C NMR (101 MHz, $CDCl_3$) **δ** 166.7 (d, *J* = 3.0 Hz), 133.4 (d, *J* = 13.5 Hz), 129.1 (d, *J* = 11.0 Hz), 80.6, 62.3 (d, *J* = 6.5 Hz), 28.2, 27.7 (d, *J* = 139.0 Hz), 16.6 (d, *J* = 6.0 Hz), 12.7 (d, *J* = 2.5 Hz).

^{31}P NMR (162 MHz, $CDCl_3$) **δ** 25.80.

HRMS (ESI+): Found [M+H]+ = 293.1503; $C_{13}H_{26}O_5P$ requires 293.1512, Δ -3.20 ppm.

IR (film) vmax/cm^{-1}: 2981, 1706, 1253, 1159, 1050, 1026, 966.

3.iii Additional procedures for the synthesis of side-products and derivatives.

1-((3*S*,4*S*,5*S*)-4-(Dimethyl(phenyl)silyl)-3,5-dimethylcyclohex-1-en-1-yl)ethan-1-one **13**

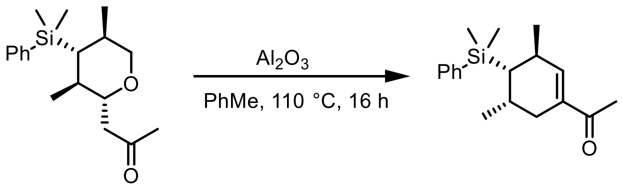

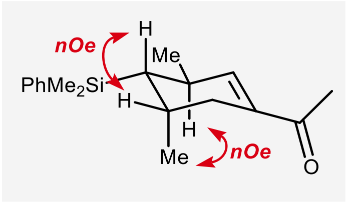

A 2-5 mL microwave vial was charged with tetrahydropyran **11** (60.9 mg, 0.200 mmol, 1.00 equiv.) and alumina (102 mg, 1.00 mmol, 5.00 equiv.) in dry PhMe (1.0 mL) and the mixture was heated at 110 °C for 16 h. Upon cooling, the mixture was filtered through a plug of silica, flushing with Et_2O and concentrated *in vacuo*. Quantitative ^{1}H NMR of the crude reaction mixture gave a 45% NMR yield with a 4:1 ratio of **13:12**. Purification by FCC (5% Et_2O/Pentane) afforded the title compound as a colourless oil (mixture of diastereomers, 8:1 ratio of **13:12**, 22.1 mg, 0.00771 mmol, 39% yield).

^{1}H NMR (400 MHz, $CDCl_3$) **δ** 7.56 – 7.46 (m, 2H), 7.38 – 7.28 (m, 3H), 6.69 – 6.63 (m, 1H), 2.56 – 2.43 (m, 1H), 2.25 (s, 3H), 2.24 – 2.13 (m, 2H), 2.13 – 2.02 (m, 1H), 1.04 (d, *J* = 7.0 Hz, 3H), 1.02 – 0.92 (m, 1H), 0.89 (d, *J* = 7.0 Hz, 3H), 0.41 – 0.31 (m, 6H).

^{13}C NMR (101 MHz, $CDCl_3$) **δ** 200.0, 146.6, 139.8, 137.5, 133.9, 128.9, 127.9, 33.2, 31.7, 30.6, 27.9, 25.4, 21.7, 18.1, -1.7, -2.3.

$[\alpha]_D^{20}$ = -17.2 (c = 1.0 $CHCl_3$).

HRMS (ESI+): Found [M+Na]+ = 309.1644; $C_{18}H_{26}OSiNa$ requires 309.1645, Δ -0.38 ppm.

IR (film) vmax/cm^{-1}: 2959, 1668, 1454, 1254, 1111, 835, 702.

(*E*)-1-(1-((3*S*,4*S*,5*R*)-4-(Dimethyl(phenyl)silyl)-3,5-dimethylcyclohex-1-en-1-yl)ethylidene)-2-(2,4-dinitrophenyl)hydrazine **14**

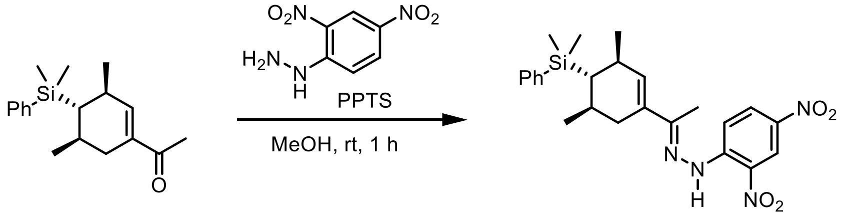

A 2 mL vial was charged with cyclohexene **12** (28.7 mg, 0.100 mmol, 1.00 equiv.) in MeOH (0.5 mL). 2,4-DNPH (60 wt.%, 33.0 mg, 0.100 mmol, 1.00 equiv.) was added followed by PPTS (2.5 mg, 0.010 mmol, 0.10 equiv.) and the mixture was stirred at rt for 1 h. The rection was quenched with water and the mixture was extracted with DCM (3 x 1 mL). The combined organic layers were dried over Na_2SO_4 and concentrated *in vacuo*. Purification by FCC (30-50% DCM/Pentane) afforded the title compound as a red solid (40.3 mg, 0.0864 mmol, 86% yield).

^{1}H NMR (400 MHz, $CDCl_3$) **δ** 11.22 (s, 1H), 9.13 (d, *J* = 2.5 Hz, 1H), 8.31 (dd, *J* = 9.5, 2.5 Hz, 1H), 8.01 – 7.91 (m, 1H), 7.60 – 7.49 (m, 2H), 7.41 – 7.31 (m, 3H), 6.28 – 6.23 (m, 1H), 2.60 – 2.47 (m, 2H), 2.19 – 2.05 (m, 4H), 1.98 – 1.87 (m, 1H), 1.09 – 0.99 (m, 6H), 0.77 (dd, *J* = 7.5, 5.5 Hz, 1H), 0.42 – 0.29 (m, 6H).

^{13}C NMR (101 MHz, $CDCl_3$) **δ** 154.1, 145.2, 139.5, 138.4, 138.0, 134.8, 133.9, 130.1, 129.5, 129.0, 127.9, 123.7, 116.8, 34.7, 32.1, 32.0, 29.7, 24.3, 24.1, 11.8, -2.6, -3.0.

m.p. = 125-126 °C.

$[\alpha]_D^{20}$ = -7.5 (c = 0.67, $CHCl_3$).

HRMS (ESI+): Found [M+H]+ = 467.2100; $C_{24}H_{31}N_4O_4Si$ requires 467.2109, Δ -1.96 ppm.

IR (film) vmax/cm^{-1}: 3315, 2956, 1618, 1427, 1335, 833, 741.

The structure of this compound was proven by X-ray crystallography (see section 6).

(*S*)-4-Benzyl-3-((1*R*,2*R*,6*R*,7*S*,8*S*,8a*S*)-4-((*tert*-butyldimethylsilyl)oxy)-7-(dimethyl(phenyl)silyl)-2,6,8-trimethyl-1,2,3,5,6,7,8,8a-octahydronaphthalene-1-carbonyl)oxazolidin-2-one ***cis-endo*-17** and (*S*)-4-Benzyl-3-((1*R*,2*S*,6*R*,7*S*,8*S*,8a*S*)-4-((*tert*-butyldimethylsilyl)oxy)-7-(dimethyl(phenyl)silyl)-2,6,8-trimethyl-1,2,3,5,6,7,8,8a-octahydronaphthalene-1-carbonyl)oxazolidin-2-one ***trans-endo*-17**

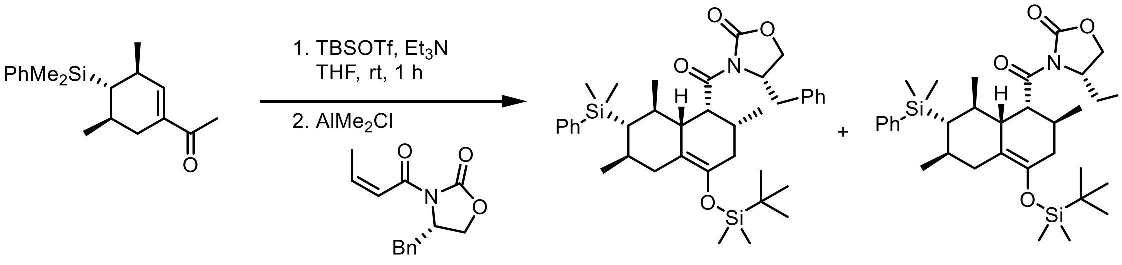

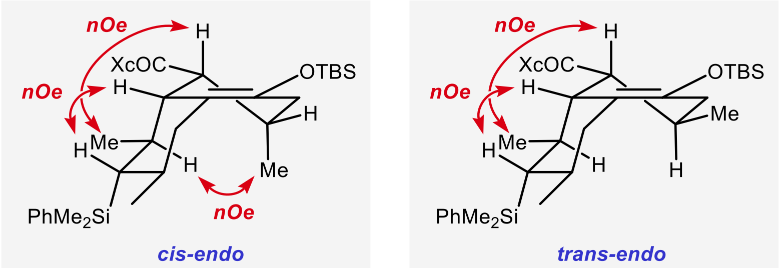

A 5 mL RBF was charged with cyclohexene **12** (28.7 mg, 0.100 mmol, 1.00 equiv.) in dry THF (0.5 mL). Triethylamine (0.035 mL, 0.25 mmol, 2.5 equiv.) was added followed by *tert*-butyldimethylsilyl trifluoromethanesulfonate (0.030 mL, 0.13 mmol, 1.3 equiv.) and the mixture was stirred for 1 h at rt. After this time, the mixture was diluted with pentane (10 mL) and washed with saturated aqueous sodium bicarbonate (5 mL) and water (5 mL). The combined organic layers were dried over Na_2SO_4 and concentrated *in vacuo*.

The crude silyl enol ether and oxazolidinone **(*S*)-16** (29.4 mg, 0.120 mmol, 1.20 equiv.) were dissolved in dry DCM (0.5 mL) and cooled to 0 °C. $AlMe_2Cl$ (0.9 M in heptane, 0.22 mL, 2.0 equiv.) was added and the mixture was stirred at 0 °C for 30 min. Pyridine (0.1 mL) was then added and the mixture was filtered through a plug of silica, flushing with Et_2O (10 mL), and concentrated *in vacuo*. Purification by FCC (6-8% Et_2O/Pentane) afforded ***cis-endo*-17** as a colourless oil (21.8 mg, 0.0337 mmol, 34% yield), and **trans-endo-17** as a colourless oil (11.9 mg, 0.0184 mmol, 18% yield).

Data for (*S*)-4-Benzyl-3-((1*R*,2*R*,6*R*,7*S*,8*S*,8a*S*)-4-((*tert*-butyldimethylsilyl)oxy)-7-(dimethyl(phenyl)silyl)-2,6,8-trimethyl-1,2,3,5,6,7,8,8a-octahydronaphthalene-1-carbonyl)oxazolidin-2-one ***cis-endo*-17**

^{1}H NMR (600 MHz, $CDCl_3$) **δ** 7.50 – 7.46 (m, 2H), 7.41 – 7.21 (m, 8H), 4.79 – 4.72 (m, 1H), 4.65 (t, *J* = 5.0 Hz, 1H), 4.14 – 4.05 (m, 2H), 3.32 (dd, *J* = 13.0, 3.5 Hz, 1H), 2.53 (dd, *J* = 13.0, 10.5 Hz, 1H), 2.48 – 2.41 (m, 1H), 2.35 – 2.29 (m, 1H), 2.19 – 2.10 (m, 1H), 2.05 – 1.98 (m, 2H), 1.86 – 1.78 (m, 2H), 1.66 – 1.56 (m, 1H), 1.00 (d, *J* = 7.0 Hz, 3H), 0.98 (s, 9H), 0.95 (d, *J* = 6.0 Hz, 3H), 0.91 (d, *J* = 6.0 Hz, 3H), 0.58 (dd, *J* = 10.5, 6.0 Hz, 1H), 0.36 – 0.28 (m, 6H), 0.22 – 0.14 (m, 6H).

^{13}C NMR (151 MHz, $CDCl_3$) **δ** 172.1, 153.5, 141.9, 140.4, 135.9, 133.8, 129.6, 129.1, 128.7, 127.8, 127.4, 113.7, 65.4, 55.7, 47.4, 42.2, 38.3, 38.2, 35.9, 32.63, 32.59, 32.4, 30.0, 26.1, 25.8, 22.3, 19.0, 18.4, -1.4, -1.7, -3.4, -3.9.

[α]$_D^{20}$ = +7.4 (c = 1.0, $CHCl_3$).

HRMS (ESI+): Found [M+Na]+ = 668.3537; $C_{38}H_{55}NO_4Si_2Na$ requires 668.3562, Δ -3.73 ppm.

IR (film) vmax/cm^{-1}: 2955, 1780, 1688, 1456, 1209, 837, 702.

Data for (*S*)-4-Benzyl-3-((1*R*,2*S*,6*R*,7*S*,8*S*,8a*S*)-4-((*tert*-butyldimethylsilyl)oxy)-7-(dimethyl(phenyl)silyl)-2,6,8-trimethyl-1,2,3,5,6,7,8,8a-octahydronaphthalene-1-carbonyl)oxazolidin-2-one ***trans-endo*-17**

^{1}H NMR (600 MHz, $CDCl_3$) **δ** 7.54 – 7.50 (m, 2H), 7.40 – 7.23 (m, 8H), 4.69 – 4.62 (m, 1H), 4.17 – 4.10 (m, 2H), 4.03 – 3.99 (m, 1H), 3.32 (dd, *J* = 13.0, 3.5 Hz, 1H), 2.60 (dd, *J* = 13.0, 10.0 Hz, 1H), 2.44 – 2.37 (m, 1H), 2.32 – 2.25 (m, 1H), 2.25 – 2.18 (m, 2H), 1.99 – 1.86 (m, 3H), 1.73 (d, *J* = 16.5 Hz, 1H), 1.15 (d, *J* = 7.0 Hz, 3H), 0.98 – 0.94 (m, 12H), 0.77 (d, *J* = 6.5 Hz, 3H), 0.56 (dd, *J* = 11.0, 5.5 Hz, 1H), 0.38 – 0.30 (m, 6H), 0.19 – 0.11 (m, 6H).

^{13}C NMR (151 MHz, $CDCl_3$) **δ** 172.8, 153.3, 140.4, 140.0, 135.8, 133.9, 129.6, 129.1, 128.7, 127.8, 127.4, 113.4, 65.8, 55.6, 43.6, 40.6, 38.2, 37.8, 33.8, 31.6, 31.0, 29.8, 29.5, 26.2, 26.1, 21.9, 20.4, 18.4, -1.7, -2.2, -3.5, -3.7.

[α]$_D^{20}$ = -7.4 (c = 0.70, $CHCl_3$).

HRMS (ESI+): Found [M+Na]+ = 668.3539; $C_{38}H_{55}NO_4Si_2Na$ requires 668.3562, Δ -3.43 ppm.

IR (film) vmax/cm^{-1}: 2956, 1780, 1699, 1456, 1208, 837, 702.

(*R*)-4-Benzyl-3-((1*S*,2*S*,6*R*,7*S*,8*S*,8a*S*)-4-((*tert*-butyldimethylsilyl)oxy)-7-(dimethyl(phenyl)silyl)-2,6,8-trimethyl-1,2,3,5,6,7,8,8a-octahydronaphthalene-1-carbonyl)-5,5-dimethyloxazolidin-2-one ***cis-exo*-S6** and (*R*)-4-Benzyl-3-((1*S*,2*R*,6*R*,7*S*,8*S*,8a*S*)-4-((*tert*-butyldimethylsilyl)oxy)-7-(dimethyl(phenyl)silyl)-2,6,8-trimethyl-1,2,3,5,6,7,8,8a-octahydronaphthalene-1-carbonyl)-5,5-dimethyloxazolidin-2-one ***trans-exo*-S6**

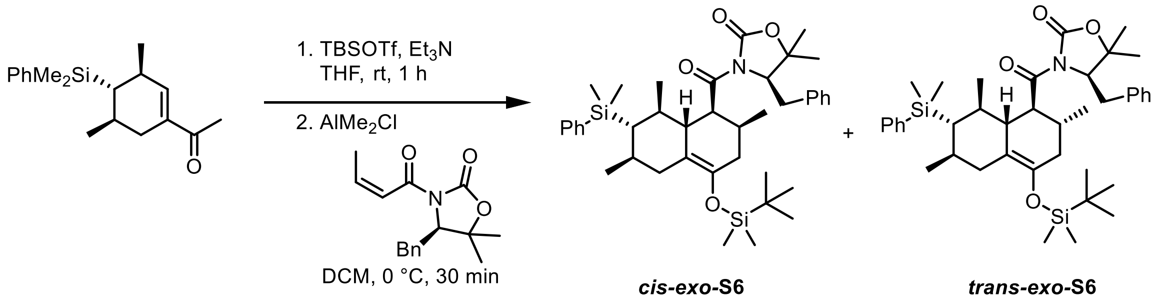

***cis-exo*-S6** ***trans-exo*-S6**

A 25 mL RBF was charged with cyclohexene **12** (200 mg, 0.698 mmol, 1.00 equiv.) in dry THF (3.5 mL). Triethylamine (0.25 mL, 1.8 mmol, 2.5 equiv.) was added followed by *tert*-butyldimethylsilyl trifluoromethanesulfonate (0.21 mL, 0.91 mmol, 1.3 equiv.) and the mixture was stirred for 1 h at rt. After this time, the mixture was diluted with pentane (50 mL) and washed with saturated aqueous sodium bicarbonate (25 mL) and water (25 mL). The combined organic layers were dried over Na_2SO_4 and concentrated *in vacuo*.

The crude silyl enol ether and oxazolidinone **(*R*)-S2** (229 mg, 0.838 mmol, 1.20 equiv.) were dissolved in dry DCM (3.5 mL) and cooled to 0 °C. $AlMe_2Cl$ (0.9 M in heptane, 1.1 mL, 1.4 equiv.) was added and the mixture was stirred at 0 °C for 30 min. Pyridine (0.5 mL) was then added and the mixture was filtered through a plug of silica, flushing with Et_2O (30 mL), and concentrated *in vacuo*. Purification by FCC (5-10% Et_2O/Pentane) afforded ***cis-exo*-S6** as a colourless oil (201 mg, 0.298 mmol, 43% yield), and ***trans-exo*-S6** as a white solid (173 mg, 0.257 mmol, 37% yield).

Data for (*R*)-4-Benzyl-3-((1*S*,2*S*,6*R*,7*S*,8*S*,8a*S*)-4-((*tert*-butyldimethylsilyl)oxy)-7-(dimethyl(phenyl)silyl)-2,6,8-trimethyl-1,2,3,5,6,7,8,8a-octahydronaphthalene-1-carbonyl)-5,5-dimethyloxazolidin-2-one ***cis-exo*-S6**

^{1}H NMR (400 MHz, $CDCl_3$) **δ** 7.54 – 7.44 (m, 2H), 7.35 – 7.16 (m, 8H), 4.51 (dd, *J* = 10.0, 3.5 Hz, 1H), 3.95 (dd, *J* = 5.5, 4.0 Hz, 1H), 3.05 (dd, *J* = 14.5, 3.5 Hz, 1H), 2.86 – 2.71 (m, 2H), 2.23 (dd, *J* = 16.0, 5.0 Hz, 1H), 2.15 – 2.03 (m, 2H), 1.94 (dd, *J* = 16.0, 6.0 Hz, 1H), 1.67 – 1.52 (m, 1H), 1.45 – 1.22 (m, 8H), 0.95 – 0.90 (m, 12H), 0.87 – 0.81 (m, 6H), 0.69 (t, *J* = 11.0 Hz, 1H), 0.34 – 0.26 (m, 6H), 0.10 (s, 6H).

^{13}C NMR (101 MHz, $CDCl_3$) **δ** 175.1, 152.7, 141.3, 139.1, 137.2, 133.8, 129.3, 128.8, 128.6, 127.8, 126.9, 115.7, 81.7, 63.8, 45.4, 44.7, 40.6, 38.9, 36.7, 36.5, 35.3, 33.5, 29.6, 28.6, 26.0, 24.9, 23.2, 22.4, 18.4, 16.9, -0.7, -0.8, -3.7, -3.9.

$[\alpha]_D^{20}$ = +35.9 (c = 0.69, $CHCl_3$).

HRMS (ESI+): Found [M+H]+ = 674.4059; $C_{40}H_{60}NO_4Si_2$ requires 674.4055, Δ 0.53 ppm.

IR (film) vmax/cm^{-1}: 2956, 1777, 1697, 1457, 1251, 838, 702.

Data for (*R*)-4-Benzyl-3-((1*S*,2*R*,6*R*,7*S*,8*S*,8a*S*)-4-((*tert*-butyldimethylsilyl)oxy)-7-(dimethyl(phenyl)silyl)-2,6,8-trimethyl-1,2,3,5,6,7,8,8a-octahydronaphthalene-1-carbonyl)-5,5-dimethyloxazolidin-2-one ***trans-exo*-S6**

^{1}H NMR (400 MHz, $CDCl_3$) **δ** 7.54 – 7.44 (m, 2H), 7.38 – 7.17 (m, 8H), 4.56 (dd, *J* = 11.0, 2.0 Hz, 1H), 3.72 (dd, *J* = 10.5, 8.5 Hz, 1H), 3.20 (dd, *J* = 14.5, 2.0 Hz, 1H), 2.79 – 2.65 (m, 2H), 2.47 (t, *J* = 9.0 Hz, 1H), 2.06 – 1.86 (m, 3H), 1.63 – 1.45 (m, 2H), 1.36 (s, 4H), 1.29 (s, 3H), 1.02 – 0.96 (m, 3H), 0.93 (s, 9H), 0.85 (d, *J* = 6.5 Hz, 6H), 0.64 (dd, *J* = 11.5, 10.0 Hz, 1H), 0.34 – 0.26 (m, 6H), 0.16 – 0.05 (m, 6H).

^{13}C NMR (101 MHz, $CDCl_3$) **δ** 178.4, 152.6, 141.1, 139.9, 137.6, 133.8, 129.1, 128.8, 128.6, 127.8, 126.8, 117.0, 81.7, 64.6, 50.2, 48.8, 41.0, 38.8, 38.5, 35.5, 35.1, 34.5, 32.8, 29.0, 25.9, 24.8, 23.2, 22.6, 19.0, 18.3, -0.8, -1.0, -3.6, -4.1.

m.p. = decomposed >200 °C.

$[\alpha]_D^{20}$ = +20.4 (c = 1.0, $CHCl_3$).

HRMS (ESI+): Found [M+Na]+ = 696.3876; $C_{40}H_{59}NO_4Si_2Na$ requires 696.3875, Δ 0.16 ppm.

IR (film) vmax/cm^{-1}: 2955, 1777, 1693, 1458, 1202, 838, 701.

The structure of this compound was proven by X-ray crystallography (see section 6).

Coprophilin

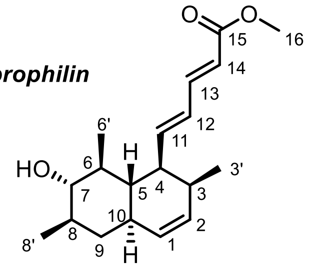

4. NMR tables of natural products

Table 1. Comparison of NMR data for Coprophilin

Isolation[7]	2018 synthesis[6]	This work		Assignment
7.29 (dd, *J* = 15.4, 10.5 Hz, 1H)	7.29 (dd, *J* = 15.5, 11.0 Hz, 1H)	7.29 (dd, *J* = 15.5, 11.0 Hz, 1H)		H13
6.18 (dd, *J* = 15.4, 10.3 Hz, 1H)	6.19 (dd, *J* = 14.5, 11.0 Hz, 1H)	6.19 (dd, *J* = 15.0, 10.5 Hz, 1H)		H11
6.10 (ddd, *J* = 15.4, 10.3, 9.0 Hz, 1H)	6.10 (dd, *J* = 14.5, 11.0 Hz, 1H)	6.10 (dd, *J* = 15.0, 11.0 Hz, 1H)		H12
5.80 (d, *J* = 15.4 Hz, 1H)	5.79 (d, *J* = 15.5 Hz, 1H)	5.79 (d, *J* = 15.5 Hz, 1H)		H14
5.57 (ddd, *J* = 9.4, 4.4, 2.7 Hz, 1H)	5.60–5.56 (m, 1H)	5.58 (ddd, *J* = 9.5, 4.5, 2.5 Hz, 1H)		H2
5.45 (dt, *J* = 9.4, 2.1Hz, 1H)	5.47–5.44 (m, 1H)	5.46 (dt, *J* = 9.5, 2.0 Hz, 1H)		H1
3.72 (s, 3H)	3.74 (s, 3H)	3.74 (s, 3H)		H16 x 3
2.7 (t, *J* = 9.6 Hz, 1H)	2.76–2.70 (m, 1H)	2.73 (t, *J* = 9.5 Hz, 1H)		H7
2.44 (ddd, *J* = 10.2, 9.0, 5.5 Hz, 1H)	2.45 (ddd, *J* = 11.0, 8.5, 4.5 Hz, 1H)	2.45 (ddd, *J* = 10.5, 9.0, 5.5 Hz, 1H)		H4
2.2 (m, 1H)	2.28–2.13 (m, 1H)	2.25 – 2.16 (m, 1H)		H3
1.85 (m, 1H)	1.92–1.82 (m, 1H)	1.90 – 1.82 (m, 1H)		H10
1.75 (dd, *J* = 13.3, 3.4 Hz, 1H)	1.76 (ddd, *J* = 13.5, 4.0, 3.5 Hz, 1H)	1.76 (dt, *J* = 13.0, 3.5 Hz, 1H)		H9a
1.45 (m, 1H)	1.60–1.38 (m, 1H)	1.53 – 1.43 (m, 1H)		H8
1.39 (m, 1H)	1.38–1.18 (m, 1H)	1.35 – 1.26 (m, 1H)		H6
1.07 (t, *J* = 9.8, 1H)	1.13–1.00 (m, 1H)	1.11 – 1.02 (m, 7H)		H5
1.06 (d, *J* = 6.2 Hz, 3H)	1.07 (d, *J* = 6.0 Hz, 3H)			H6' x 3
1.02 (d, *J* = 6.2 Hz, 3H)	1.04 (d, *J* = 6.0 Hz, 3H)			H8' x 3
0.94 (q, *J* = 12.2 Hz, 1 H)	1.00–0.88 (m, 1H)	1.00 – 0.93 (m, 4H)		H9b
0.94(d, *J* = 7.1 Hz, 3H)	0.95 (d, *J* = 7.0 Hz, 3H)			H3' x 3
			$\Delta\delta_C$[7]	
167.8	167.8	167.9	0.1	C15
149.7	149.6	149.8	0.1	C11
145.2	145.2	145.4	0.2	C13
132.7	132.7	132.8	0.1	C2
131.6	131.6	131.7	0.1	C1
126.8	126.8	127.0	0.2	C12
118.8	118.8	118.9	0.1	C14
82.1	82.1	82.3	0.2	C7
51.1	51.5	51.6	0.5	C16
49.4	49.4	49.6	0.2	C4
46.0	46.0	46.1	0.1	C5
43.9	43.9	44.0	0.1	C6
41.7	41.7	41.8	0.1	C10
39.6	39.6	39.7	0.1	C8
39.2	39.2	39.3	0.1	C9
36.3	36.3	36.5	0.2	C3
19.1	19.0	19.2	0.1	C8'
17.9	17.9	18.0	0.1	C6'
16.5	16.5	16.7	0.2	C3'

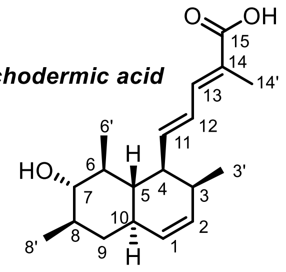

Trichodermic acid

Table 2. Comparison of NMR data for Trichodermic Acid

**2013 synthesis**[8]	**This work**		**Assignment**
7.30 (d, *J* = 11.0 Hz, 1H)	7.30 (dq, *J* = 10.0, 1.5 Hz, 1H)		H13
6.28 (dd, *J* = 11.0, 14.5 Hz, 1H)	6.28 (dd, *J* = 15.0, 11.0 Hz, 1H)		H12
6.20 (dd, *J* = 11.0, 14.5 Hz, 1H)	6.20 (dd, *J* = 15.0, 10.5 Hz, 1H)		H11
5.59 (ddd, *J* = 9.5, 4.5, 2.0 Hz, 1H)	5.59 (ddd, *J* = 9.5, 4.5, 3.0 Hz, 1H)		H2
5.50–5.40 (m, 1H)	5.46 (dt, *J* = 9.5, 2.0 Hz, 1H)		H1
2.74 (dd, *J* = 10.0, 9.5 Hz, 1H)	2.74 (t, *J* = 9.5 Hz, 1H)		H7
2.50 (ddd, *J* = 11.0, 9.5, 6.0 Hz, 1H)	2.50 (ddd, *J* = 10.5, 9.0, 5.5 Hz, 1H)		H4
2.28–2.13 (m, 1H)	2.22 (qt, *J* = 7.0, 5.0 Hz, 1H)		H3
1,93 (s, 1H)	1.94 (d, *J* = 1.5 Hz, 3H)		H14' x 3
1.88 (ddddd, *J* = 13.5, 10.0, 3.5, 2.5, 2.0 Hz, 1H)	1.88 (tq, *J* = 12.5, 2.5 Hz, 1H)		H10
1.76 (ddd, *J* = 13.5, 4.0, 3.5 Hz, 1H)	1.76 (dt, *J* = 13.0, 3.5 Hz, 1H)		H9a
1.55–1.41 (m, 1H)	1.55 – 1.44 (m, 1H)		H8
1.38–1.20 (m, 1H)	1.37 – 1.28 (m, 1H)		H6
1.15–1.00 (m, 1H)	1.12 – 1.06 (m, 4H)		H5
1.08 (d, *J* = 6.5 Hz, 3H)			H6' x 3
1.04 (d, *J* = 6.5 Hz, 3H)	1.04 (d, *J* = 6.5 Hz, 3H)		H8' x 3
1.00–0.90 (m, 1H)	1.01 – 0.92 (m, 4H)		H9b
0.96 (d, *J* = 7.5 Hz, 3H)			H3' x 3
		**$\Delta\delta_C$[8]**	
172.8	172.9	0.1	C15
149.3	149.5	0.2	C11
140.7	140.9	0.2	C13
132.7	132.9	0.2	C2
131.5	131.6	0.1	C1
124.4	124.6	0.2	C12
124.1	124.2	0.1	C14
82.2	82.3	0.1	C7
49.8	50.0	0.2	C4
45.9	46.0	0.1	C5
43.8	44.0	0.2	C6
41.8	42.0	0.2	C10
39.6	39.7	0.1	C8
39.2	39.4	0.2	C9
36.5	36.7	0.2	C3
19.0	19.2	0.2	C8'
18.0	18.2	0.2	C6'
16.5	16.7	0.2	C3'
12.3	12.5	0.2	C14'

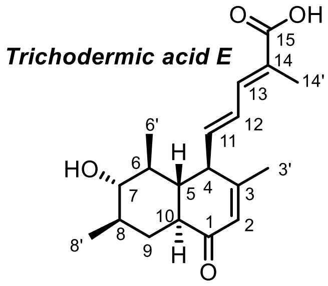

Table 3. Comparison of NMR data for Trichodermic Acid E

Isolation[9]	This work		Assignment
7.17 (d, *J* = 11.2 Hz, 1H)	7.20 (d, *J* = 11.5 Hz, 1H)		H13
6.63 (dd, *J* = 15.2, 11.2 Hz, 1H)	6.62 (dd, *J* = 15.0, 11.5 Hz, 1H)		H12
5.91 (m, 1H)	5.97 (dd, *J* = 15.0, 9.5 Hz, 1H)		H11
5.96 (s, 1H)	5.94 (dd, *J* = 2.0, 1.5 Hz, 1H)		H2
3.23 (t, *J* = 9.4 Hz, 1H)	3.21 (t, *J* = 8.5 Hz, 1H)		H4
2.63 (t, *J* = 9.6 Hz, 1H)	2.61 (t, *J* = 10.0 Hz, 1H)		H7
2.28 (m, 1H)	2.25 (td, *J* = 12.0, 3.5 Hz, 1H)		H10
2.20 (m, 1H)	2.18 (dt, *J* = 14.0, 3.5 Hz, 1H)		H9a
1.97 (s, 3H)	1.95 (d, *J* = 1.5 Hz, 3H)		H14' x 3
1.88 (s, 3H)	1.86 (t, *J* = 1.0 Hz, 3H)		H3' x 3
1.62 (m, 1H)	1.61 (ddd, *J* = 12.5, 10.5, 8.5 Hz, 1H)		H5
1.53 (m, 1H)	1.51 (tq, *J* = 10.0, 6.5 Hz, 1H)		H6
1.43 (m, 1H)	1.41 (dddd, *J* = 12.5, 10.0, 6.5, 3.5 Hz, 1H)		H8
1.14 (d, *J* = 6.4 Hz, 3H)	1.12 (d, *J* = 6.5 Hz, 3H)		H6' x 3
1.08 (d, *J* = 6.4 Hz, 3H)	1.06 (d, *J* = 6.5 Hz, 3H)		H8' x 3
0.92 (m, 1H)	0.94 (q, *J* = 12.5 Hz, 1H)		H9b
		**Δδ$_C$[9]**	
202.4	202.4	0.0	C1
174.0	172.0	-2.0*	C15
162.6	162.4	-0.2	C3
144.9	146.2	1.3*	C11
137.0	138.8	1.8*	C13
130.6	130.4	-0.2	C12
134.1	128.4	-5.7*	C14
126.9	127.0	0.1	C2
81.3	81.3	0.0	C7
52.2	52.2	0.0	C4
49.7	49.8	0.1	C5
49.4	49.6	0.2	C10
49.7	47.9	-1.8*	C6
39.4	39.4	0.0	C8
33.3	33.3	0.0	C9
23.6	23.6	0.0	C3'
19.6	19.6	0.0	C8'
18.9	18.9	0.0	C6'
13.2	12.9	-0.3	C14'

* Some ^{13}C shifts do not match those reported in the isolation paper. These peaks are not visible in the published 1D ^{13}C spectrum, and have presumably been picked out of the 2D HSQC or HMBC spectra in the isolation paper. We believe our data to be a more accurate representation of these chemical shift values.

Trichodermic acid G

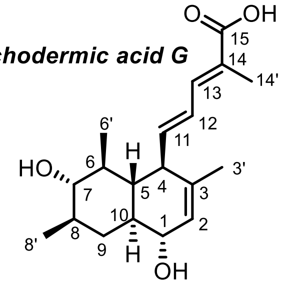

Table 4. Comparison of NMR data for Trichodermic Acid G

Isolation[9]	This work		Assignment
7.12 (d, J = 11.2 Hz, 1H)	7.15 (d, J = 11.5 Hz, 1H)		H13
6.45 (dd, J = 15.2, 11.2 Hz, 1H)	6.45 (dd, J = 15.0, 11.5 Hz, 1H)		H12
5.78 (dd, J = 15.2, 9.4 Hz, 1H)	5.79 (dd, J = 15.0, 9.5 Hz, 1H)		H11
5.53 (s, 1H)	5.53 (q, J = 1.5 Hz, 1H)		H2
3.66 (d, J = 7.4 Hz 1H)	3.69 – 3.62 (m, 1H)		H1
2.68 (m, 1H)	2.68 (t, J = 8.0 Hz, 1H)		H4
2.63 (t, J = 9.6 Hz, 1H)	2.64 (t, J = 10.0 Hz, 1H)		H7
2.22 (d, J = 13.8 Hz, 1H)	2.22 (dt, J = 13.0, 3.5 Hz, 1H)		H9a
1.91 (s, 3H)	1.91 (d, J = 1.5 Hz, 3H)		H14' x 3
1.61 (s, 3H)	1.60 (t, J = 2.0 Hz, 3H)		H3' x 3
1.37 (m, 1H)	1.42 – 1.27 (m, 2H)		H8
1.31 (m, 1H)			H6
1.14 (m, 1H)	1.19 – 1.09 (m, 2H)		H10
1.15 (m, 1H)			H5
1.07 (d, J = 6.4 Hz, 3H)	1.07 (d, J = 6.5 Hz, 3H)		H6' x 3
1.04 (d, J = 6.4 Hz, 3H)	1.03 (d, J = 6.5 Hz, 3H)		H8' x 3
0.78 (dd, J = 13.8, 12.0 Hz, 1H)	0.78 (td, J = 13.0, 11.0 Hz, 1H)		H9b
		$\Delta\delta_C$[9]	
172.5	172.2	-0.3	C15
148.8	149.0	0.2	C11
139.5	139.7	0.2	C13
135.6	135.5	-0.1	C3
130.7	130.7	0.0	C2
128.5	128.5	0.0	C12
127.2	126.8	-0.4	C14
82.1	82.1	0.0	C7
72.1	72.1	0.0	C1
51.7	51.7	0.0	C4
48.7	48.8	0.1	C5
48.1	48.1	0.0	C6
47.0	46.9	-0.1	C10
39.9	39.9	0.0	C8
37.4	37.4	0.0	C9
22.0	22.0	0.0	C3'
19.6	19.6	0.0	C8'
18.3	18.3	0.0	C6'
12.8	12.7	-0.1	C14'

Table 5. Comparison of NMR data for Trichodermic Acid D

Trichodermic acid D

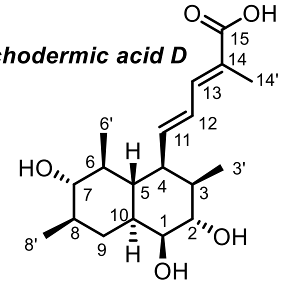

Isolation[10]	This work		Assignment
7.17 (d, *J* = 11.4 Hz, 1H)	7.19 (d, *J* = 11.0 Hz, 1H)		H13
6.36 (dd, *J* = 15.0, 11.4 Hz, 1H)	6.35 (dd, *J* = 15.0, 11.0 Hz, 1H)		H12
6.22 (dd, *J* = 15.0, 10.8 Hz, 1H)	6.23 (dd, *J* = 15.0, 10.5 Hz, 1H)		H11
3.63 – 3.57 (m, 1H)	3.60 (t, *J* = 2.5 Hz, 1H)		H1
3.47 (dd, *J* = 7.2, 1.2 Hz, 1H)	3.47 (dd, *J* = 7.0, 2.5 Hz 1H)		H2
2.57 (t, *J* = 9.6 Hz, 1H)	2.58 (t, *J* = 9.5 Hz, 1H)		H7
2.49 – 2.46 (m, 1H)	2.47 (ddd, *J* = 10.5, 6.5, 5.0 Hz, 1H)		H4
1.90 (s, 3H)	1.90 (s, 3H)		H14’ x 3
1.81 – 1.73 (m, 1H)	1.77 (td, *J* = 7.0, 5.0 Hz, 1H)		H3
1.71 – 1.61 (m, 1H)	1.66 (tt, *J* = 12.0, 3.0 Hz, 1H)		H10
1.49 (dt, *J* = 12.6, 3.0 Hz, 1H)	1.51 (dt, *J* = 13.0, 3.5 Hz, 1H)		H9a
1.47 – 1.40 (m, 1H)	1.46 – 1.40 (m, 1H)		H8
1.37 (td, *J* = 10.8, 6.6 Hz 1H)	1.37 (td, *J* = 11.0, 7.0 Hz, 1H)		H5
1.31 – 1.25 (m, 1H)	1.32 – 1.27 (m, 1H)		H9b
1.25 – 1.18 (m, 1H)	1.25 – 1.19 (m, 1H)		H6
1.07 (d, *J* = 6.0 Hz, 3H)	1.07 (d, *J* = 6.0 Hz, 3H)		H8' x 3
1.03 (d, *J* = 6.0 Hz, 3H)	1.03 (d, *J* = 6.5 Hz, 3H)		H6’ x 3
1.01 (d, *J* = 7.2 Hz, 3H)	1.01 (d, *J* = 7.0 Hz, 3H)		H3’ x 3
		**$\Delta\delta_C$[10]**	
172.4	172.1	-0.3	C15
149.0	149.1	0.1	C11
140.2	140.3	0.1	C13
126.5	126.5	0.0	C12
126.3	126.1	-0.2	C14
82.5	82.6	0.1	C7
78.2	78.2	0.0	C2
77.7	77.7	0.0	C1
48.8	49.0	0.2	C4
46.4	46.4	0.0	C6
42.6	42.6	0.0	C5
41.1	41.1	0.0	C10
40.4	40.5	0.1	C8
40.2	40.2	0.0	C3
37.1	37.2	0.1	C9
19.7	19.7	0.0	C6'
18.0	17.9	-0.1	C8'
15.5	15.5	0.0	C3'
12.7	12.6	-0.1	C14’

5. NMR spectra of novel compounds

5.i NMR spectra of synthetic intermediates and natural products.

(*R*)-5-Methyl-5,6-dihydro-2H-pyran-2-one 8

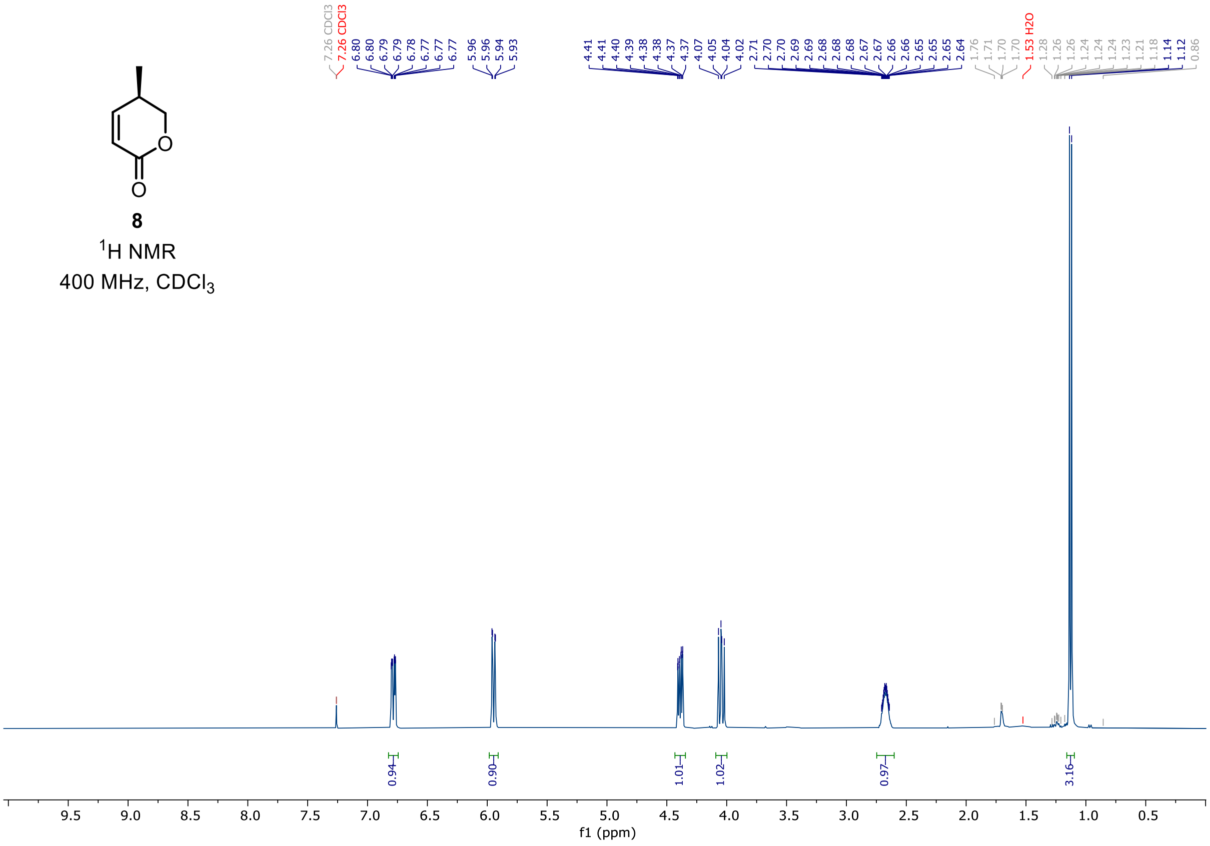

8
^{1}H NMR
400 MHz, $CDCl_3$

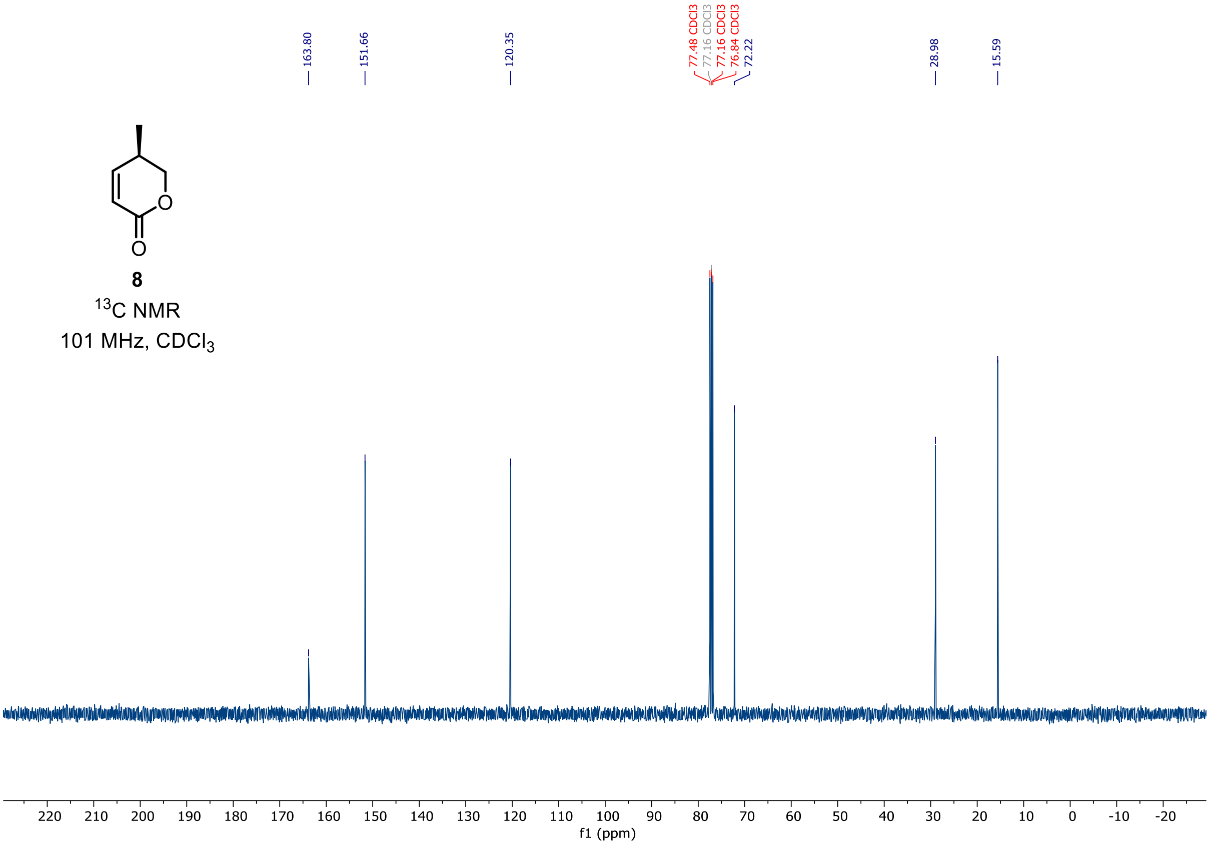

8
^{13}C NMR
101 MHz, $CDCl_3$

(3*R*,4*R*,5*S*)-4-(Dimethyl(phenyl)silyl)-3,5-dimethyltetrahydro-2H-pyran-2-one 9

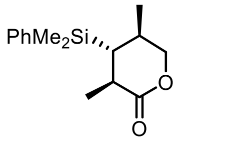

9

^{1}H NMR

400 MHz, $CDCl_3$

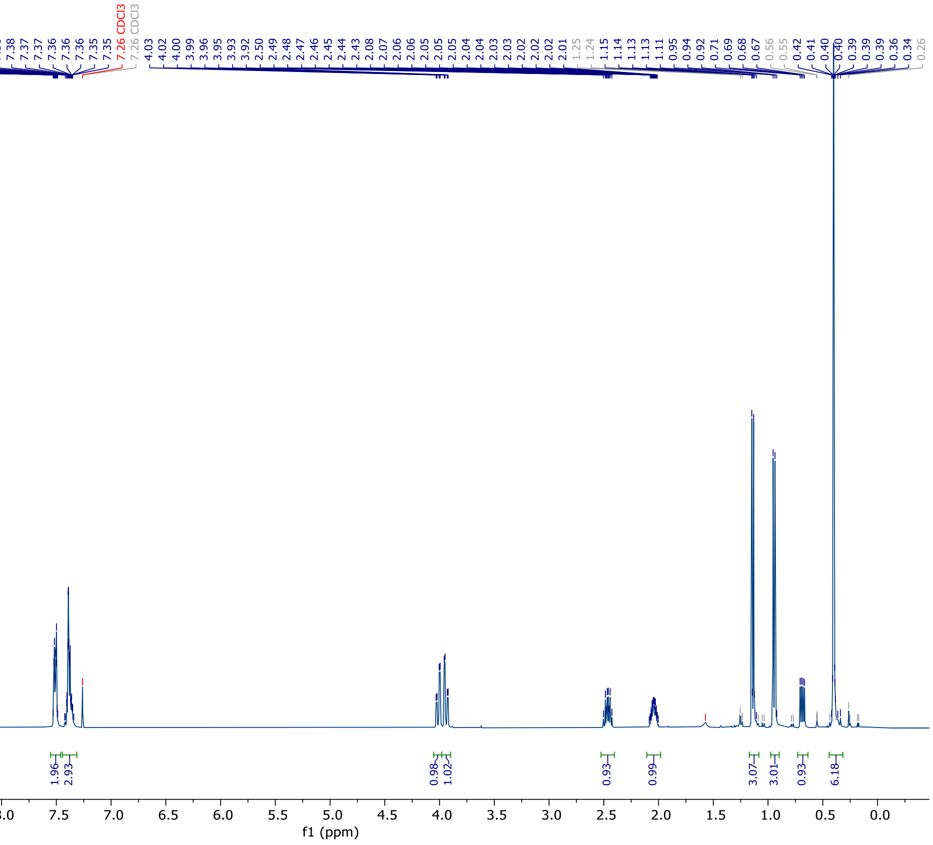

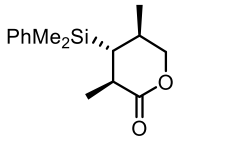

9

^{13}C NMR

101 MHz, $CDCl_3$

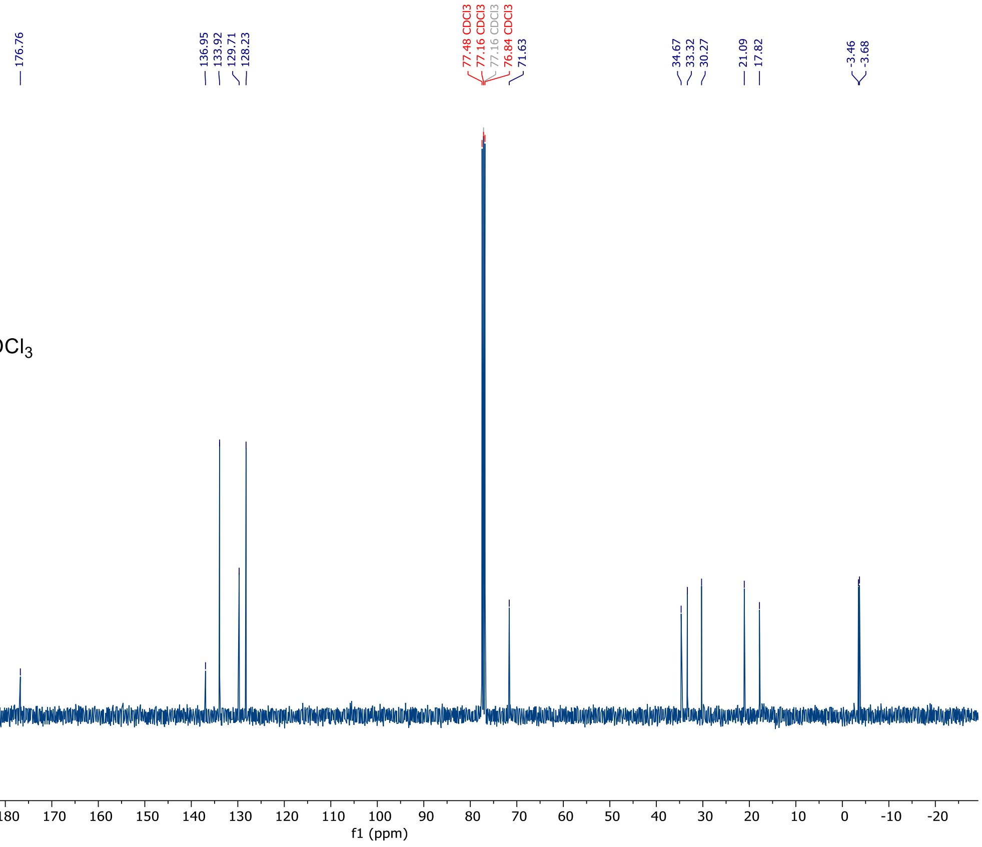

1-((2*R*,3*R*,4*R*,5*S*)-4-(Dimethyl(phenyl)silyl)-3,5-dimethyltetrahydro-2H-pyran-2-yl)propan-2-one 11

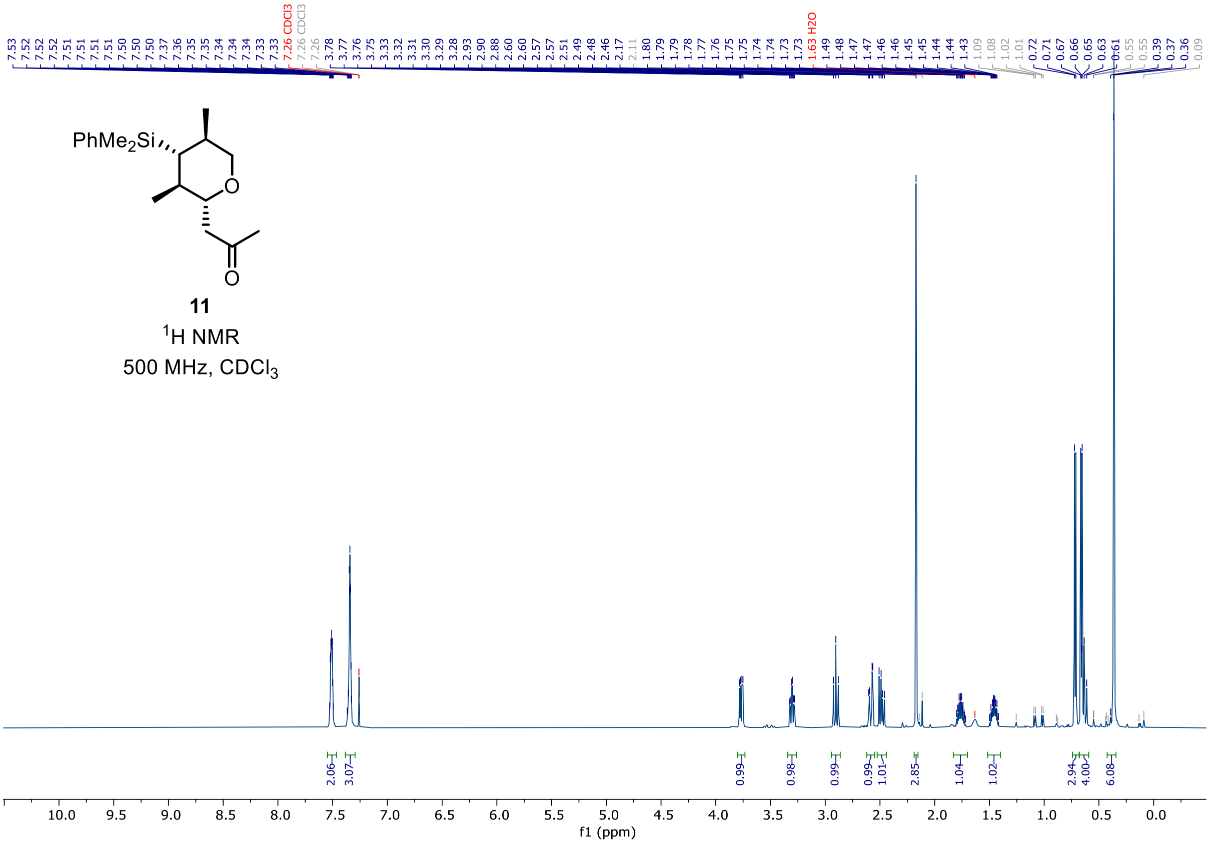

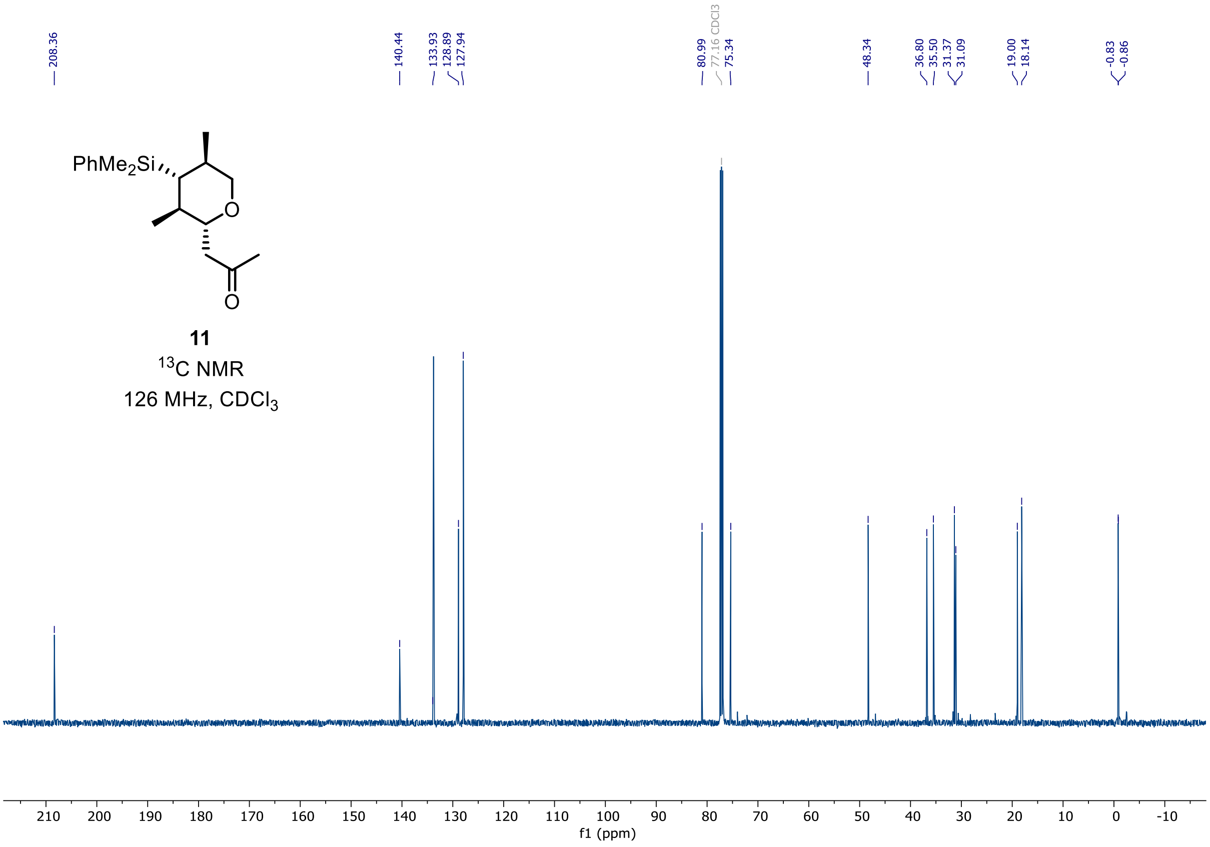

1-((3*S*,4*S*,5*R*)-4-(Dimethyl(phenyl)silyl)-3,5-dimethylcyclohex-1-en-1-yl)ethan-1-one 12

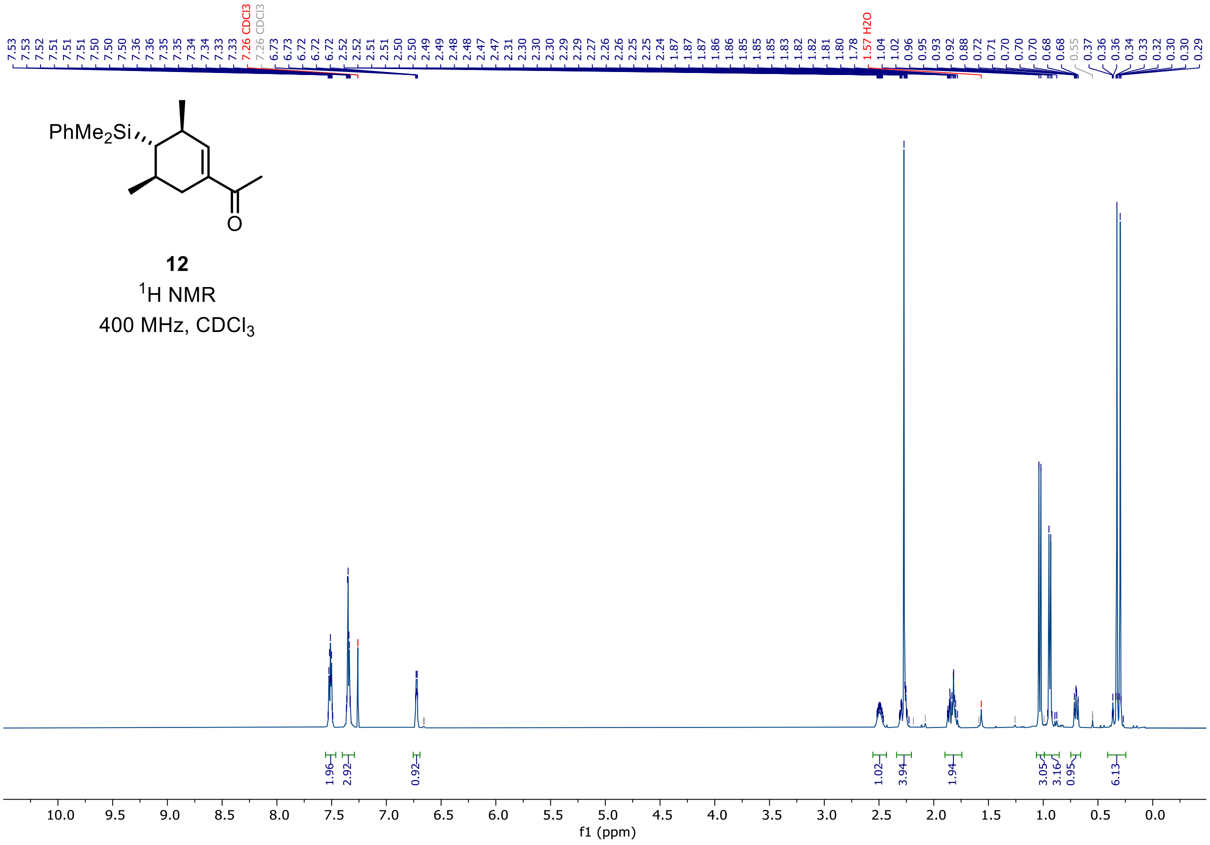

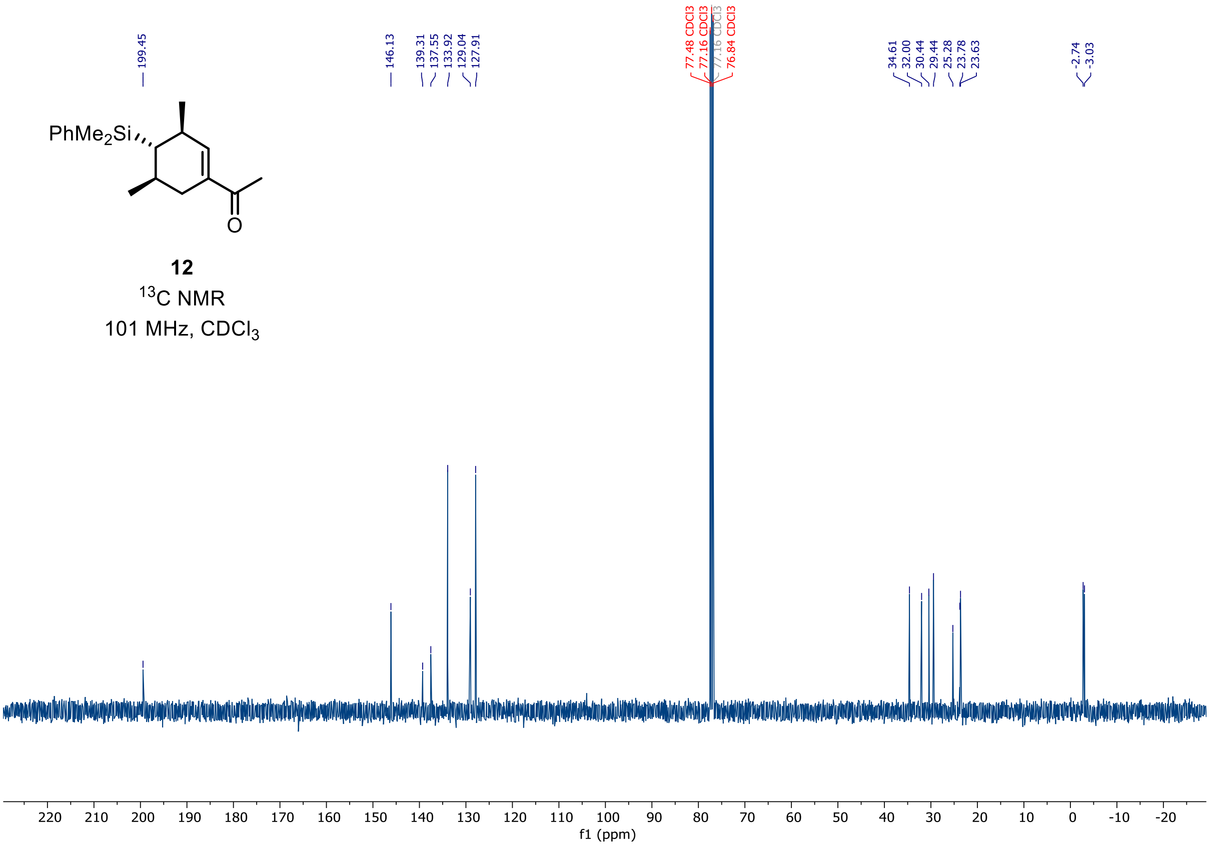

(*R*)-4-Benzyl-3-((1*S*,2*S*,6*R*,7*S*,8*S*,8a*S*)-4-((*tert*-butyldimethylsilyl)oxy)-7-(dimethyl(phenyl)silyl)-2,6,8-trimethyl-1,2,3,5,6,7,8,8a-octahydronaphthalene-1-carbonyl)oxazolidin-2-one *cis-exo*-17

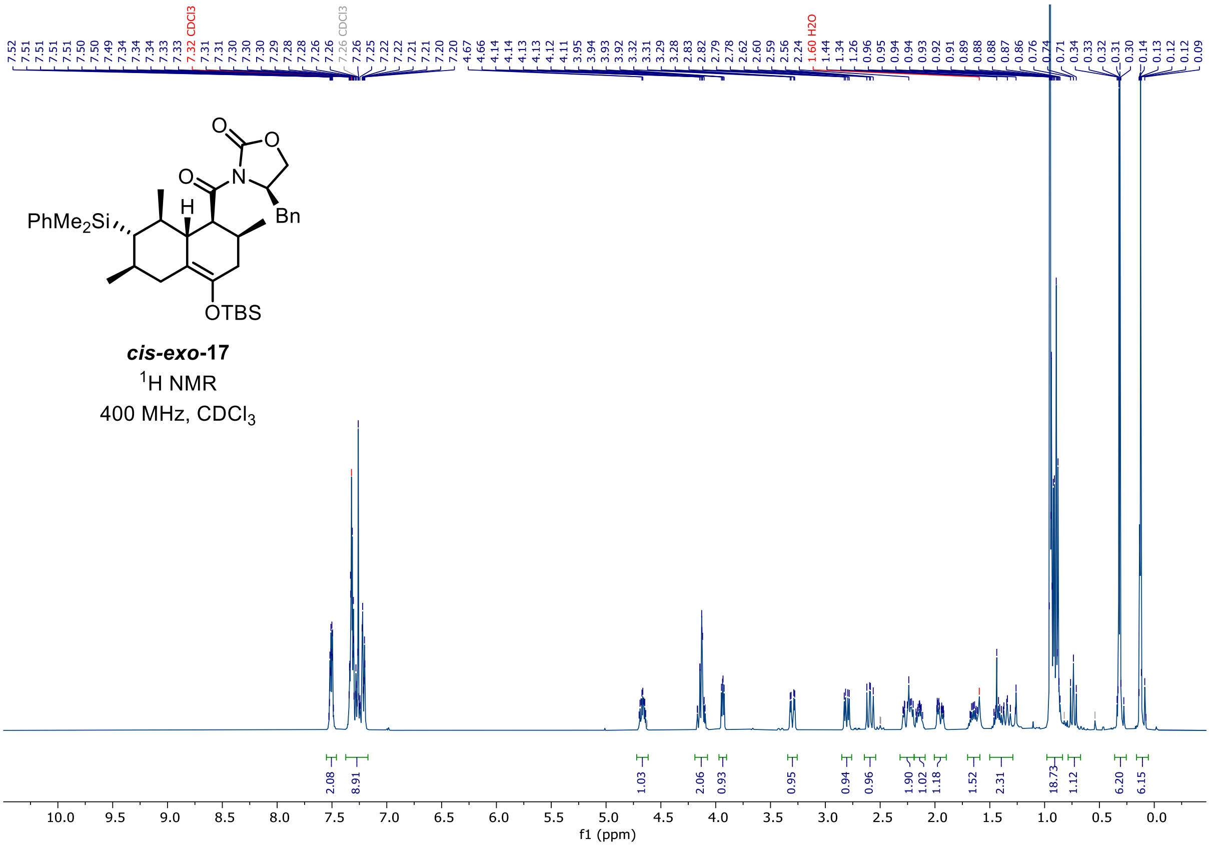

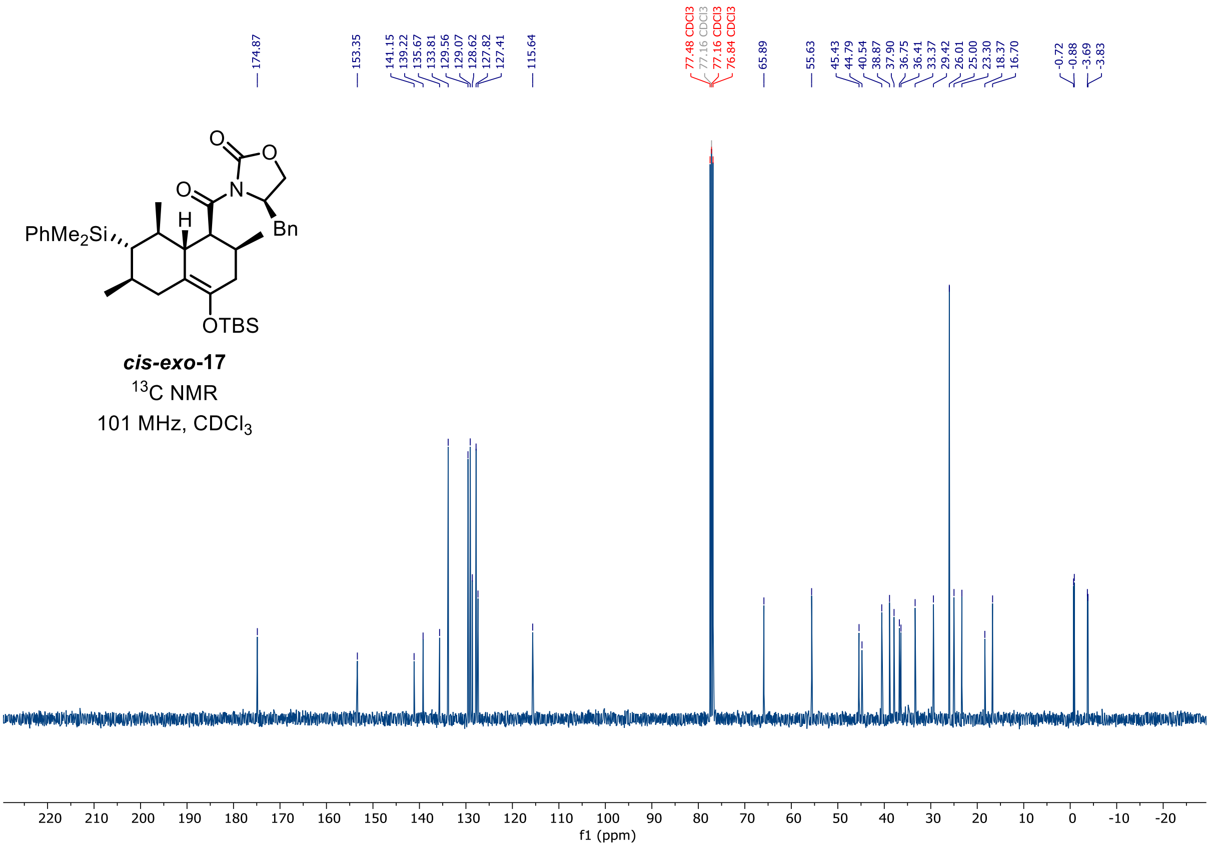

(*R*)-4-Benzyl-3-((1*S*,2*R*,6*R*,7*S*,8*S*,8a*S*)-4-((*tert*-butyldimethylsilyl)oxy)-7-(dimethyl(phenyl)silyl)-2,6,8-trimethyl-1,2,3,5,6,7,8,8a-octahydronaphthalene-1-carbonyl)oxazolidin-2-one *trans-exo*-17

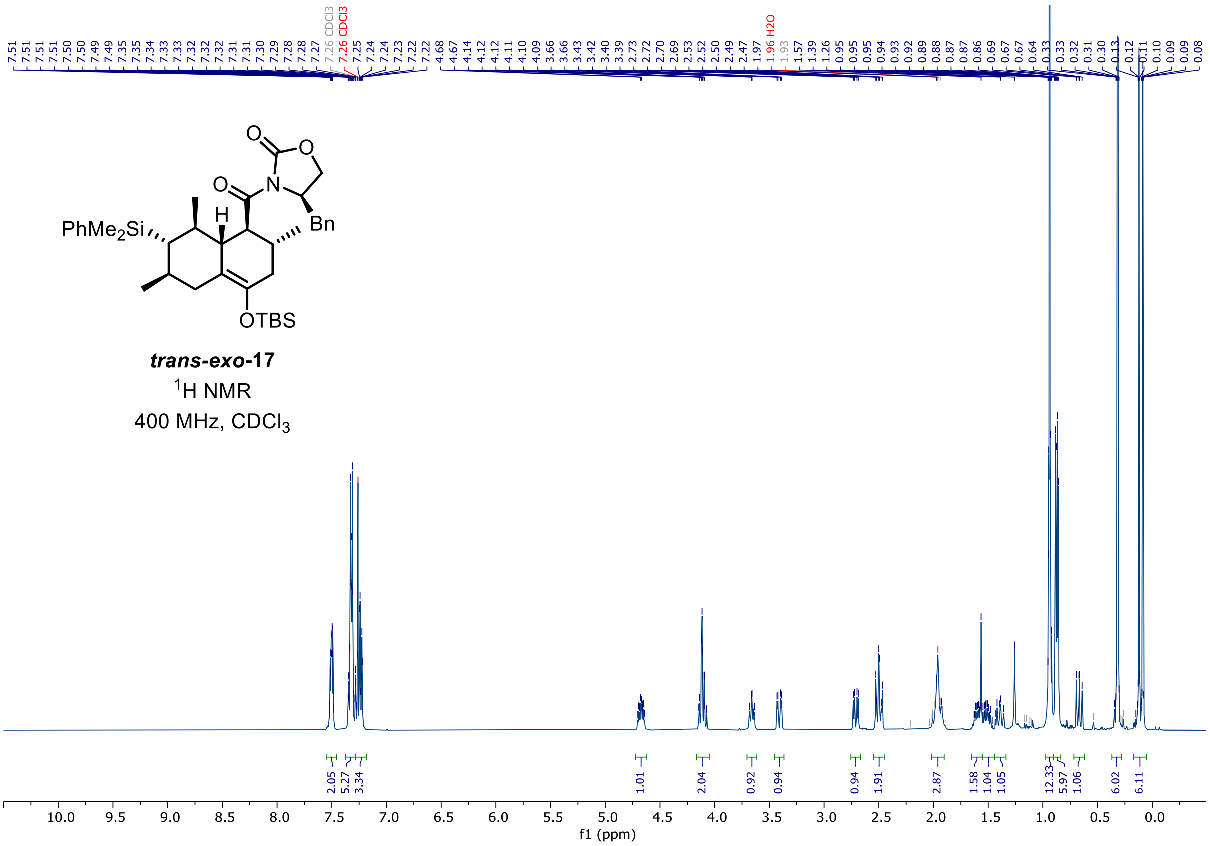

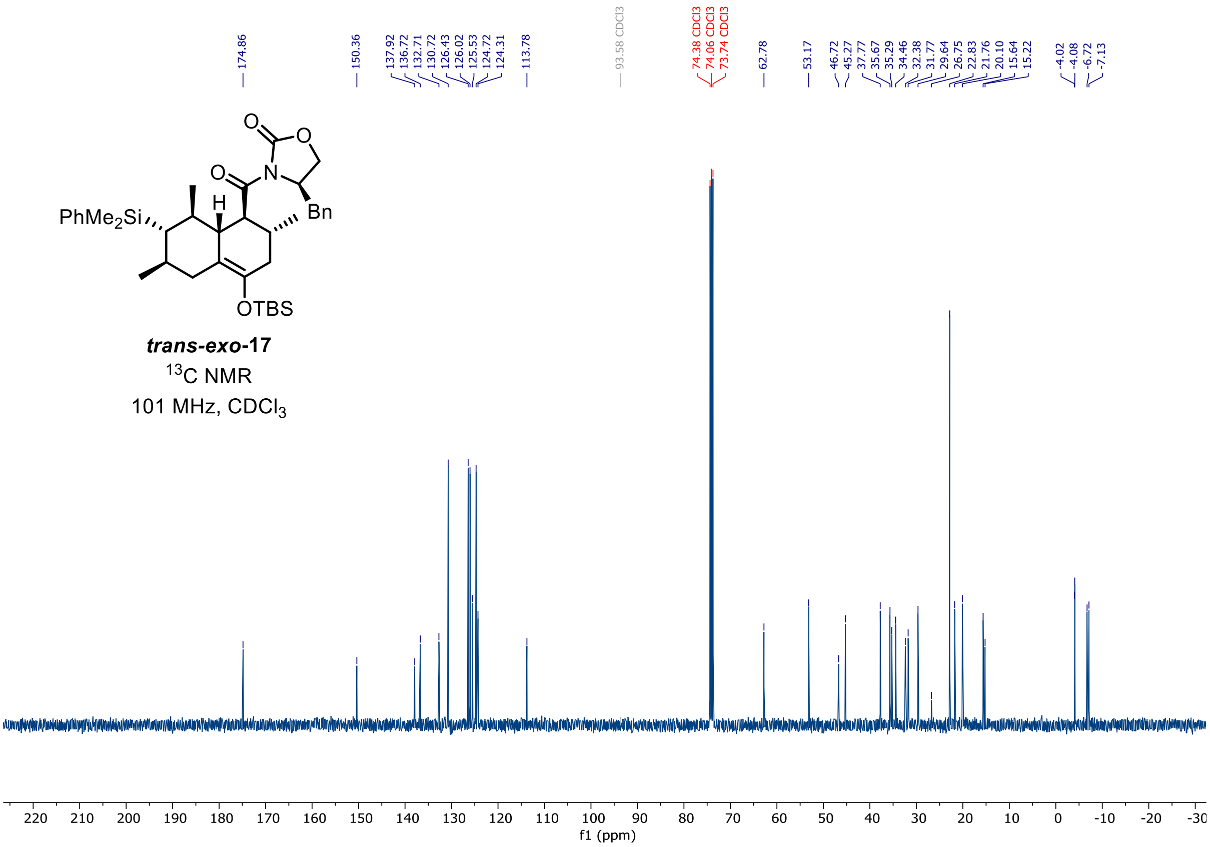

_S_-Ethyl (1_S_,2_S_,4a_S_,6_R_,7_S_,8_S_,8a_R_)-7-(dimethyl(phenyl)silyl)-2,6,8-trimethyl-4-oxodecahydronaphthalene-1-carbothioate 18

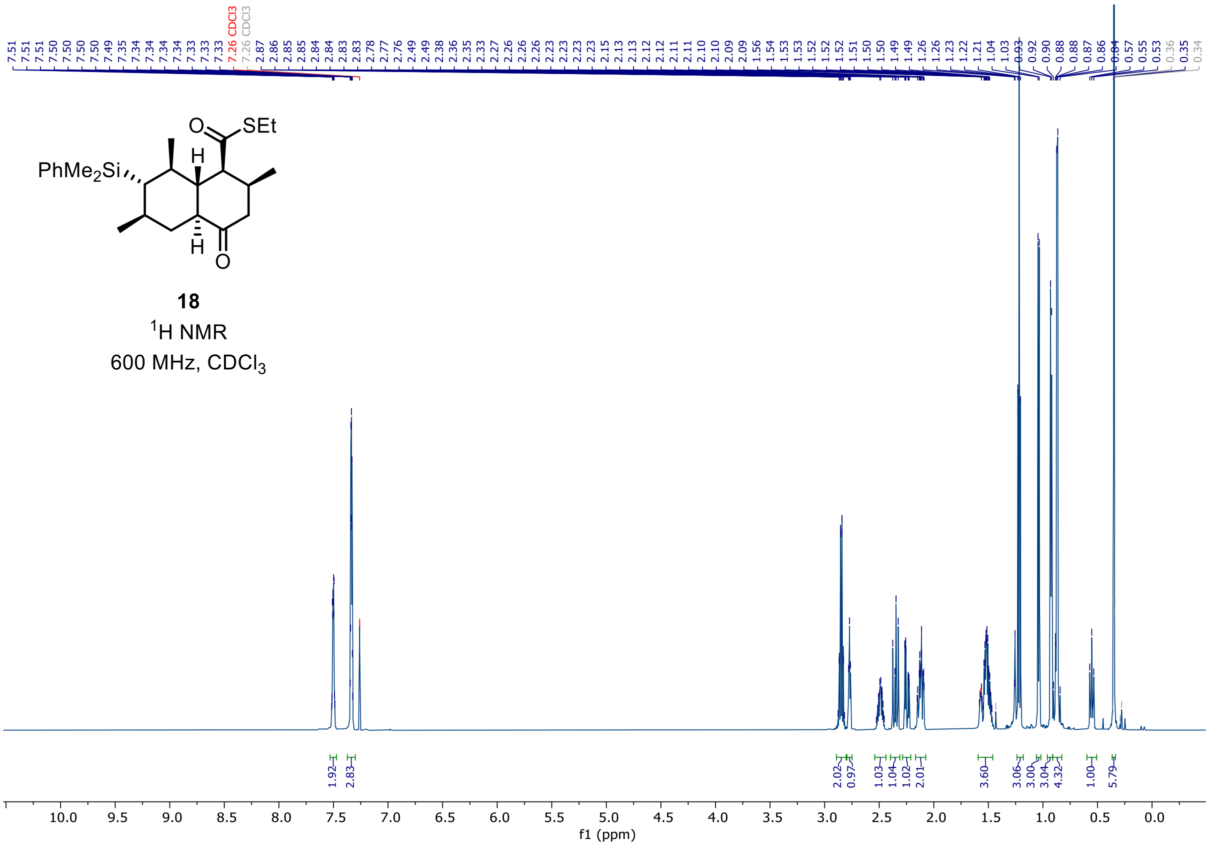

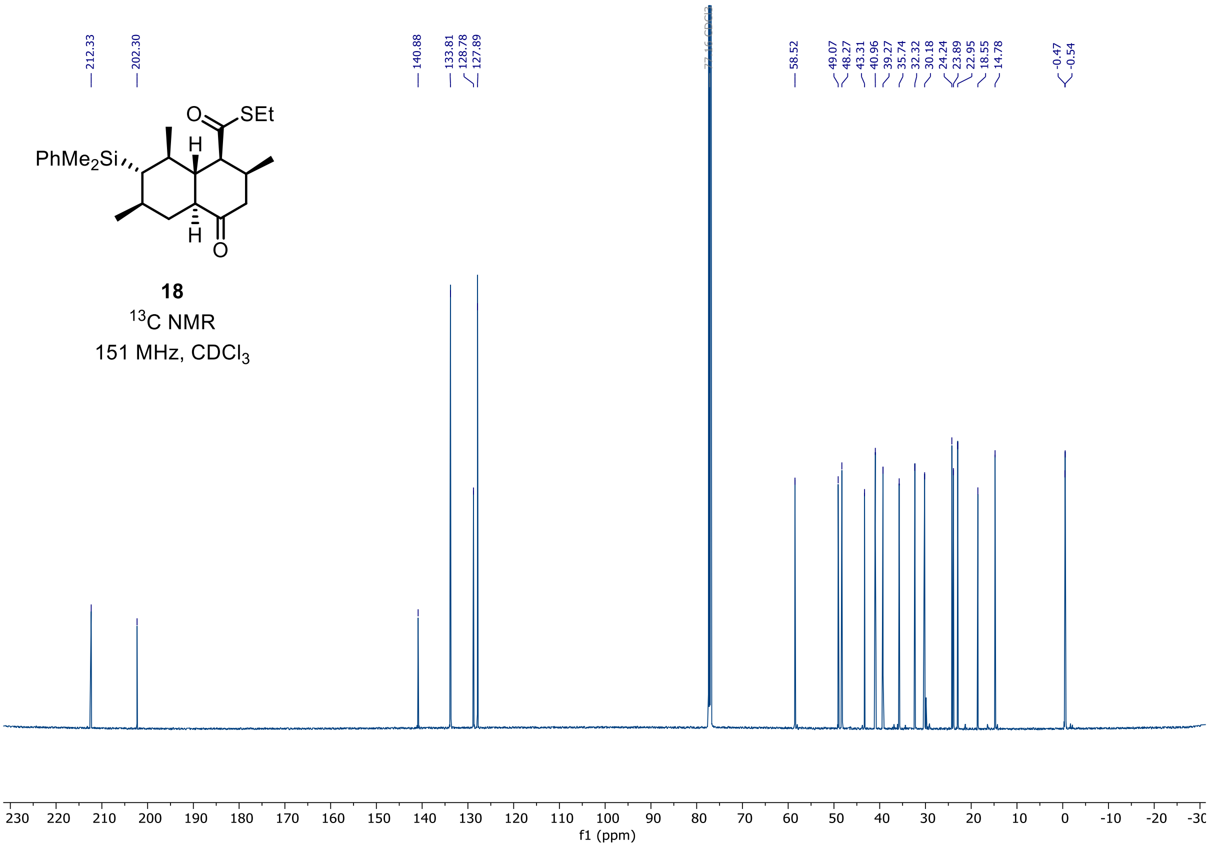

Methyl (2*E*,4*E*)-5-((1*R*,2*S*,4a*S*,6*R*,7*S*,8*S*,8a*R*)-7-(dimethyl(phenyl)silyl)-2,6,8-trimethyl-4-oxodecahydronaphthalen-1-yl)penta-2,4-dienoate 20

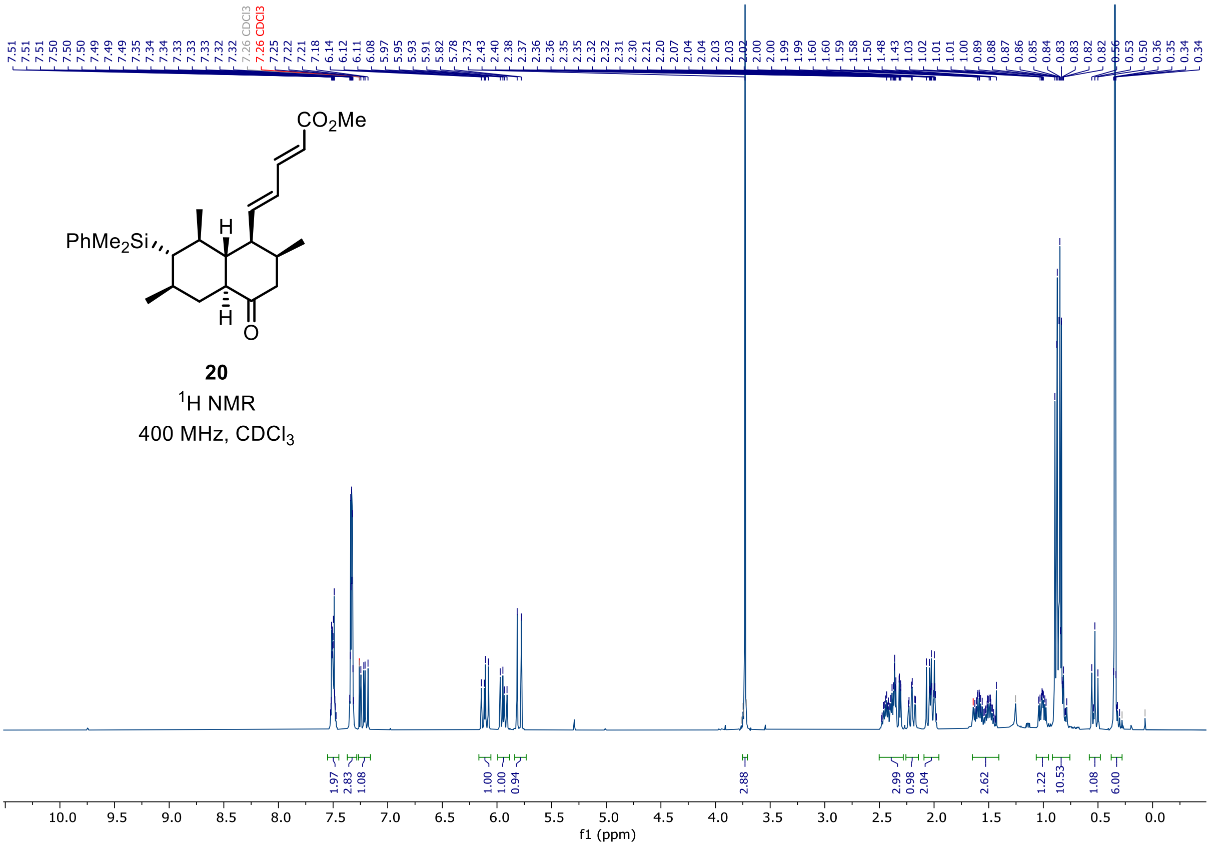

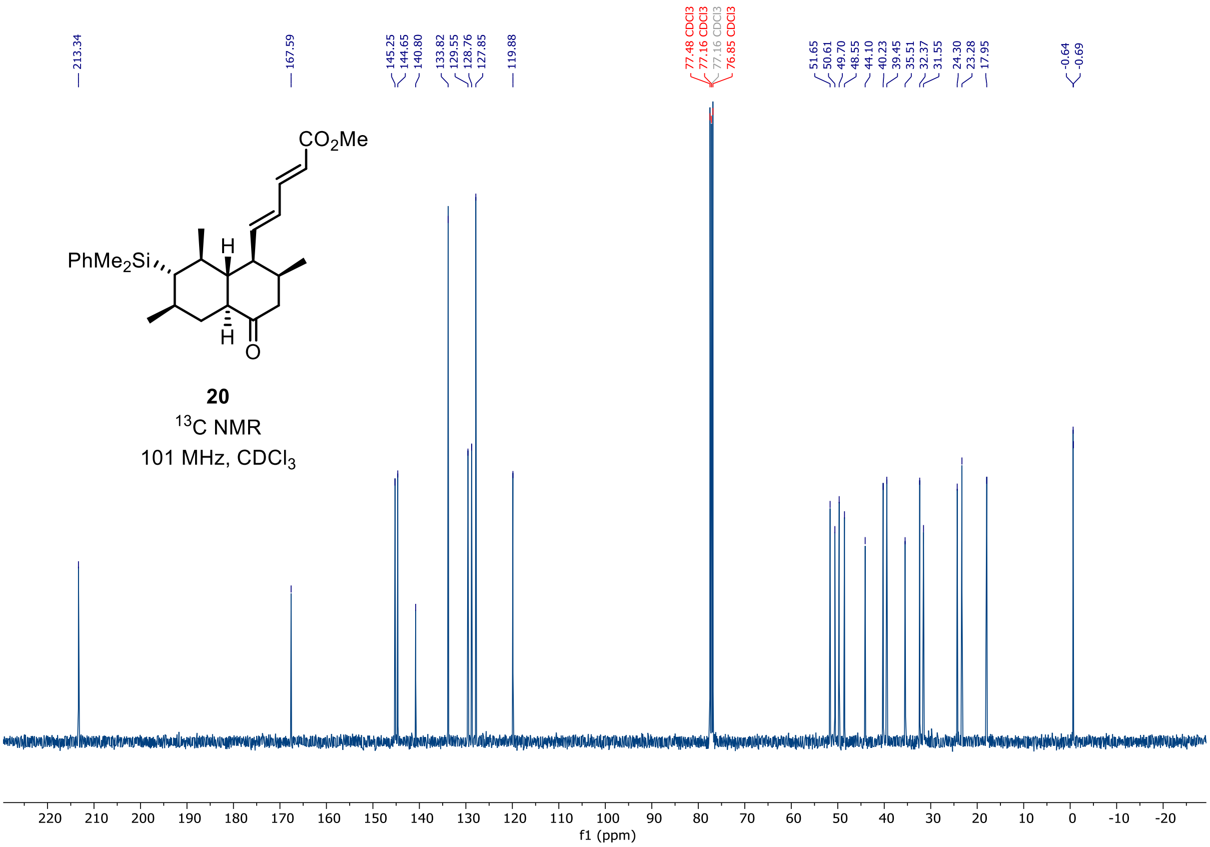

Methyl (2*E*,4*E*)-5-((1*S*,2*S*,4a*R*,6*R*,7*S*,8*S*,8a*S*)-7-(dimethyl(phenyl)silyl)-2,6,8-trimethyl-1,2,4a,5,6,7,8,8a-octahydronaphthalen-1-yl)penta-2,4-dienoate 21

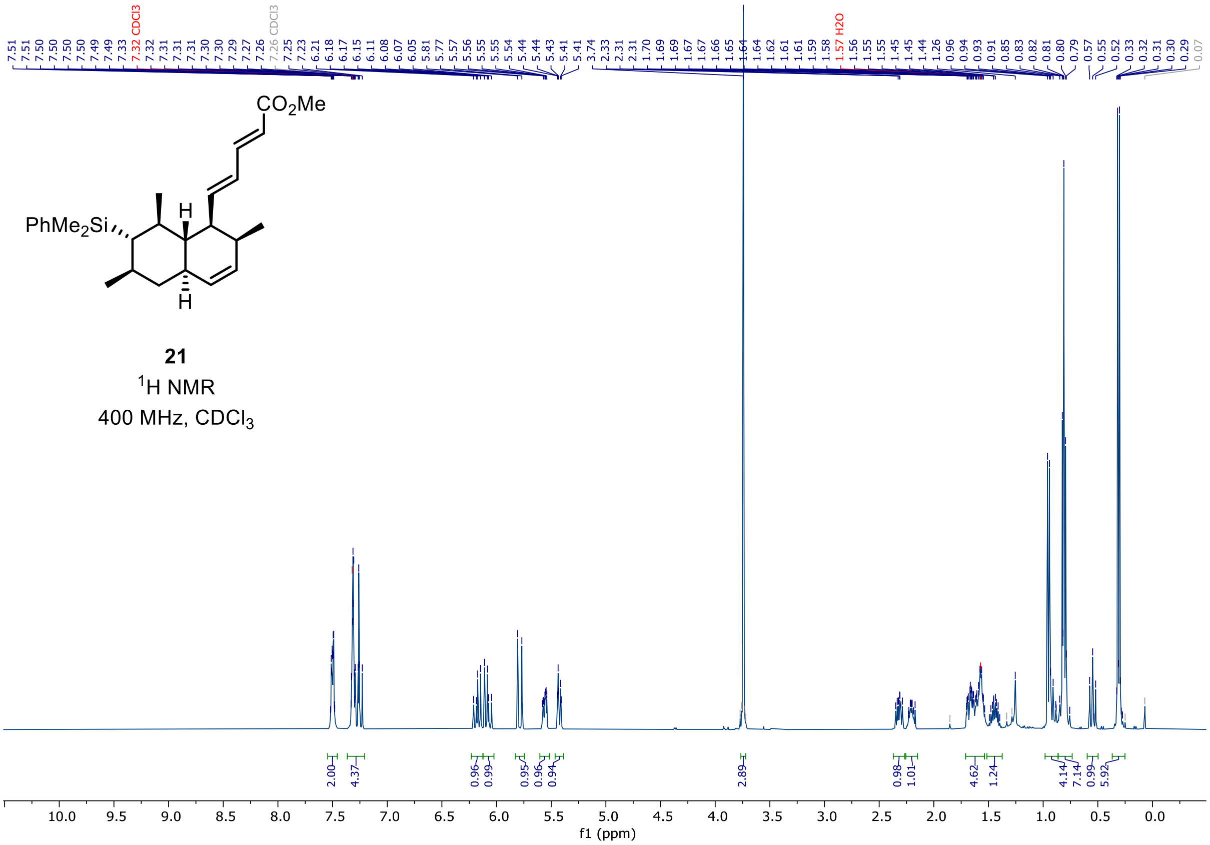

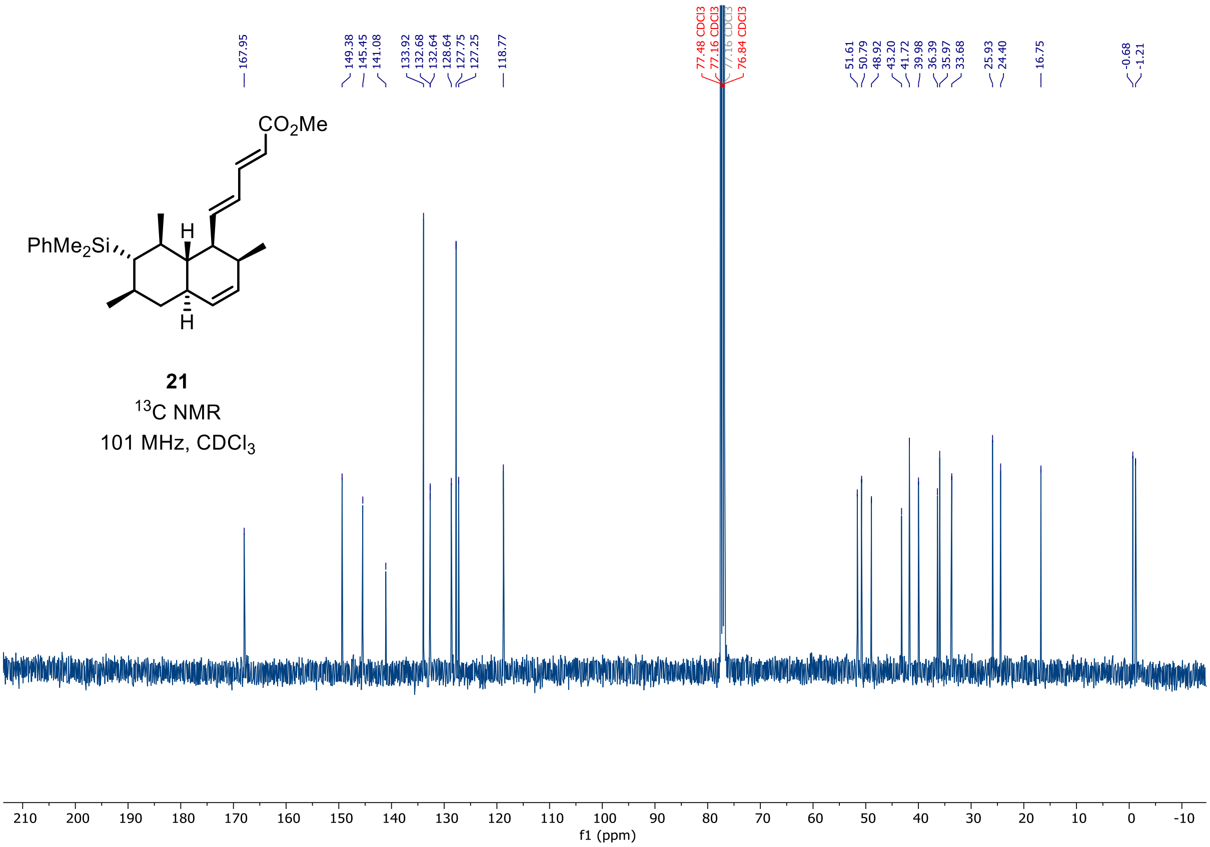

Methyl (2*E*,4*E*)-5-((1*S*,2*S*,4a*R*,6*R*,7*S*,8*S*,8a*S*)-7-hydroxy-2,6,8-trimethyl-1,2,4a,5,6,7,8,8a-octahydronaphthalen-1-yl)penta-2,4-dienoate (+)-Coprophilin (1)

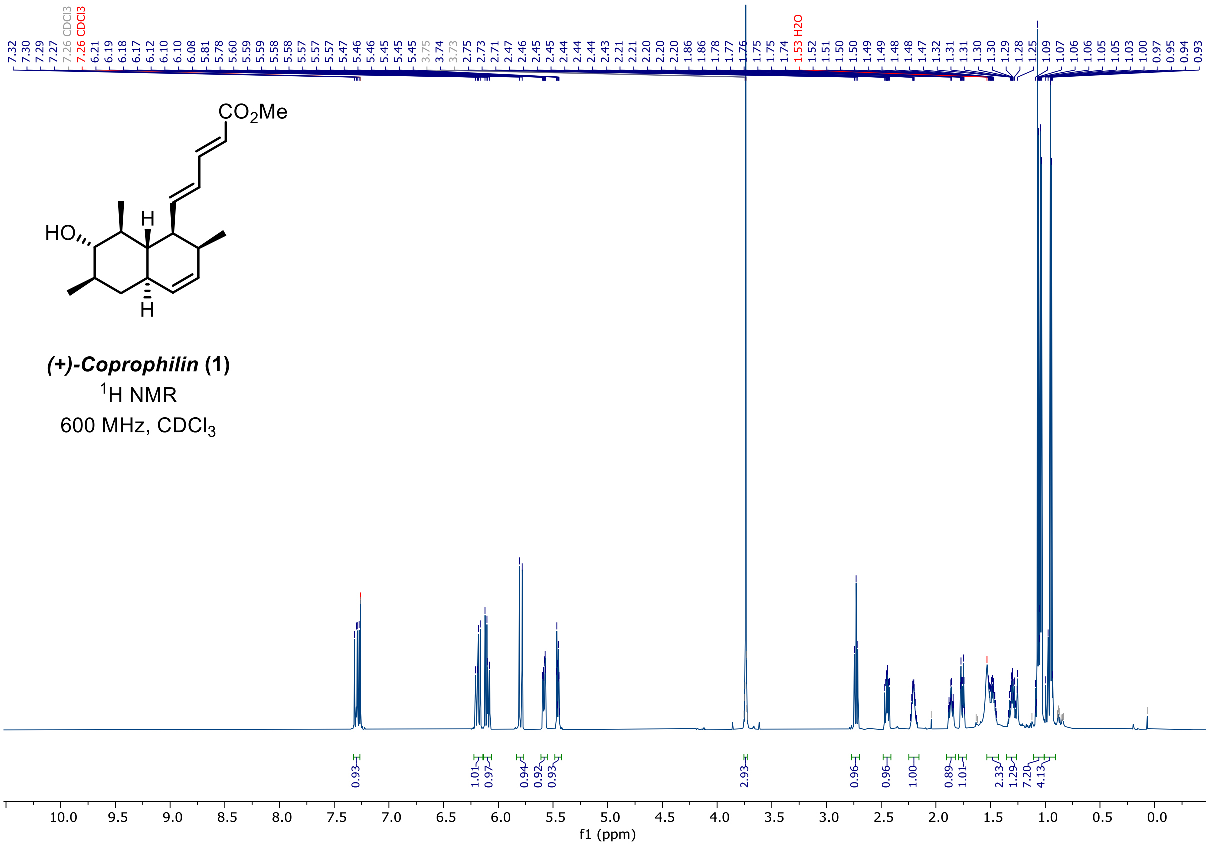

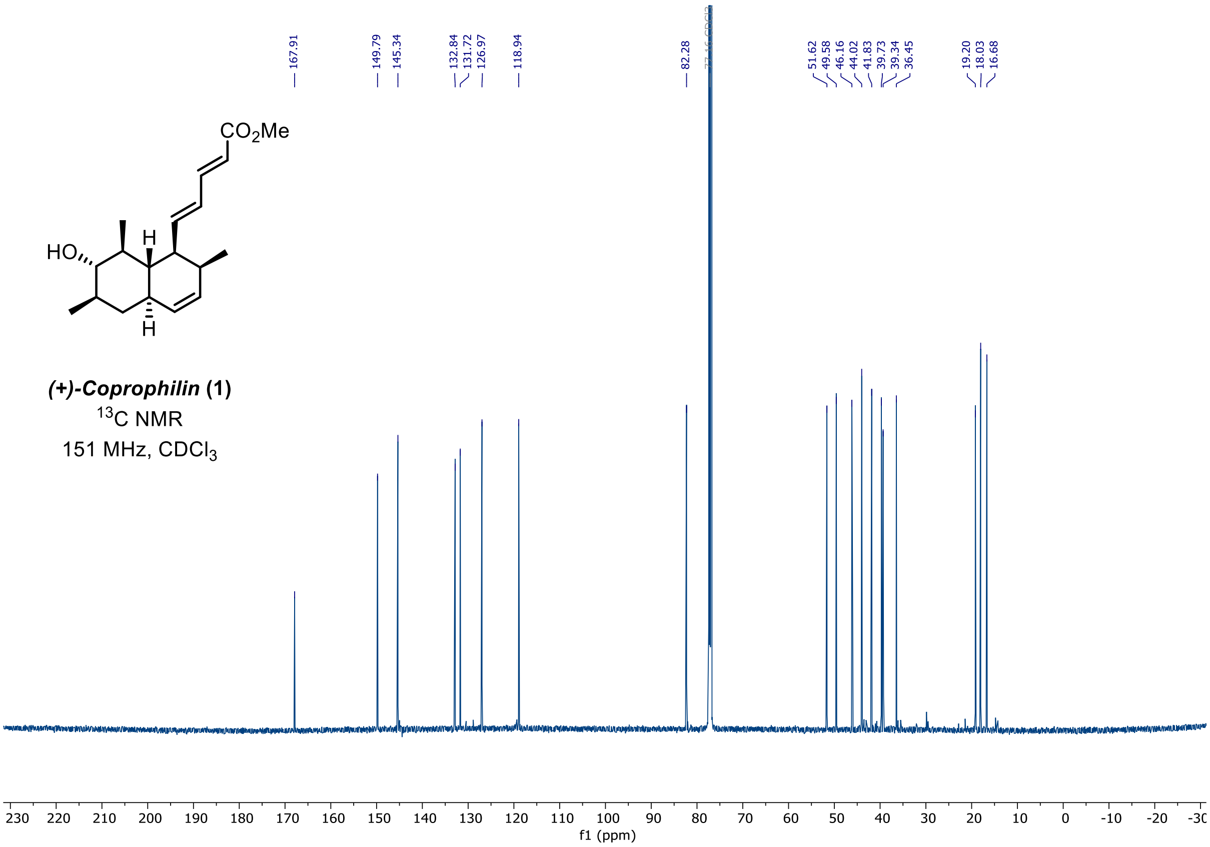

***tert*-Butyl (2*E*,4*E*)-5-((1*R*,2*S*,4a*S*,6*R*,7*S*,8*S*,8a*R*)-7-(dimethyl(phenyl)silyl)-2,6,8-trimethyl-4-oxodecahydronaphthalen-1-yl)-2-methylpenta-2,4-dienoate 23**

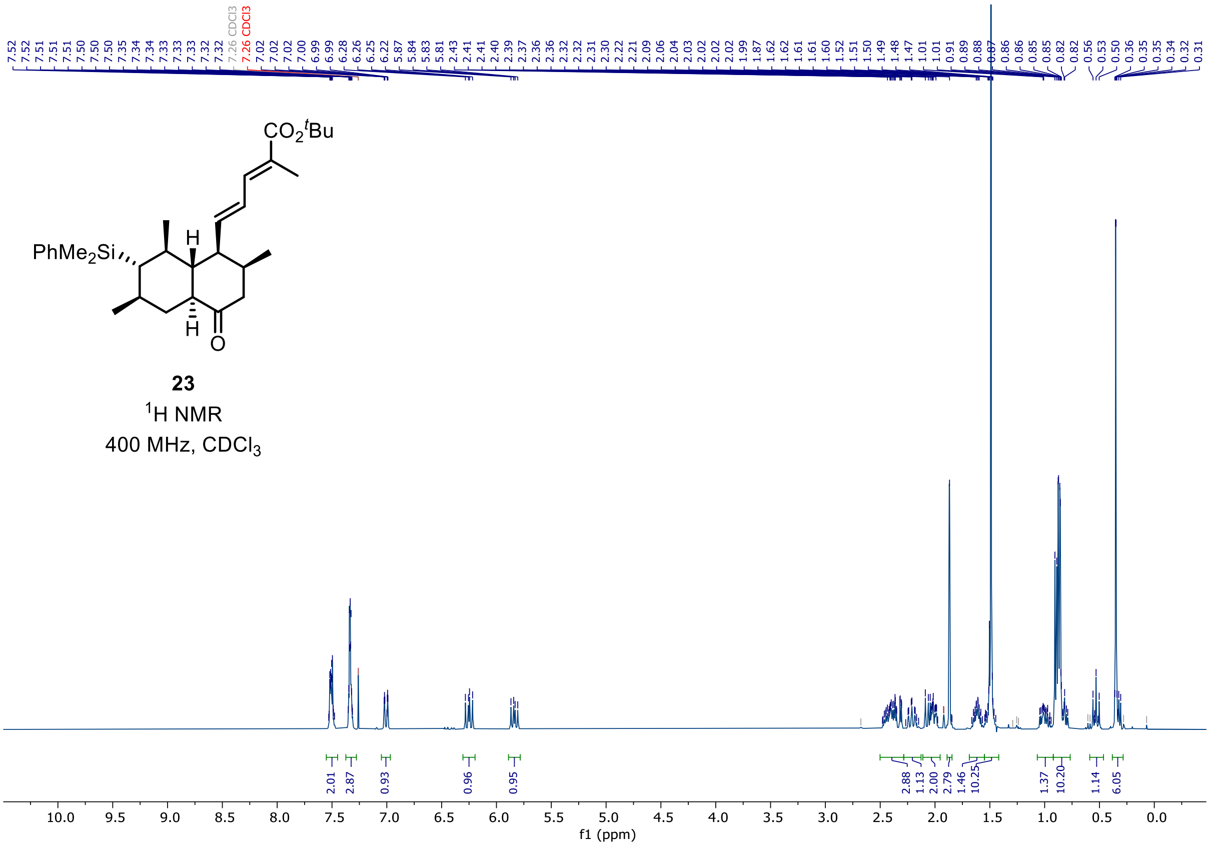

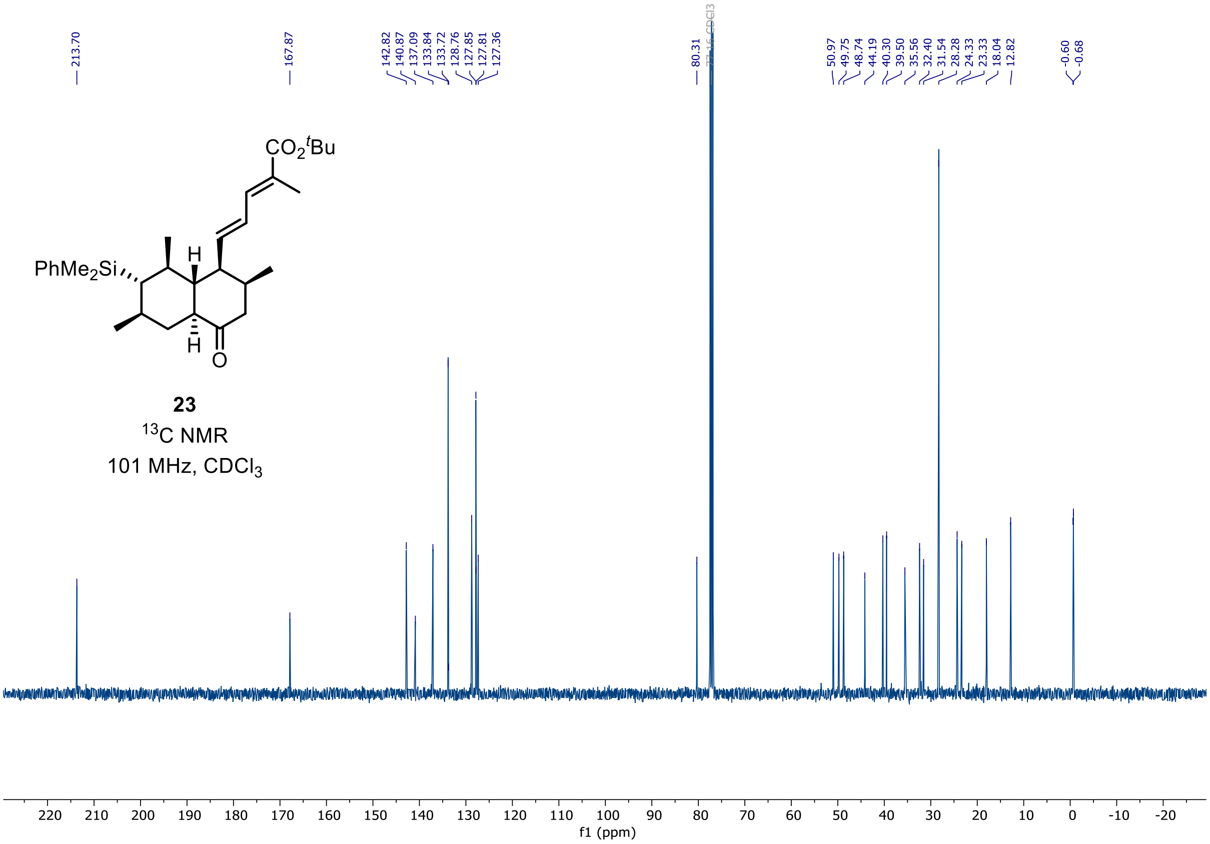

tert-Butyl (2_E_,4_E_)-5-((1_S_,2_S_,4a_R_,6_R_,7_S_,8_S_,8a_S_)-7-(dimethyl(phenyl)silyl)-2,6,8-trimethyl-1,2,4a,5,6,7,8,8a-octahydronaphthalen-1-yl)-2-methylpenta-2,4-dienoate 24

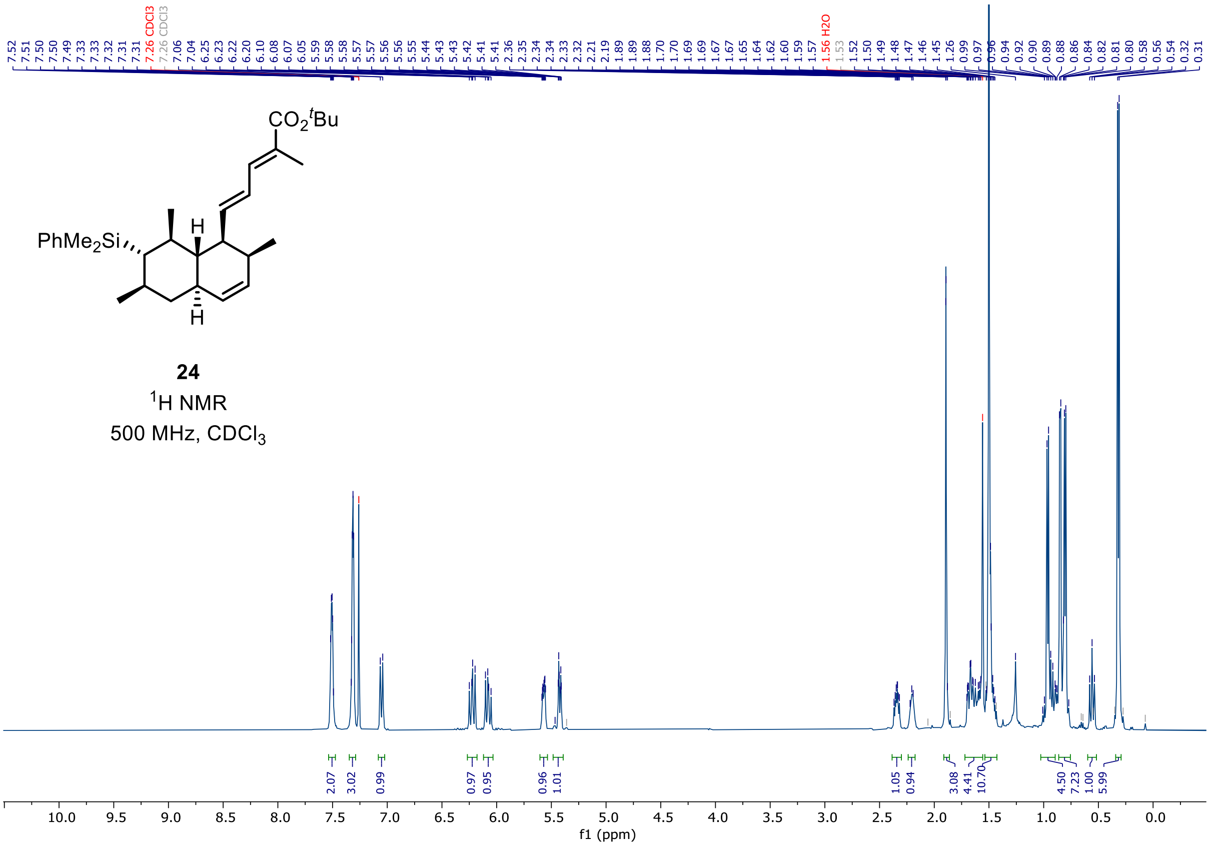

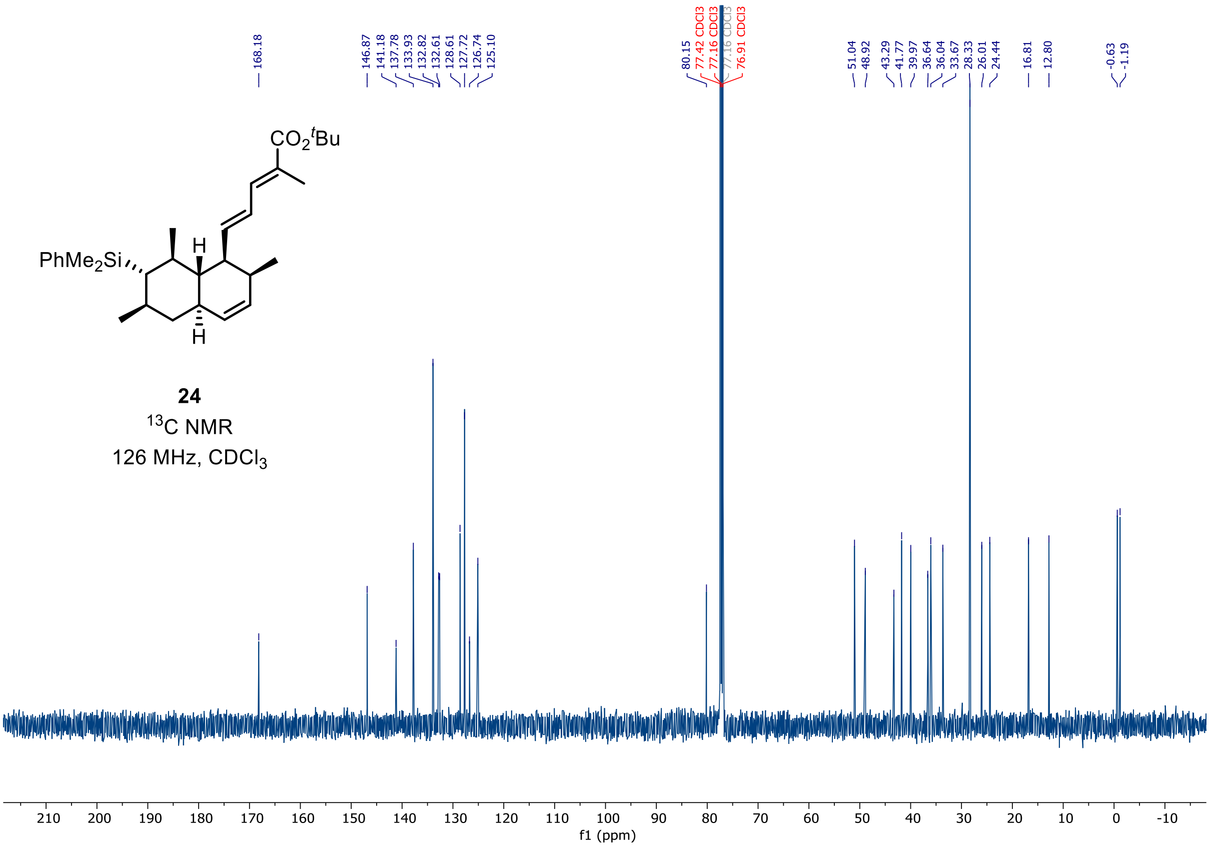

(2*E*,4*E*)-5-((1*S*,2*S*,4a*R*,6*R*,7*S*,8*S*,8a*S*)-7-Hydroxy-2,6,8-trimethyl-1,2,4a,5,6,7,8,8a-octahydronaphthalen-1-yl)-2-methylpenta-2,4-dienoic acid (+)-Trichodermic acid (2)

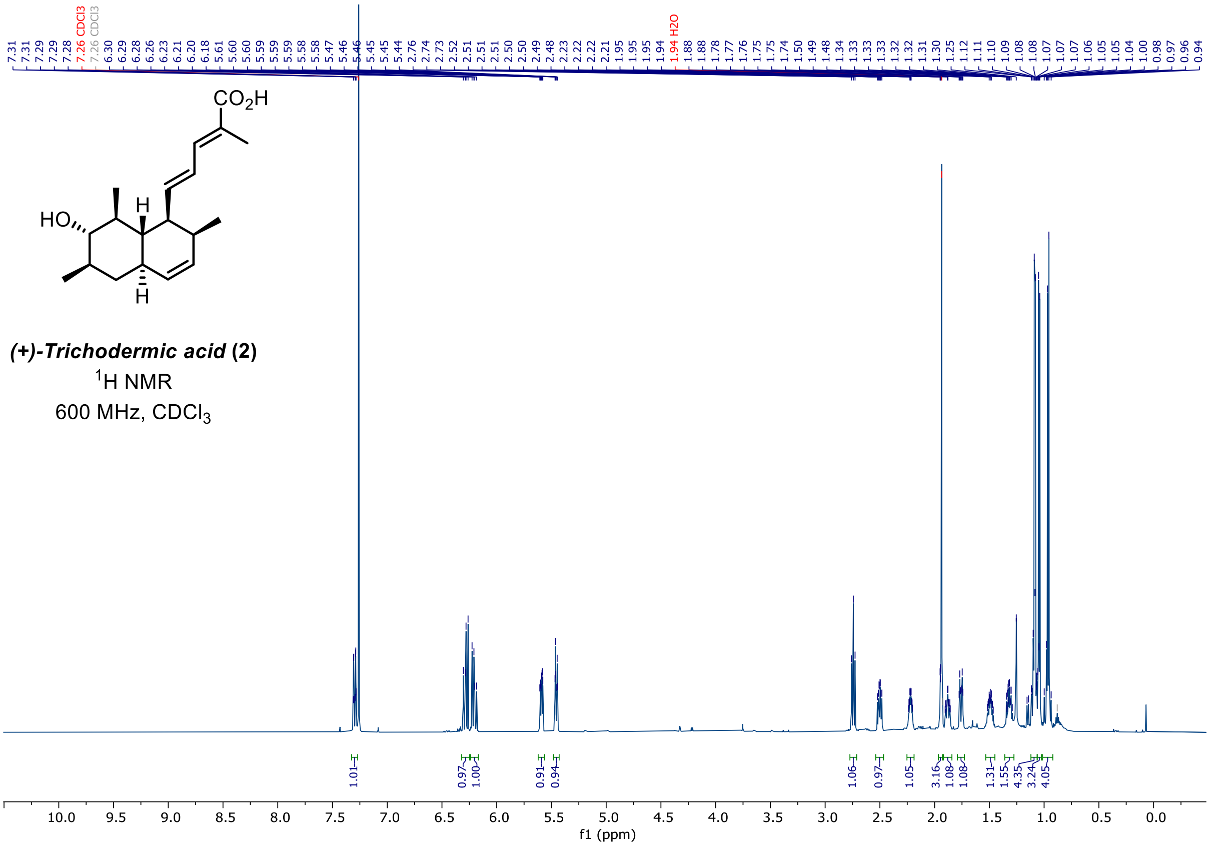

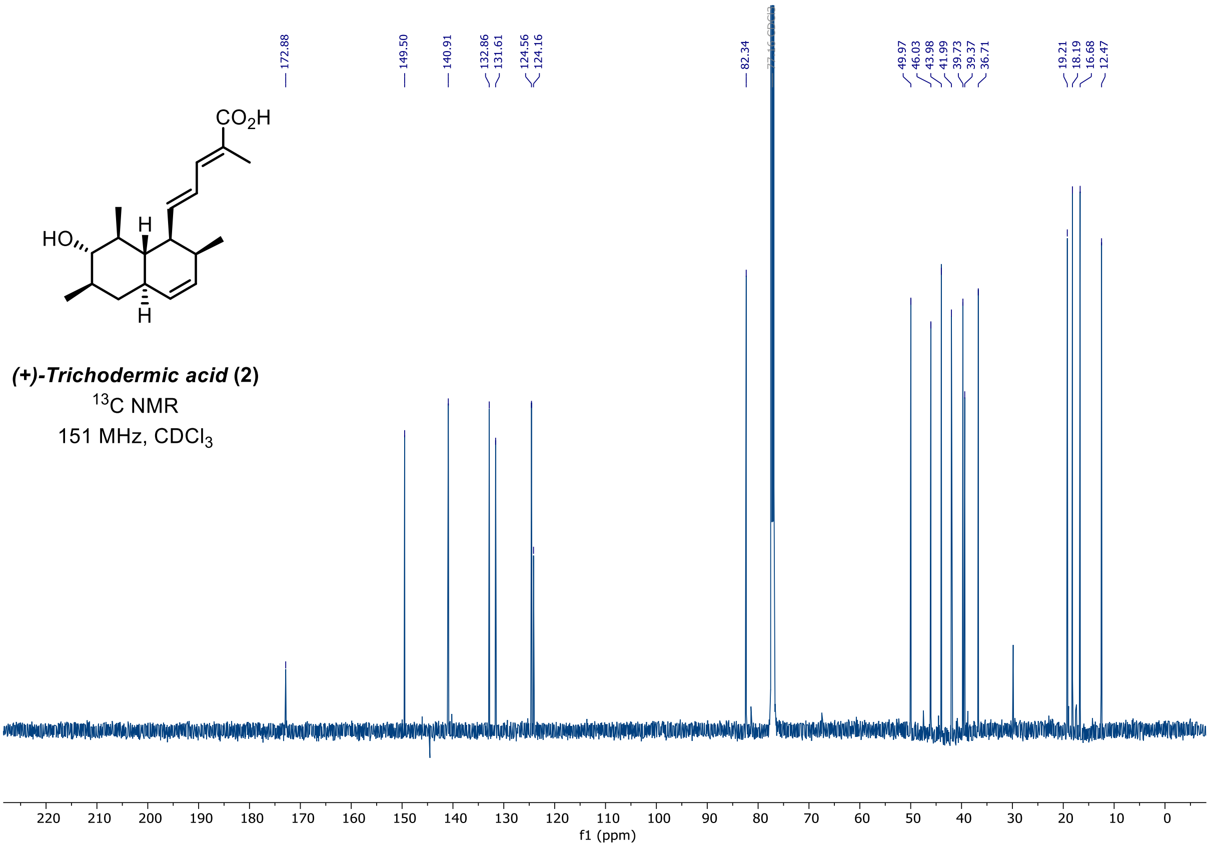

tert-Butyl (2_E_,4_E_)-5-((1_R_,4a_S_,6_R_,7_S_,8_S_,8a_R_)-7-(dimethyl(phenyl)silyl)-2,6,8-trimethyl-4-oxo-1,4,4a,5,6,7,8,8a-octahydronaphthalen-1-yl)-2-methylpenta-2,4-dienoate 25

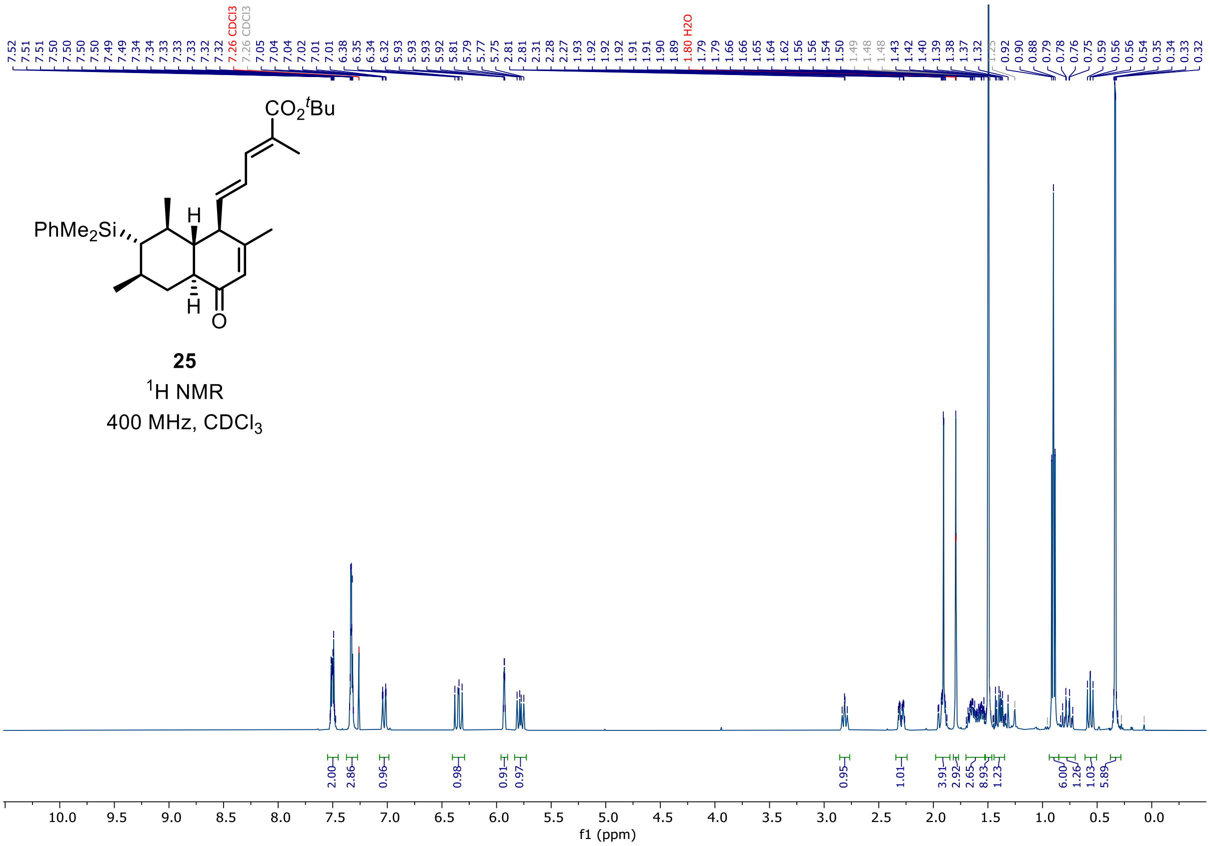

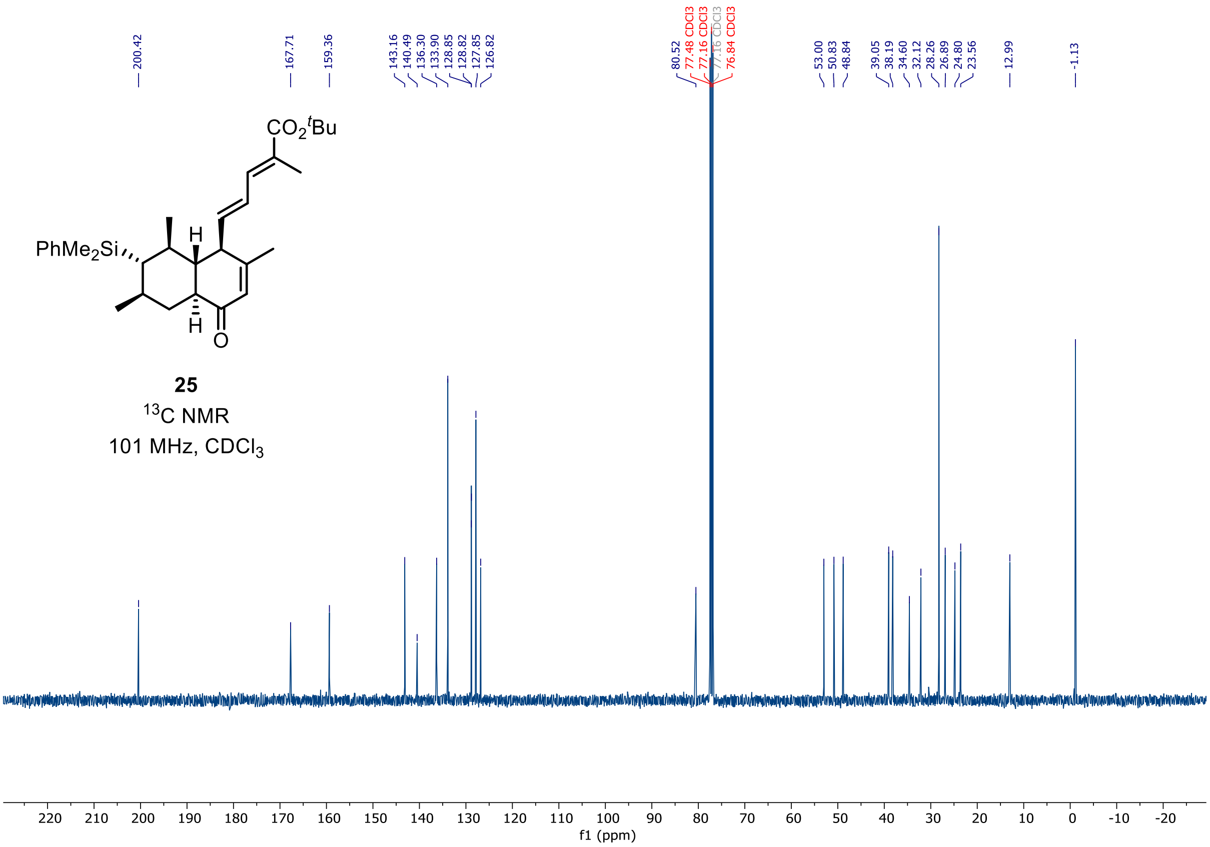

(2E,4E)-5-((1R,4aS,6R,7S,8S,8aS)-7-Hydroxy-2,6,8-trimethyl-4-oxo-1,4,4a,5,6,7,8,8a-octahydronaphthalen-1-yl)-2-methylpenta-2,4-dienoic acid (+)-Trichodermic acid E (5)

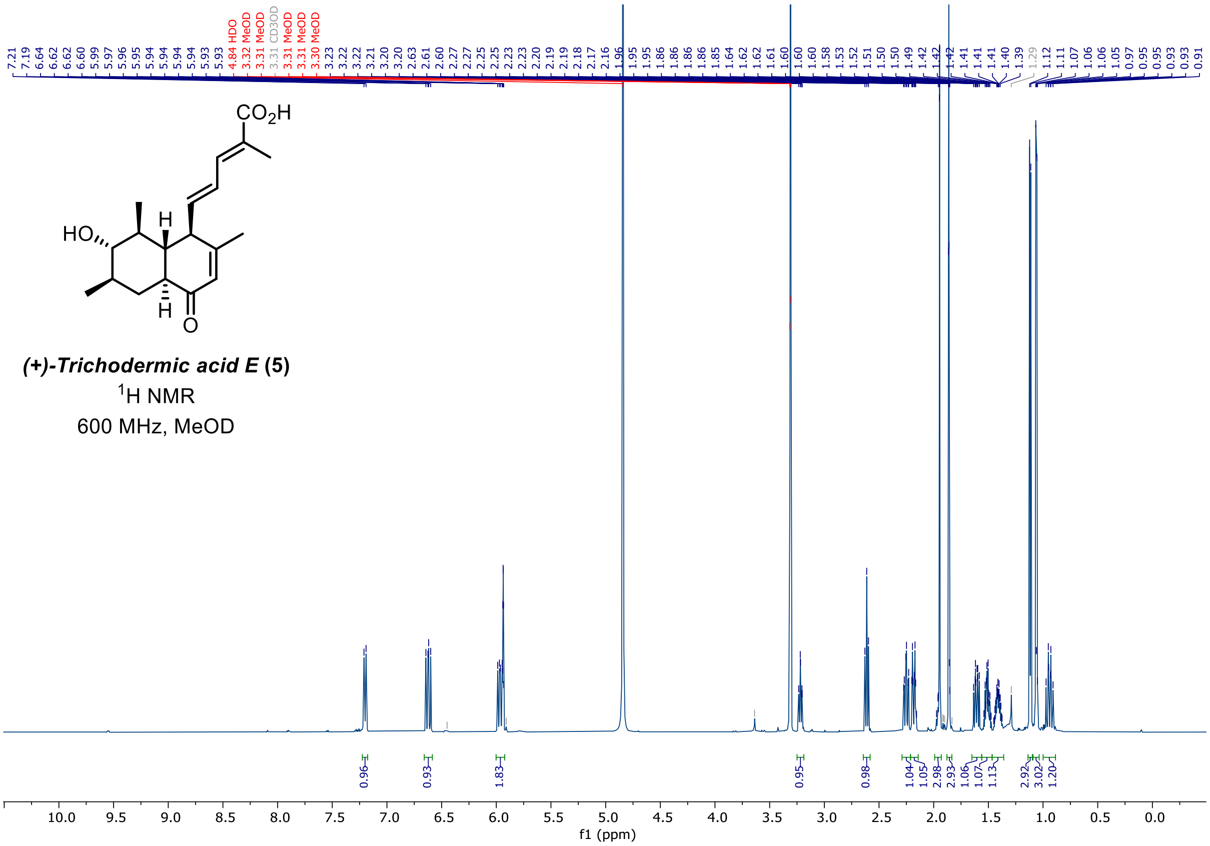

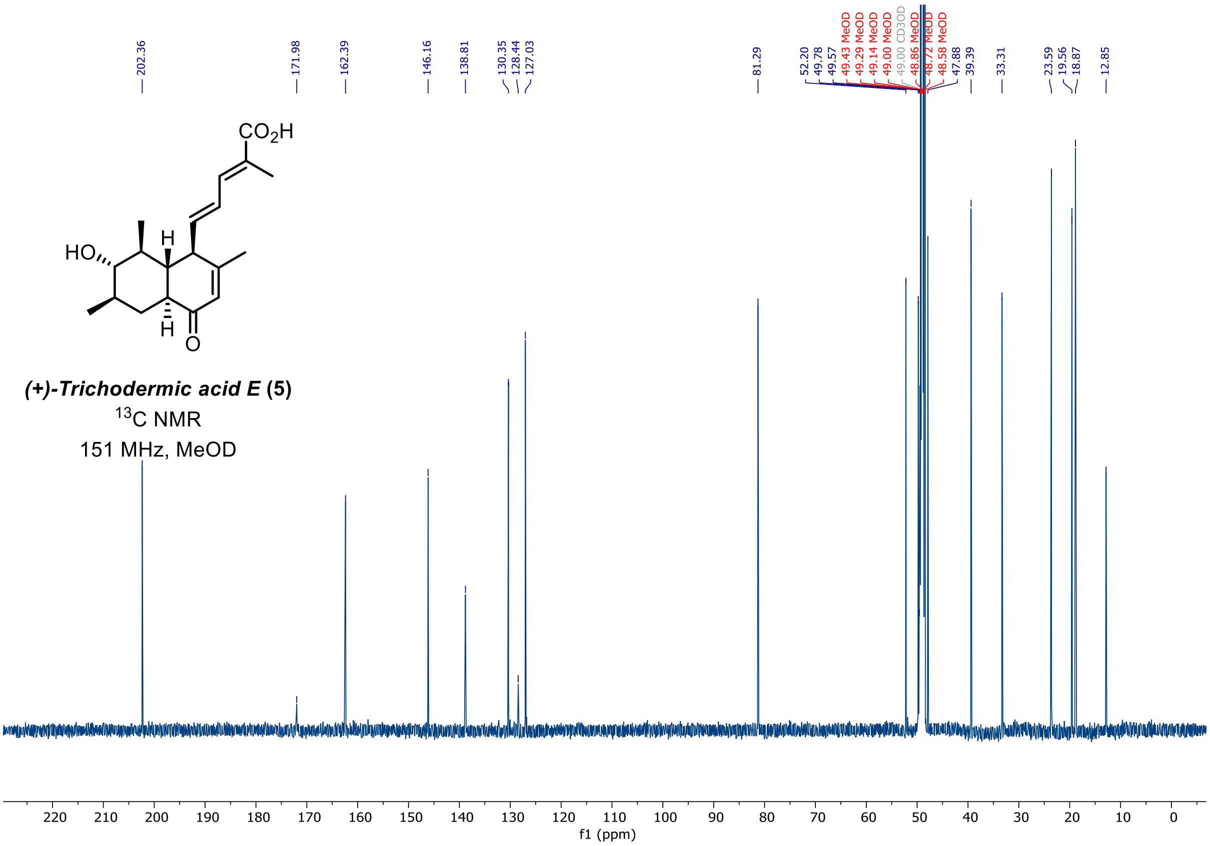

(2*E*,4*E*)-5-((1*R*,4*R*,4a*S*,6*R*,7*S*,8*S*,8a*S*)-4,7-Dihydroxy-2,6,8-trimethyl-1,4,4a,5,6,7,8,8a-octahydronaphthalen-1-yl)-2-methylpenta-2,4-dienoic acid (+)-Trichodermic acid G (6)

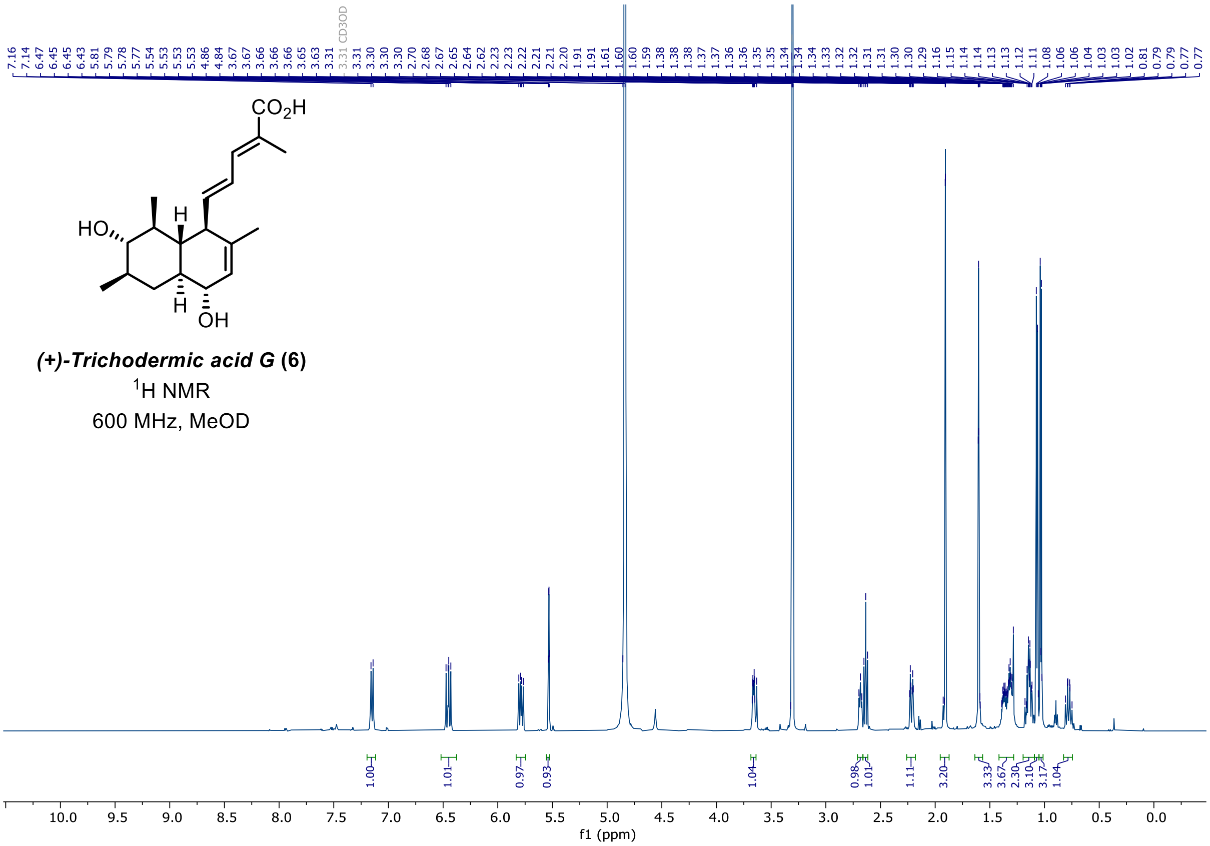

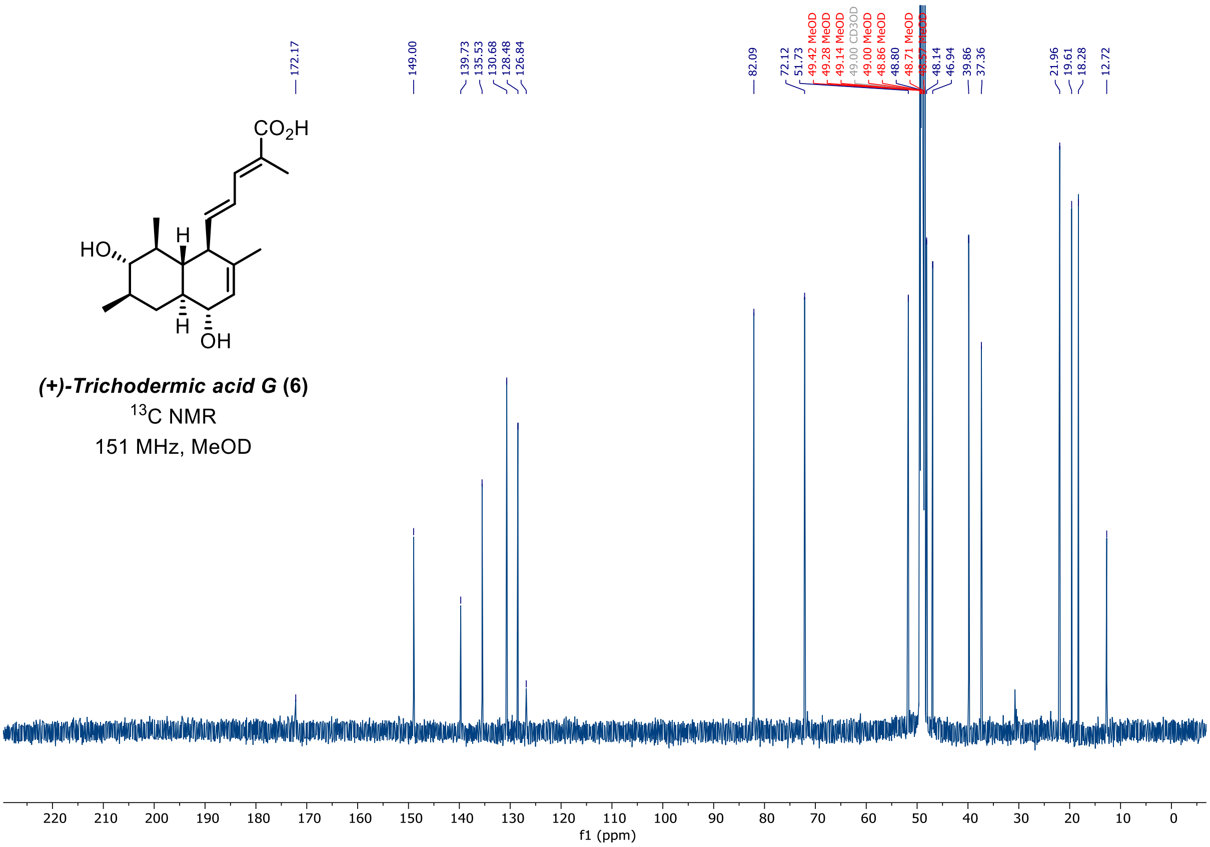

tert-Butyl (2_E_,4_E_)-5-((1_R_,2_R_,3_S_,4_S_,4a_S_,6_R_,7_S_,8_S_,8a_R_)-7-(dimethyl(phenyl)silyl)-3,4-dihydroxy-2,6,8-trimethyldecahydronaphthalen-1-yl)-2-methylpenta-2,4-dienoate

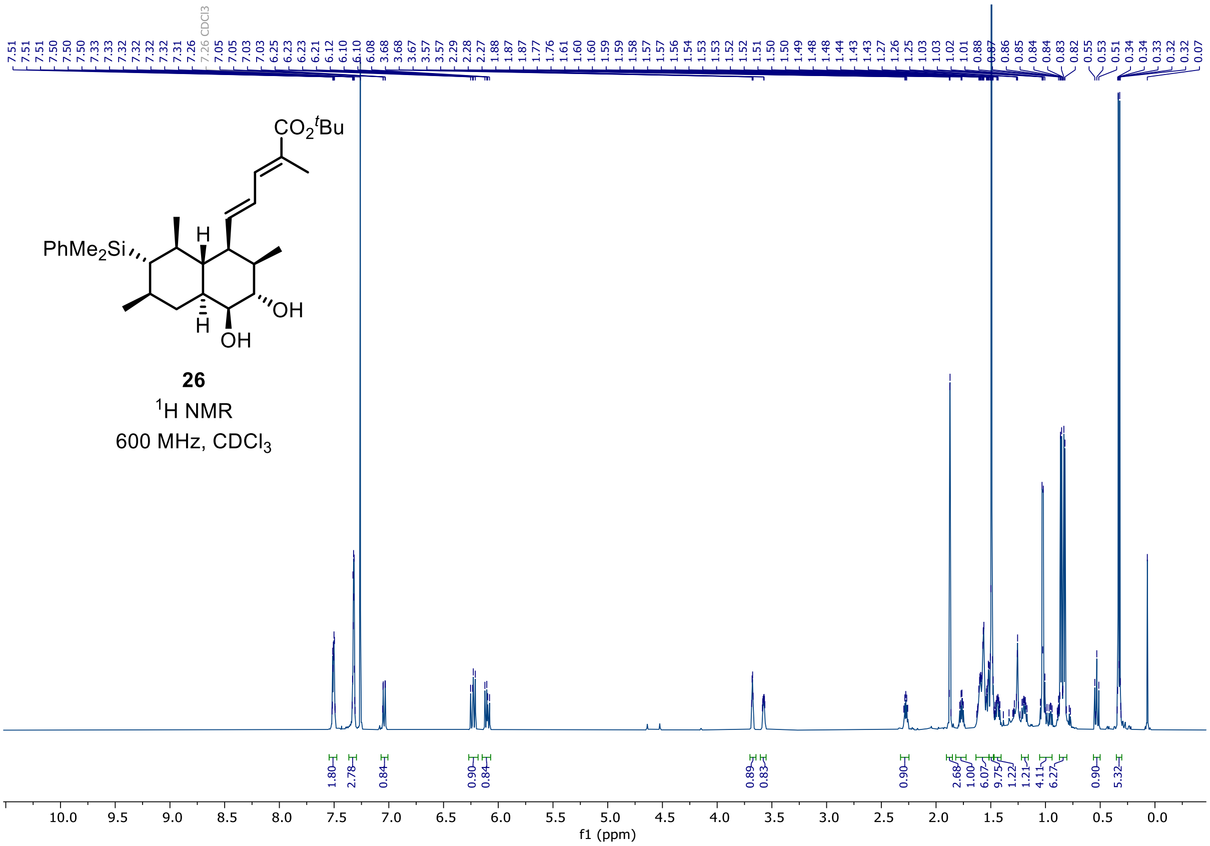

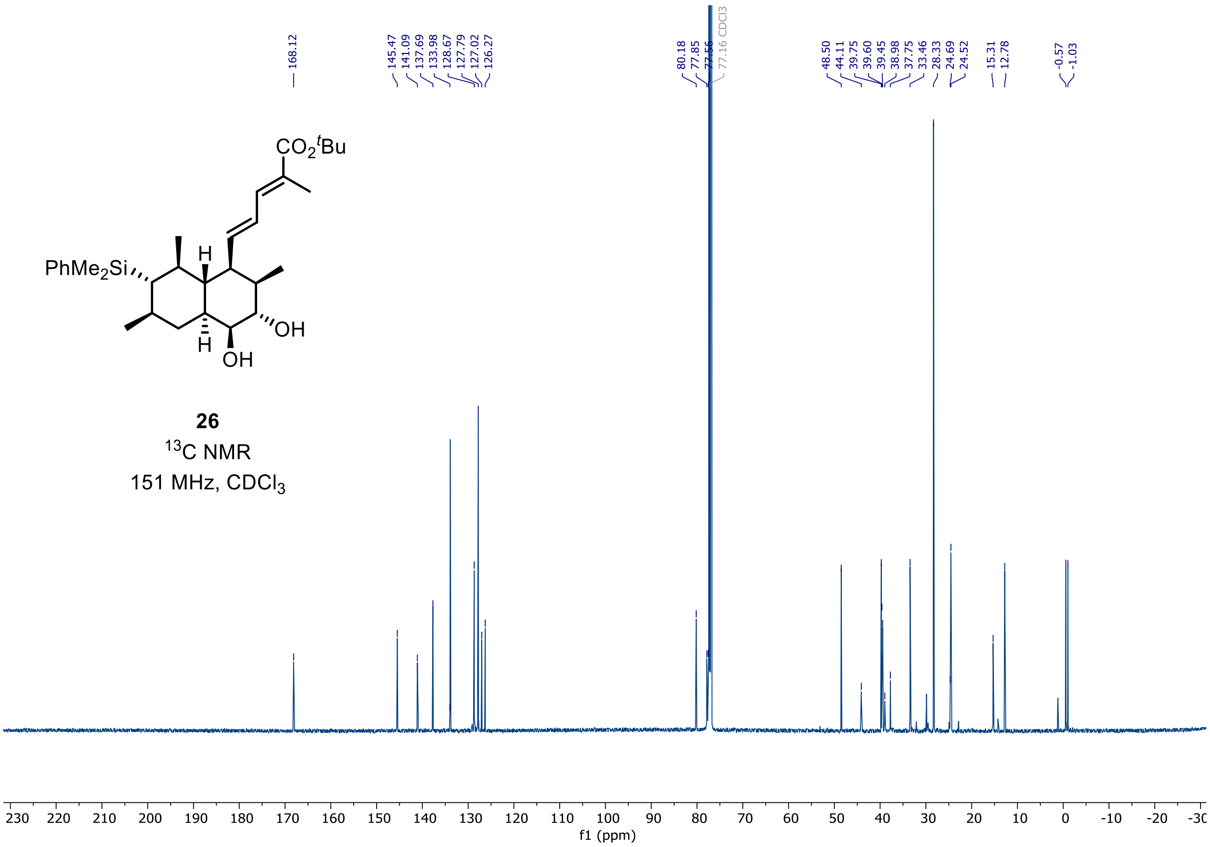

(2*E*,4*E*)-2-Methyl-5-((1*R*,2*R*,3*S*,4*S*,4a*S*,6*R*,7*S*,8*S*,8a*S*)-3,4,7-trihydroxy-2,6,8-trimethyldecahydronaphthalen-1-yl)penta-2,4-dienoic acid (-)-Trichodermic acid D (4)

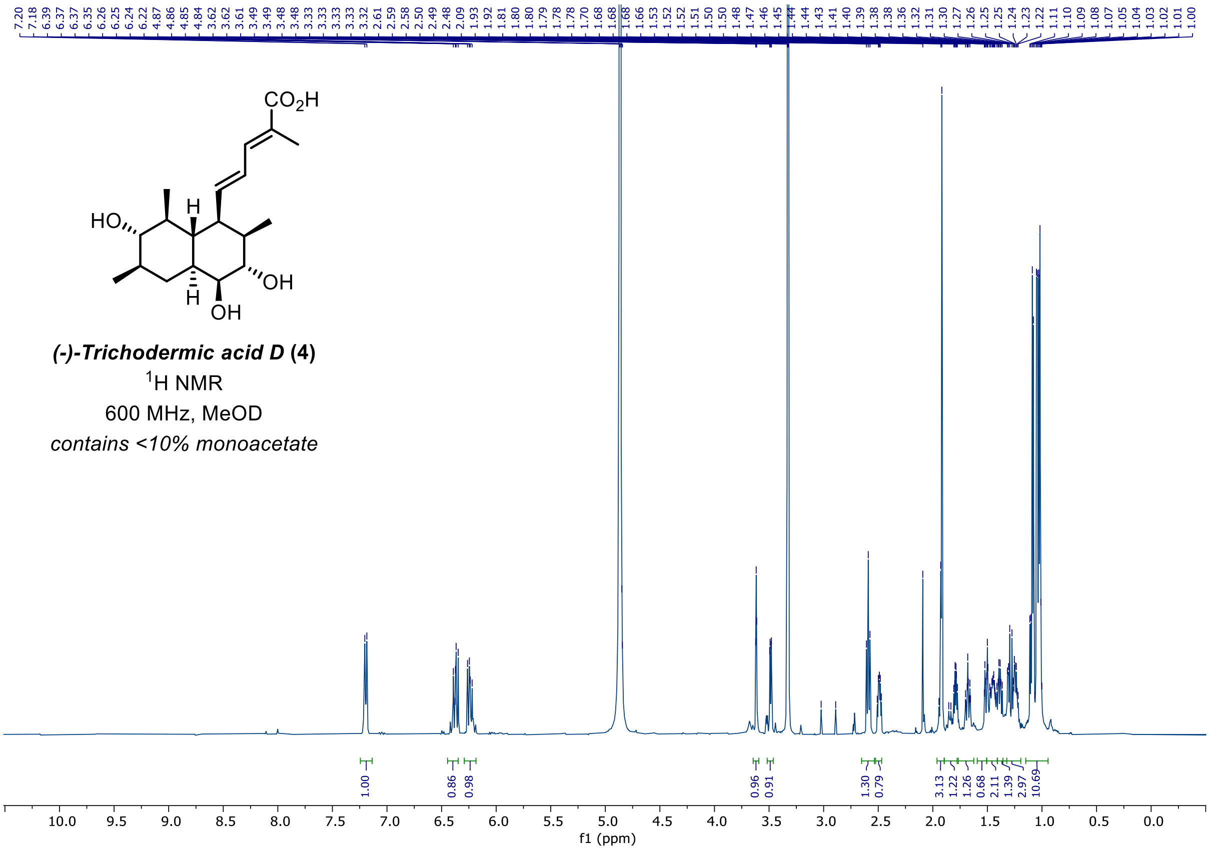

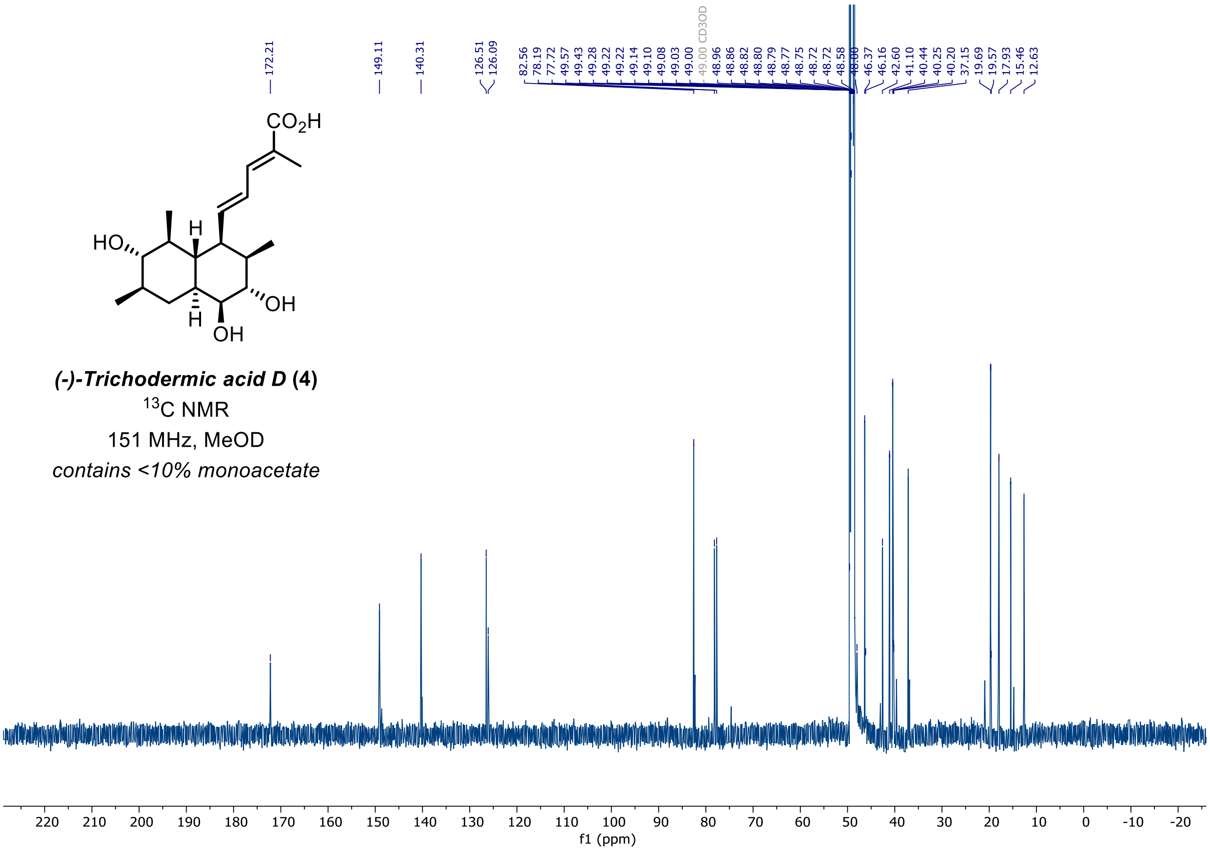

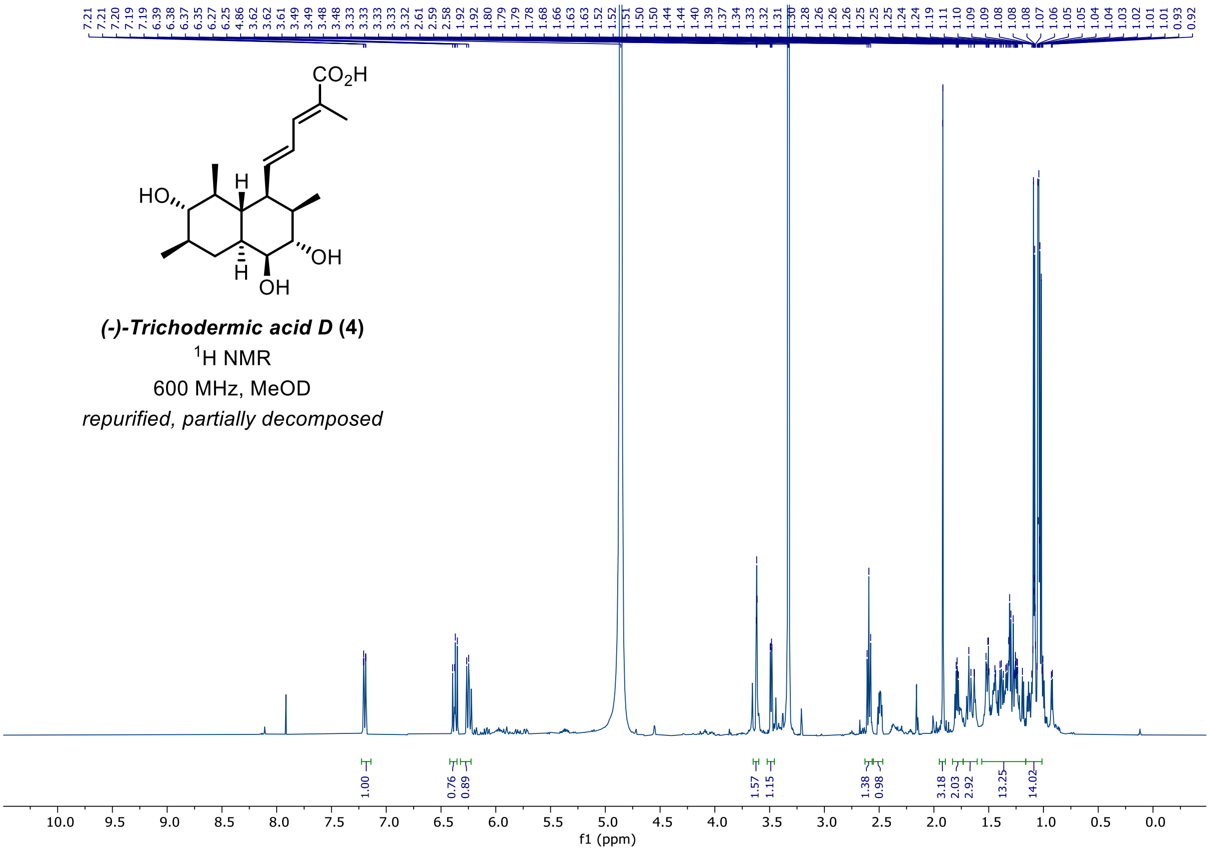
(-)-Trichodermic acid D (4)
1H NMR
600 MHz, MeOD
repurified, partially decomposed
f1 (ppm)

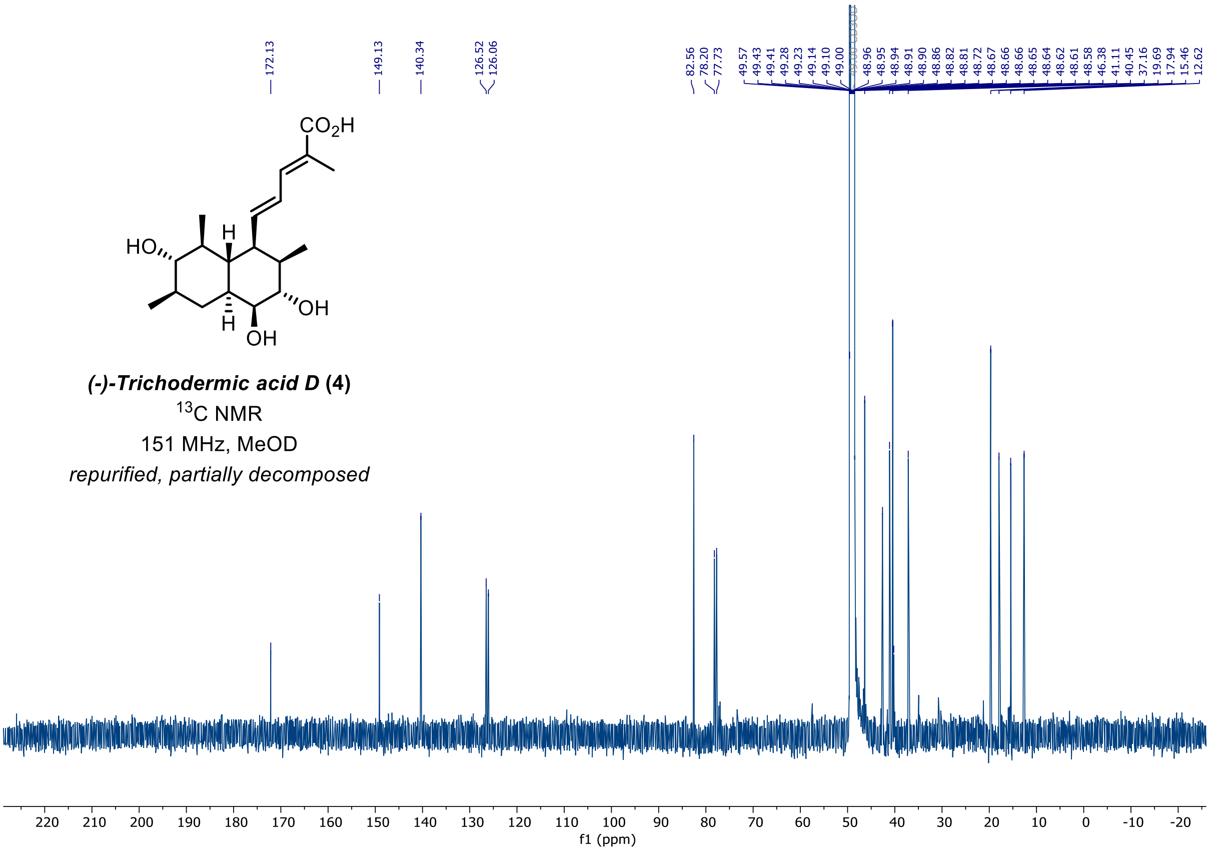
(-)-Trichodermic acid D (4)
13C NMR
151 MHz, MeOD
repurified, partially decomposed
f1 (ppm)

ii NMR spectra of small fragments.

(*R,Z*)-4-Benzyl-3-(but-2-enoyl)oxazolidin-2-one *(R)*-16

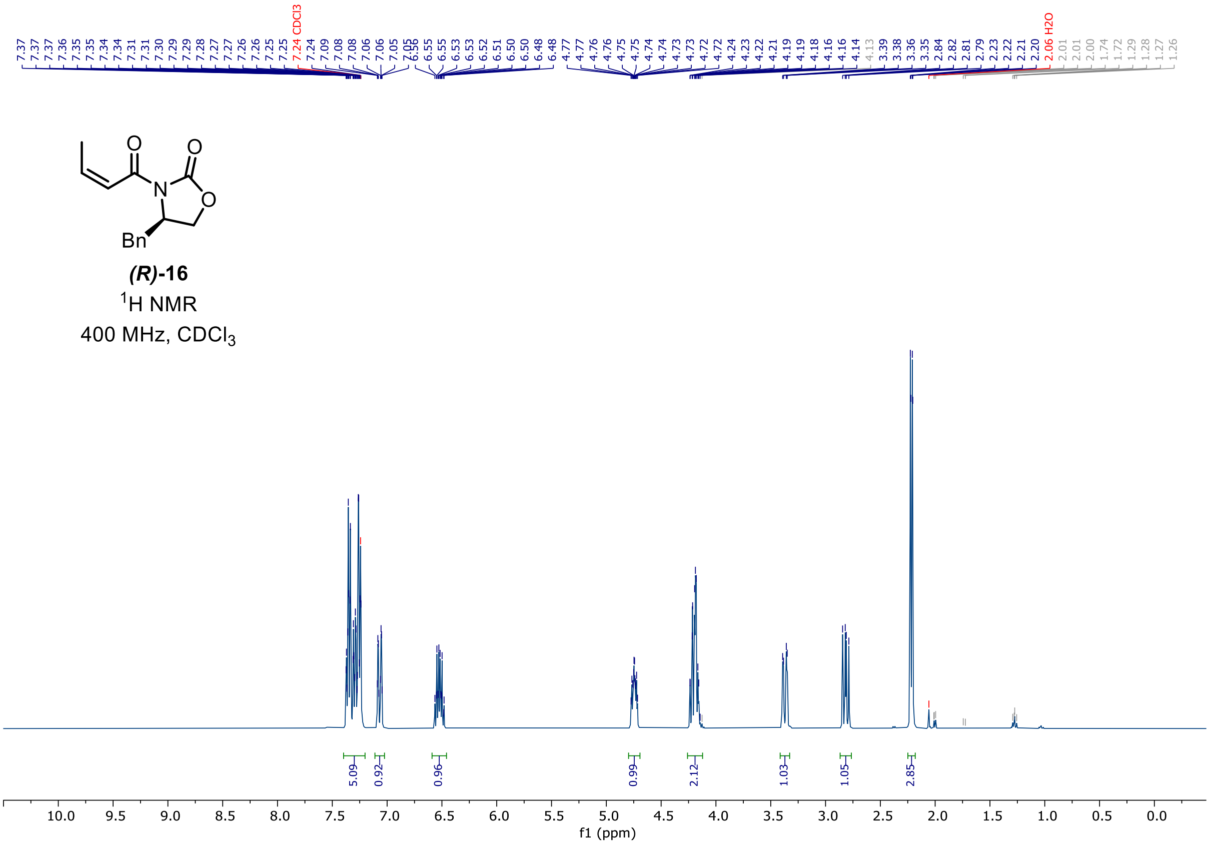

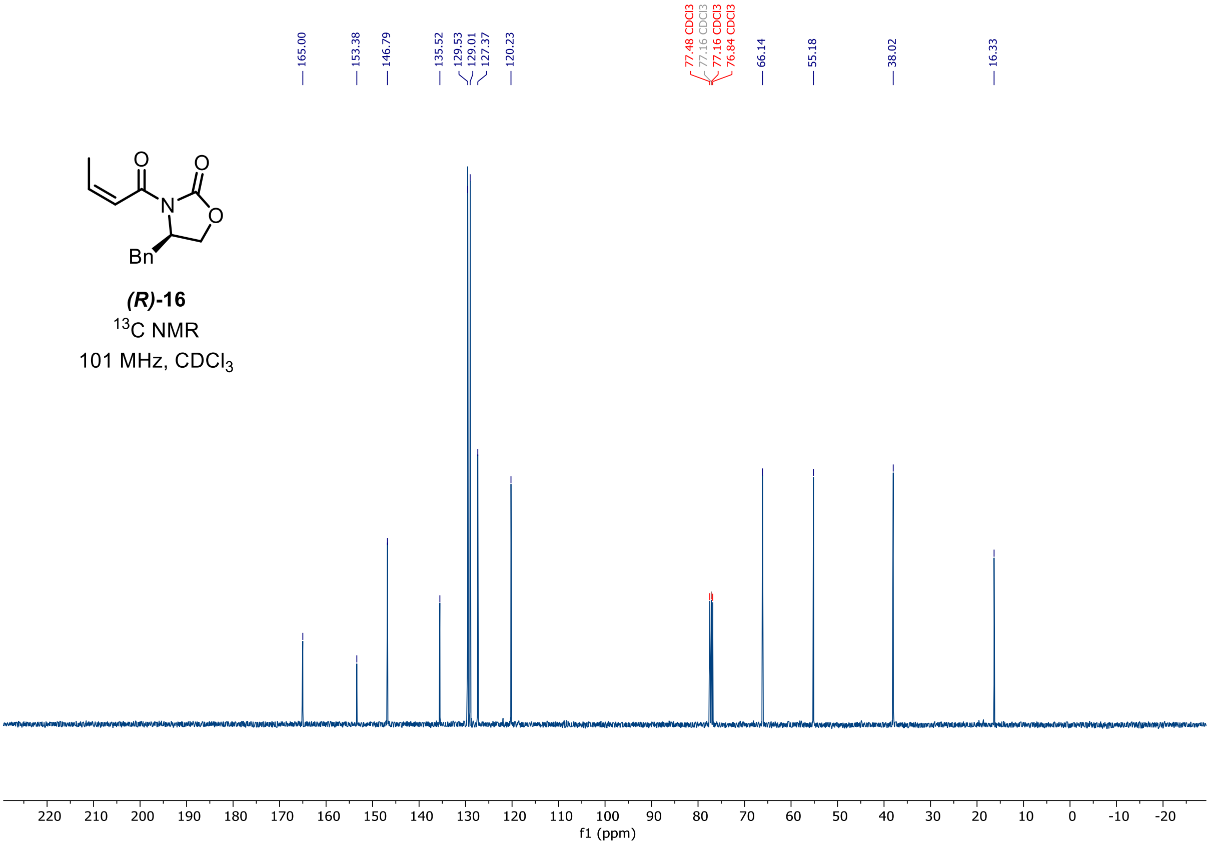

(*R*,*Z*)-4-Benzyl-3-(but-2-enoyl)-5,5-dimethyloxazolidin-2-one *(R)*-S2

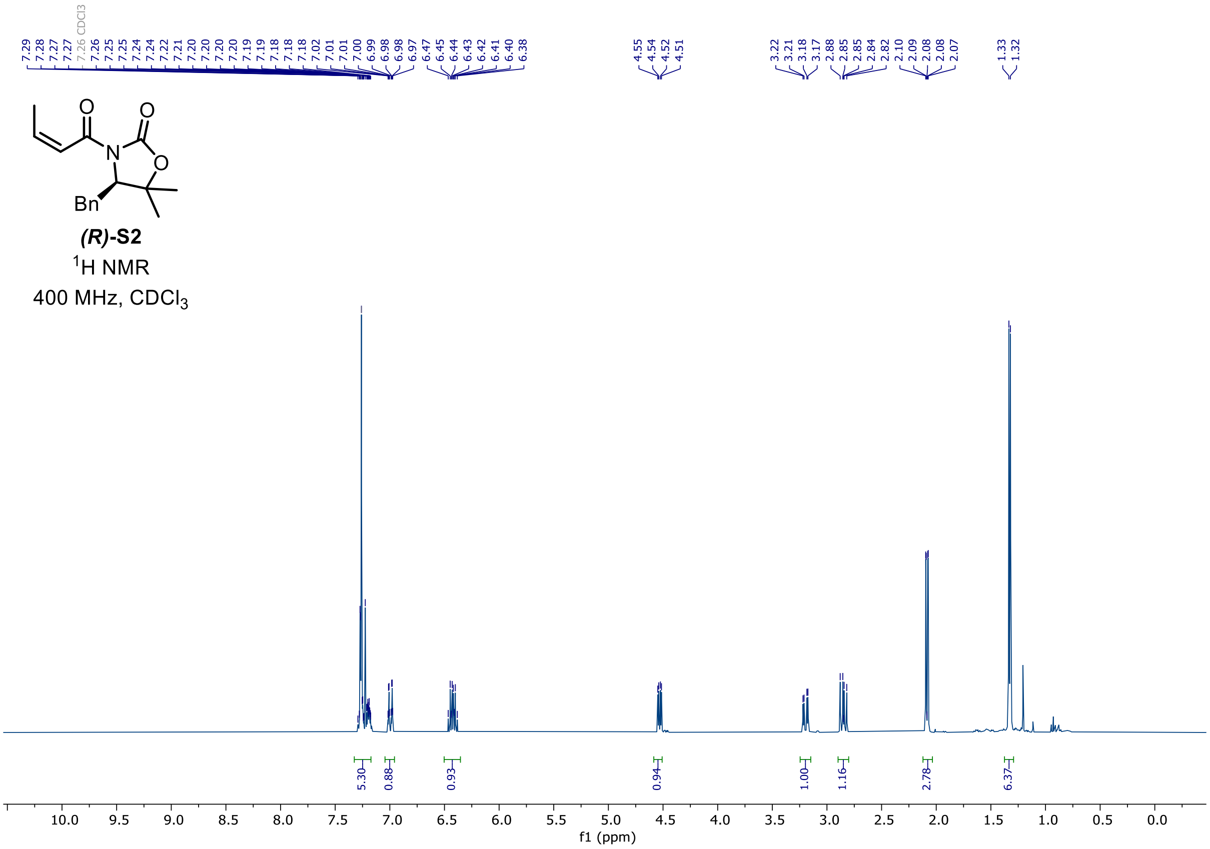

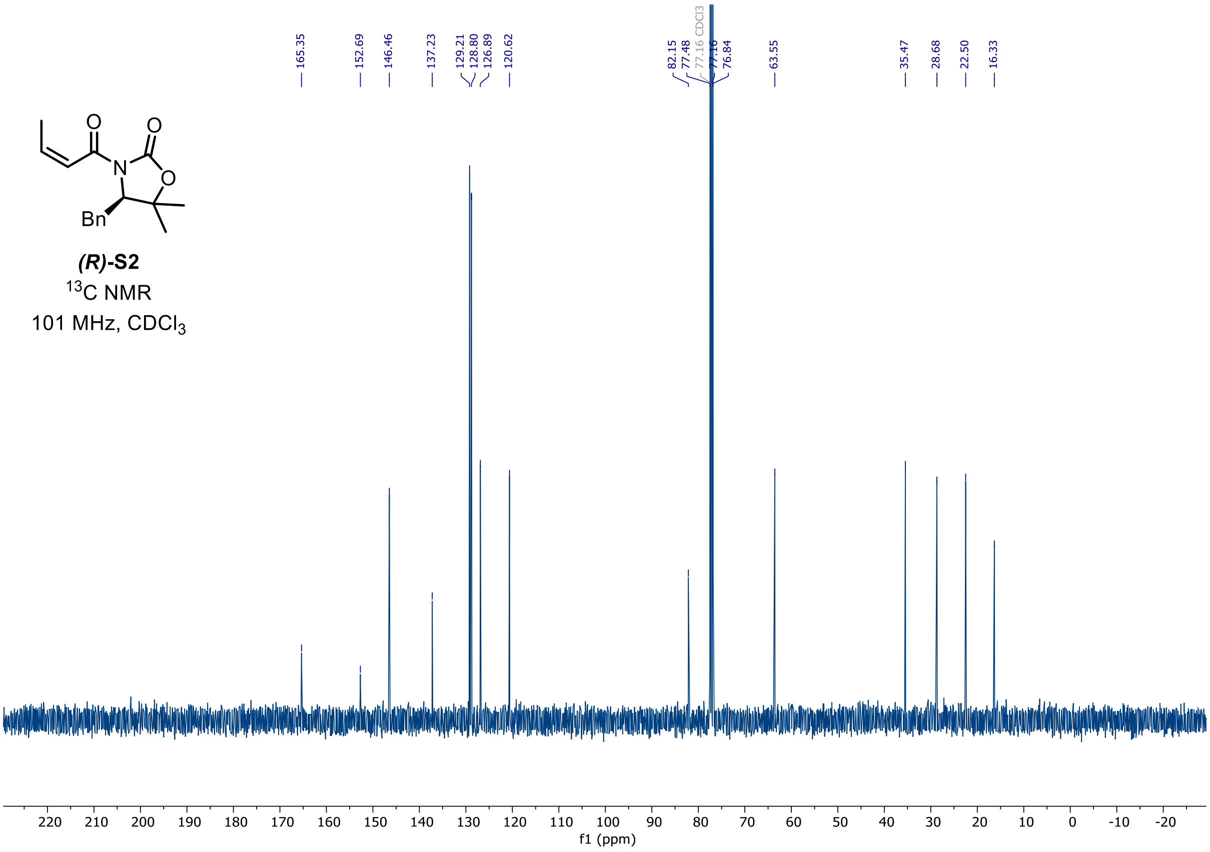

***tert*-Butyl (*E*)-4-(diethoxyphosphoryl)-2-methylbut-2-enoate 22**

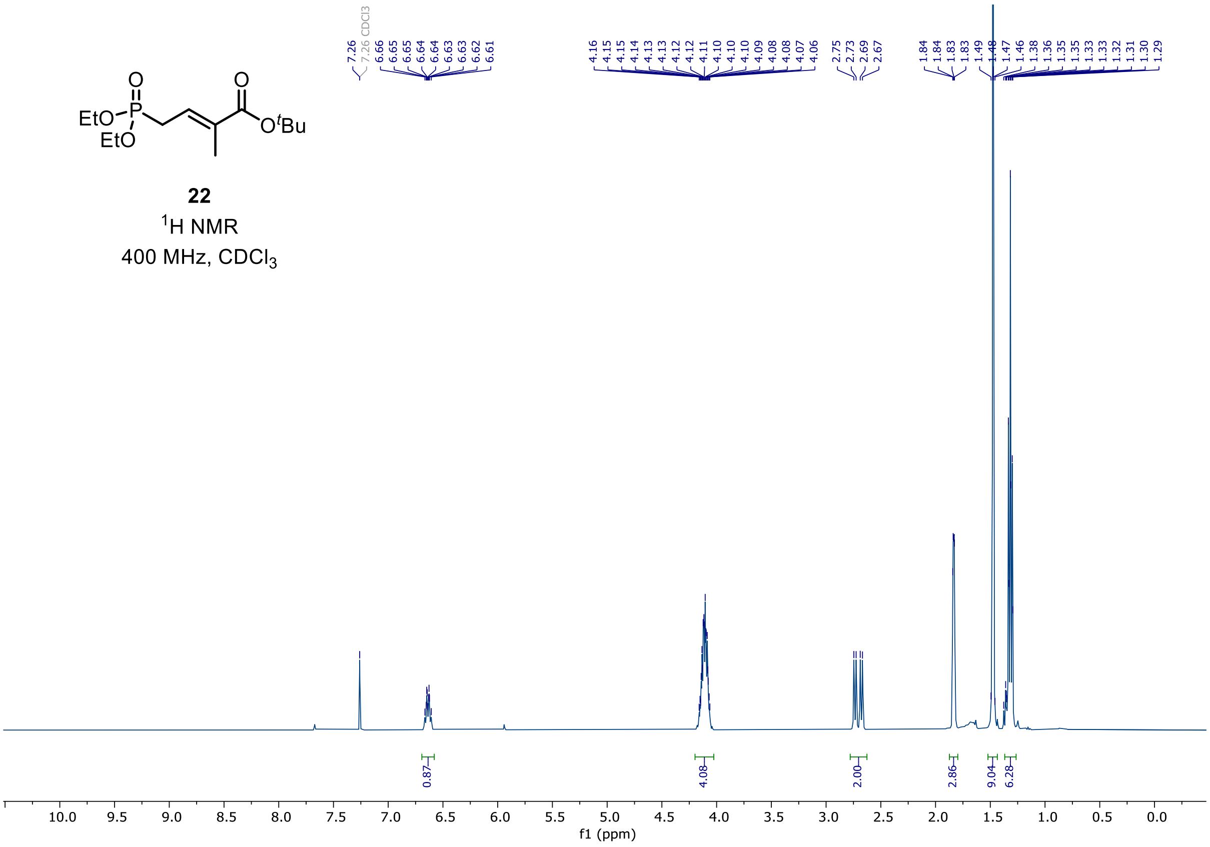

22
^{1}H NMR
400 MHz, $CDCl_3$

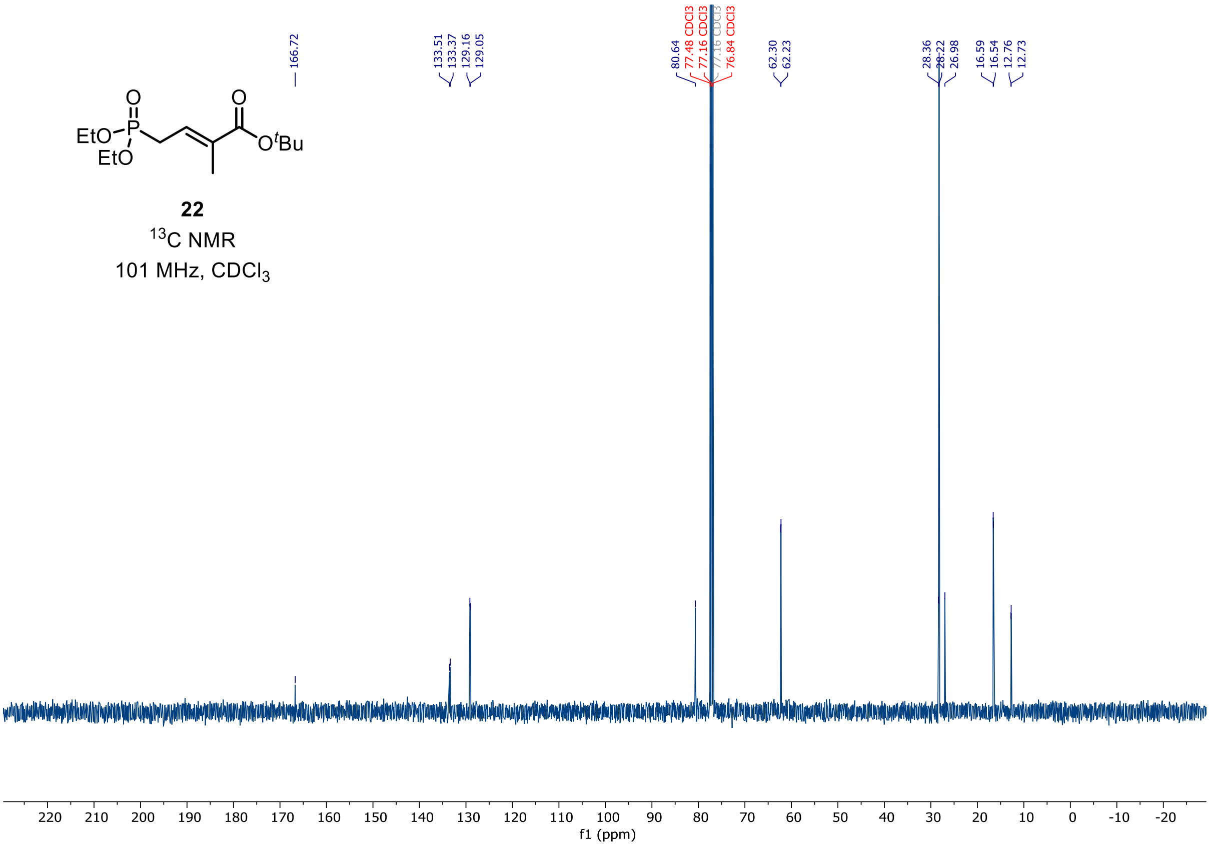

22
^{13}C NMR
101 MHz, $CDCl_3$

iii NMR spectra of side-products and derivatives.

1-((3*S*,4*S*,5*S*)-4-(Dimethyl(phenyl)silyl)-3,5-dimethylcyclohex-1-en-1-yl)ethan-1-one 13

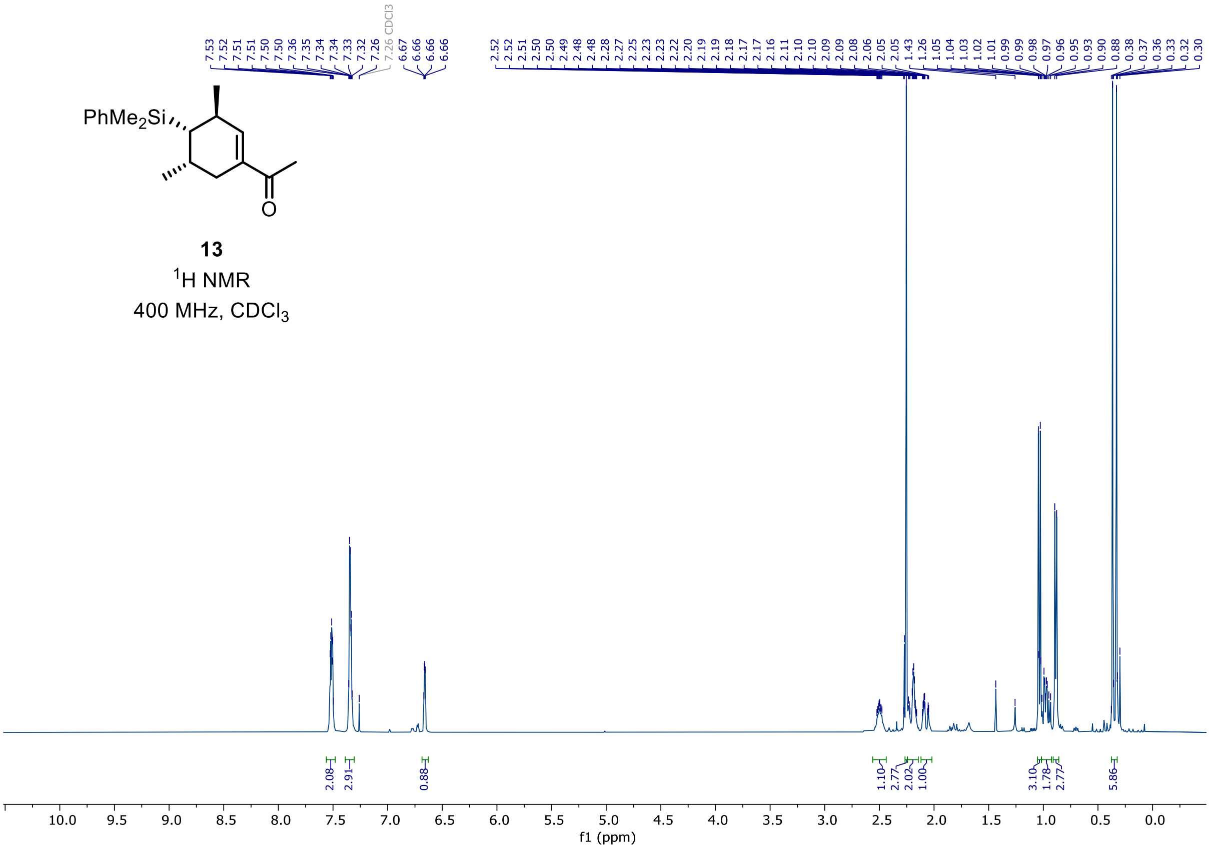

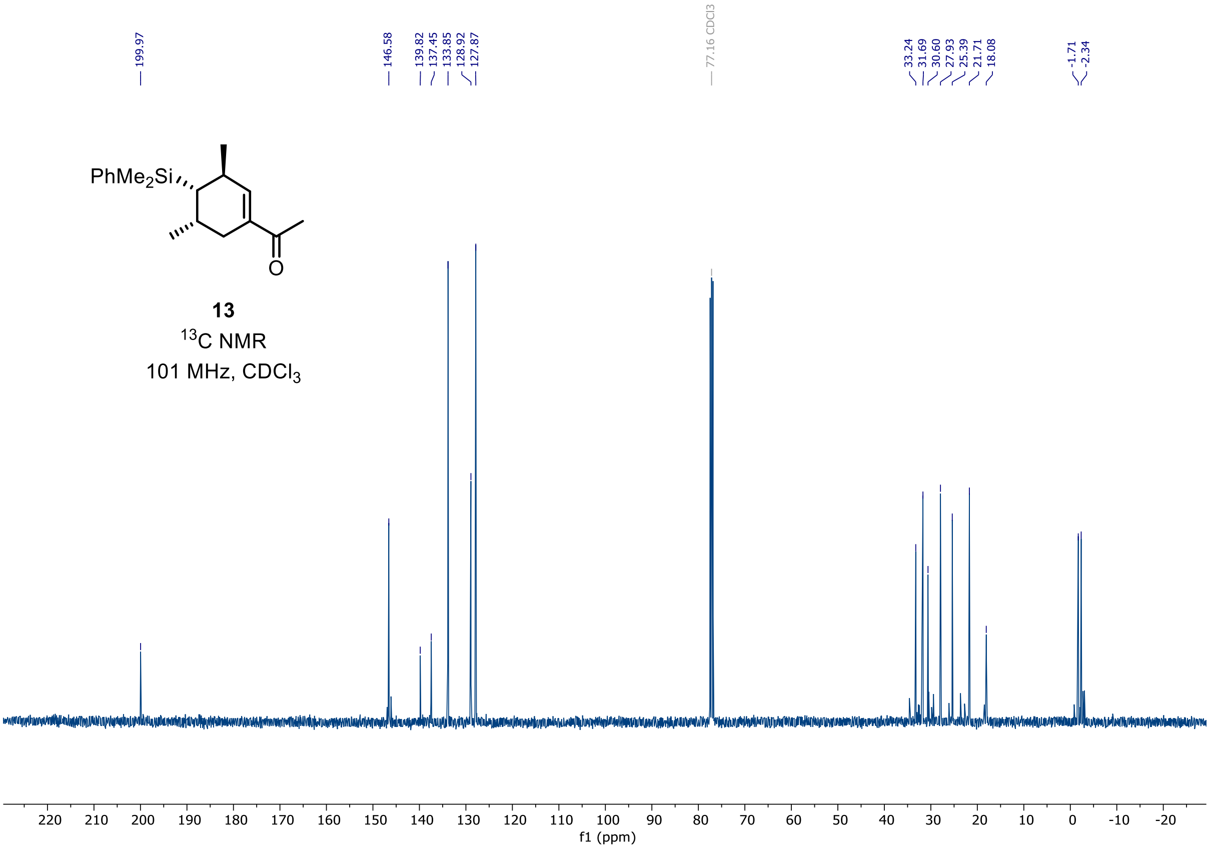

(*E*)-1-(1-((3*S*,4*S*,5*R*)-4-(Dimethyl(phenyl)silyl)-3,5-dimethylcyclohex-1-en-1-yl)ethylidene)-2-(2,4-dinitrophenyl)hydrazine 14

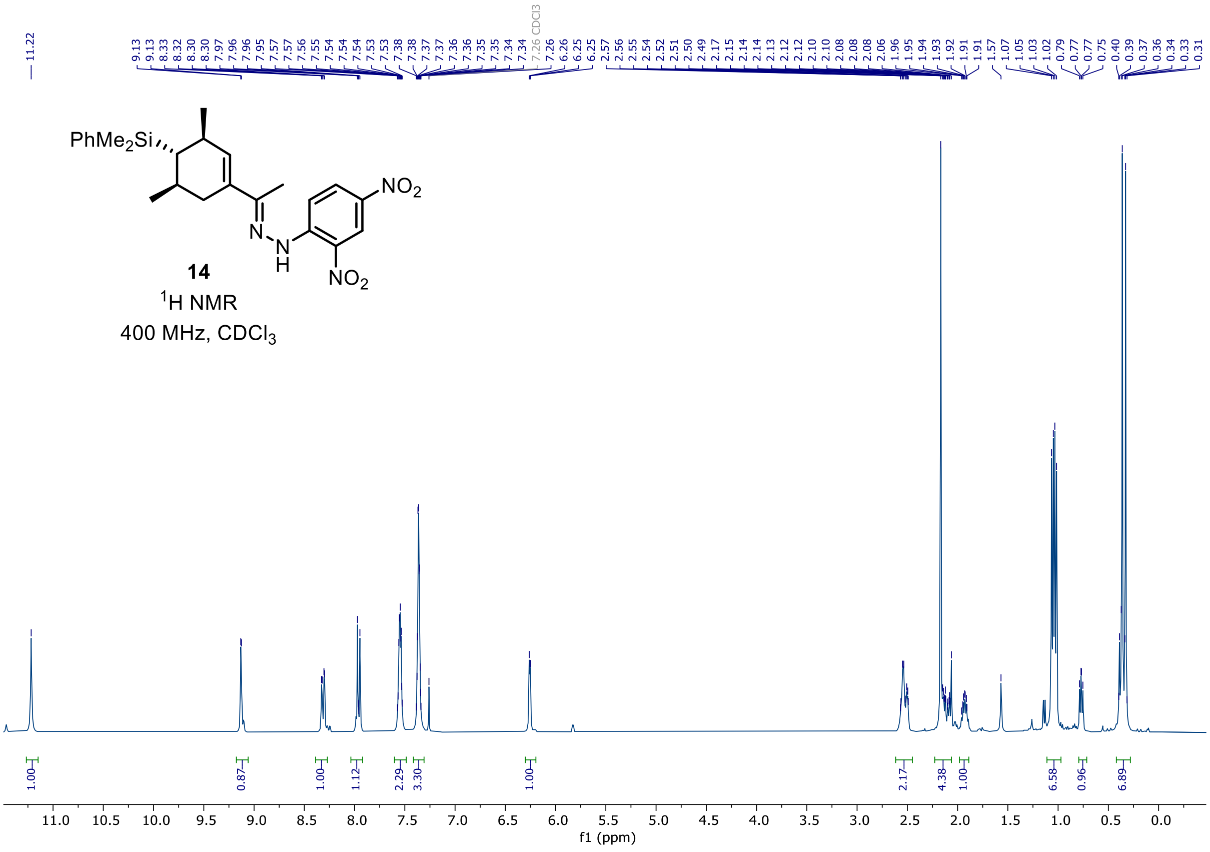

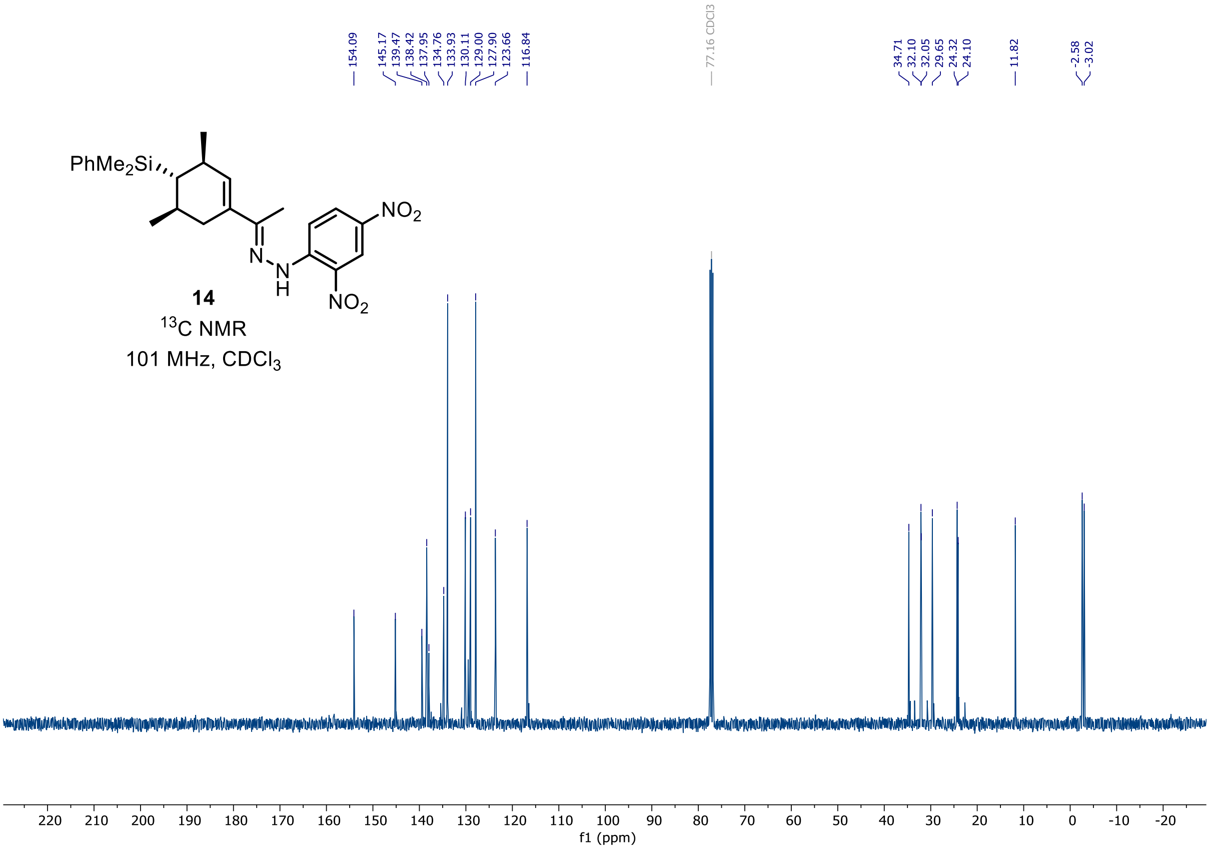

(*S*)-4-Benzyl-3-((1*R*,2*R*,6*R*,7*S*,8*S*,8a*S*)-4-((*tert*-butyldimethylsilyl)oxy)-7-(dimethyl(phenyl)silyl)-2,6,8-trimethyl-1,2,3,5,6,7,8,8a-octahydronaphthalene-1-carbonyl)oxazolidin-2-one *cis-endo*-17

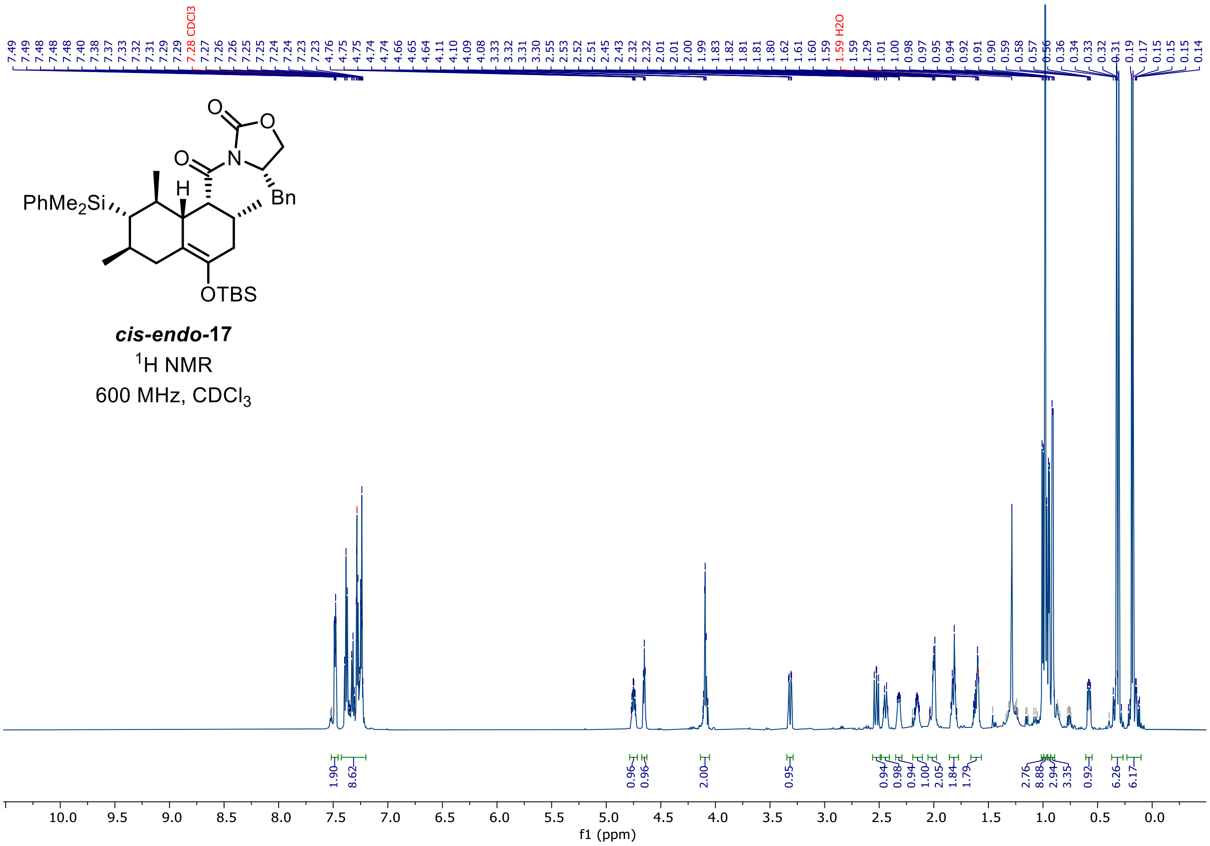

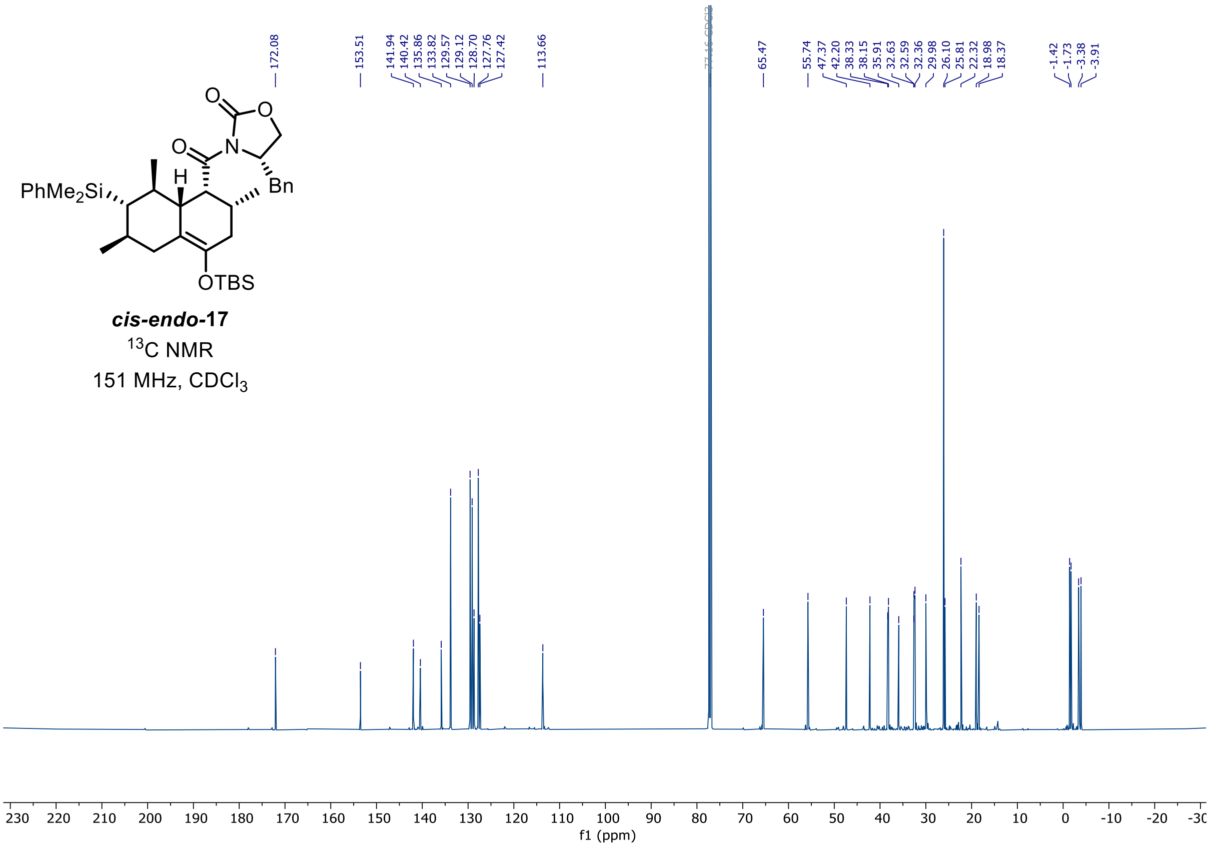

(*S*)-4-Benzyl-3-((1*R*,2*S*,6*R*,7*S*,8*S*,8a*S*)-4-((*tert*-butyldimethylsilyl)oxy)-7-(dimethyl(phenyl)silyl)-2,6,8-trimethyl-1,2,3,5,6,7,8,8a-octahydronaphthalene-1-carbonyl)oxazolidin-2-one *trans-endo*-17

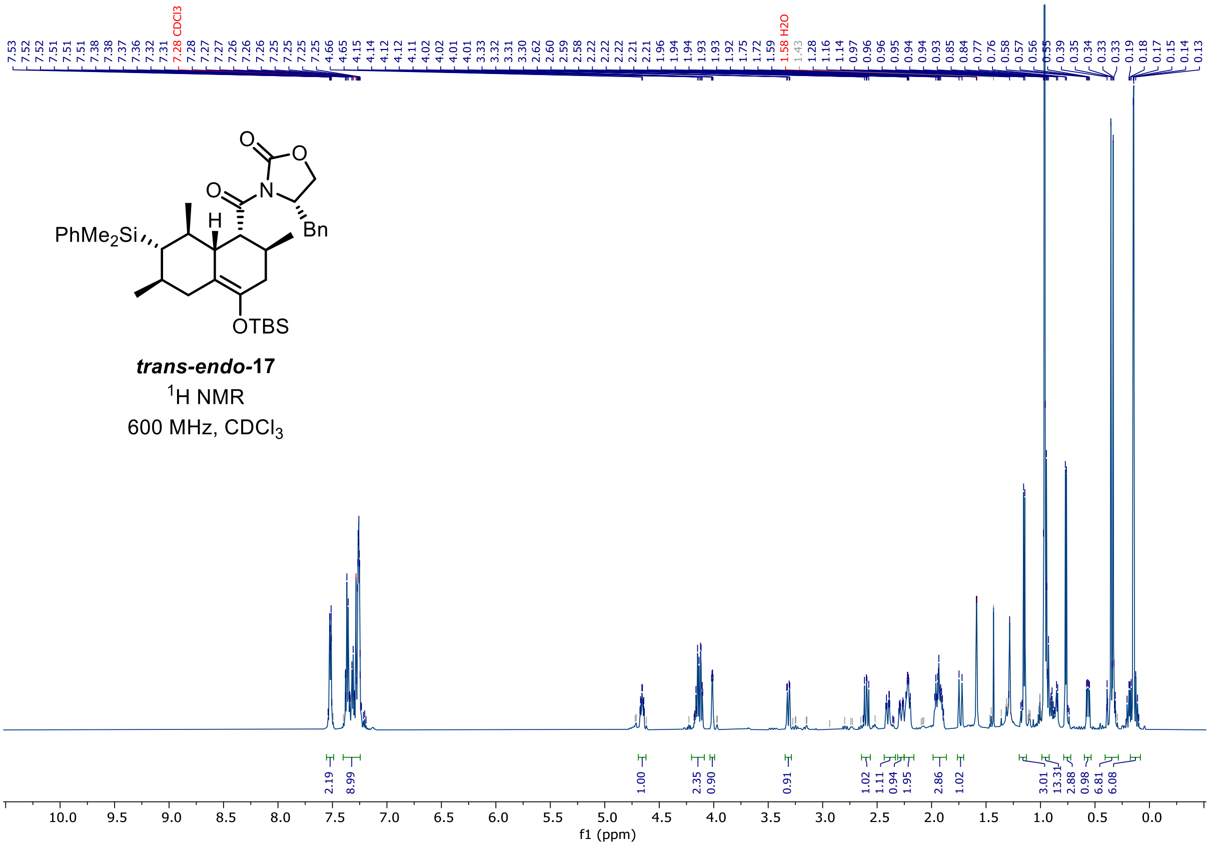

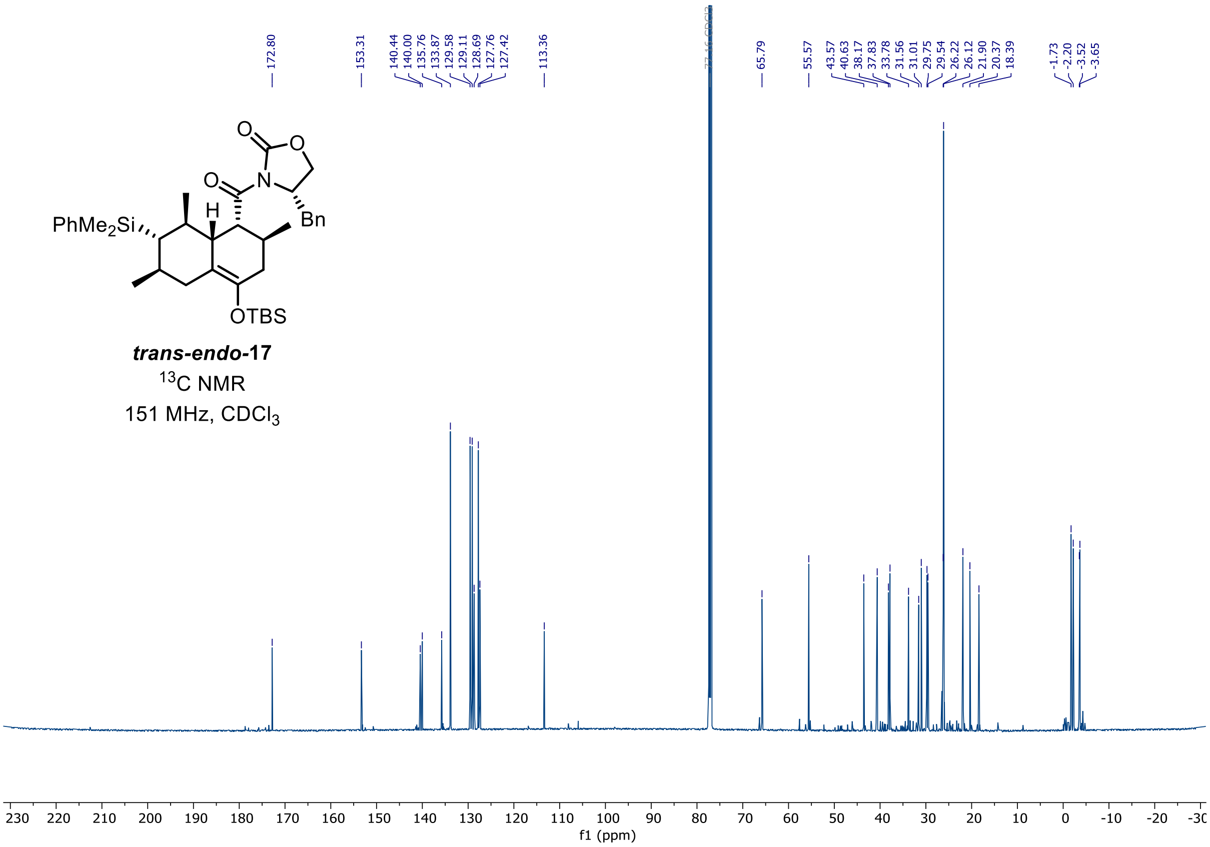

(*R*)-4-Benzyl-3-((1*S*,2*S*,6*R*,7*S*,8*S*,8a*S*)-4-((*tert*-butyldimethylsilyl)oxy)-7-(dimethyl(phenyl)silyl)-2,6,8-trimethyl-1,2,3,5,6,7,8,8a-octahydronaphthalene-1-carbonyl)-5,5-dimethyloxazolidin-2-one *cis-exo*-S6

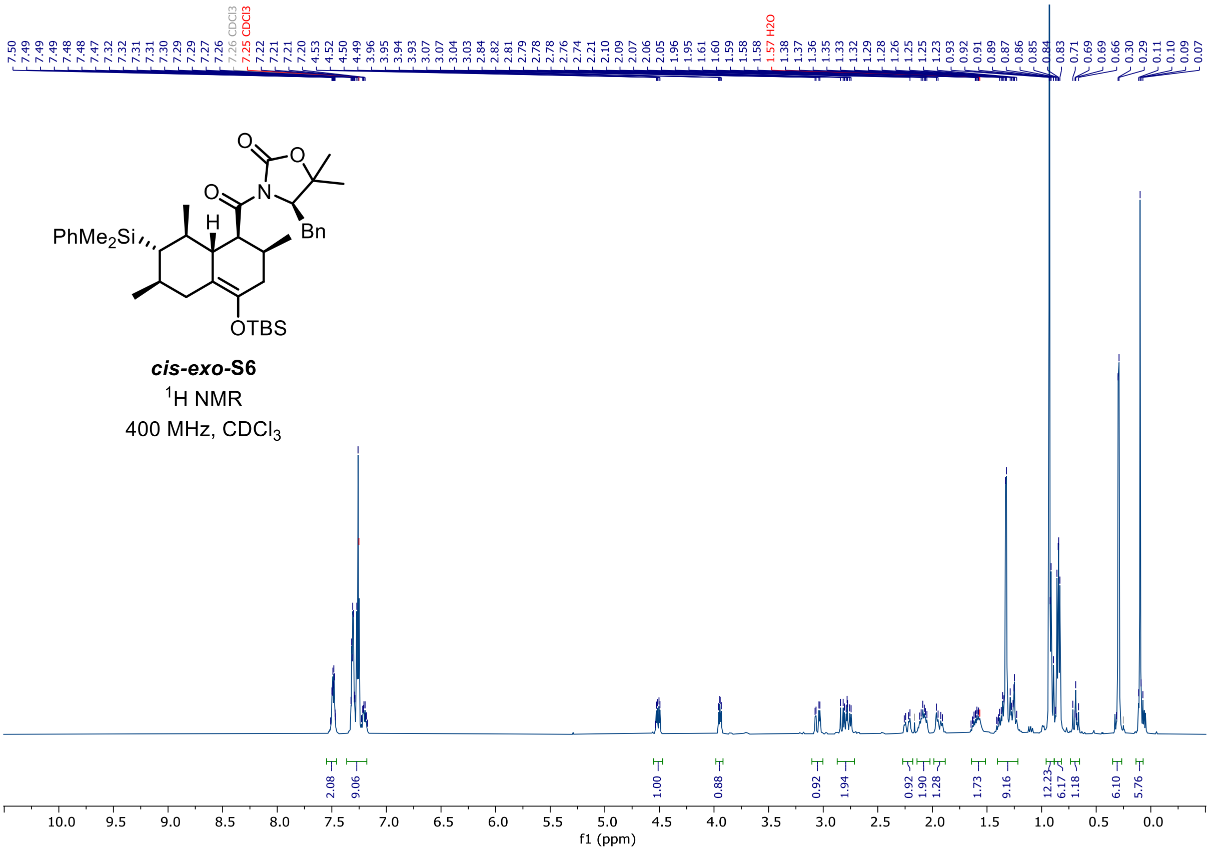

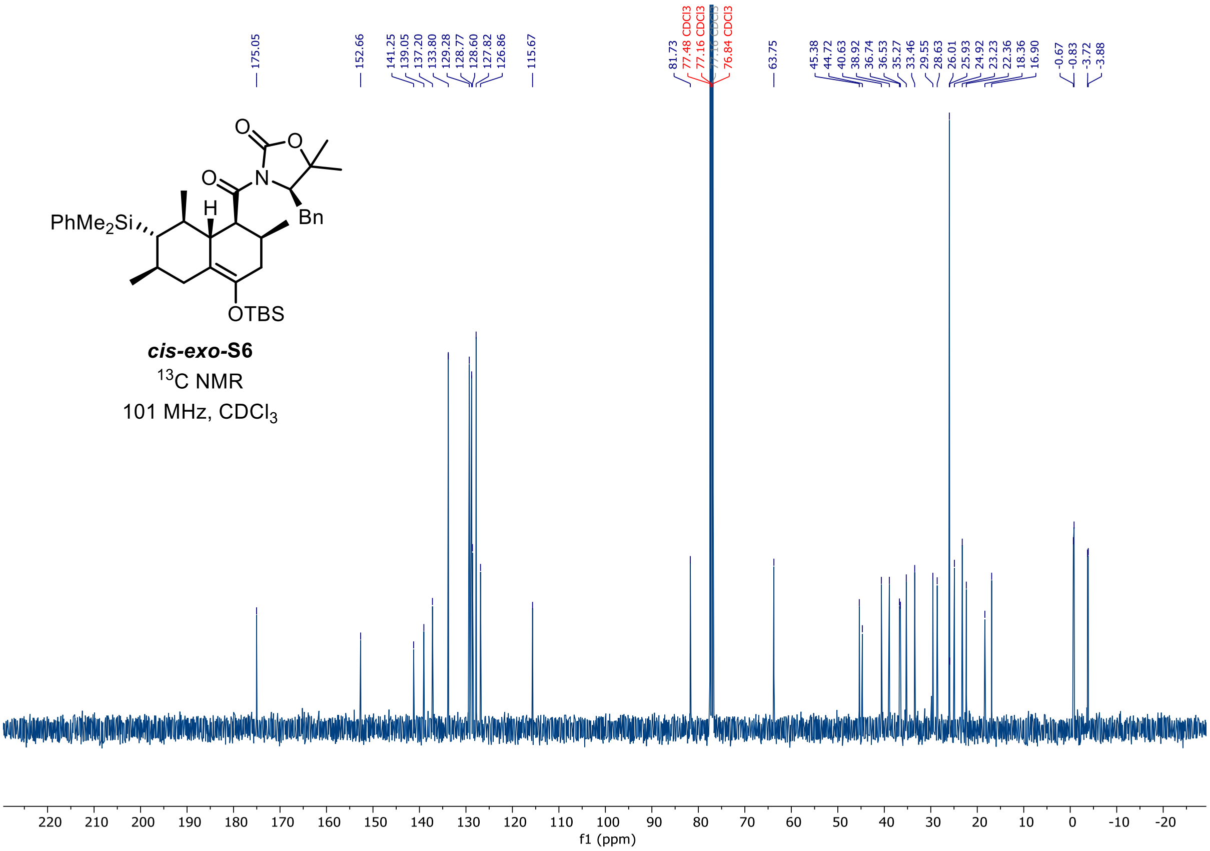

(*R*)-4-Benzyl-3-((1*S*,2*R*,6*R*,7*S*,8*S*,8a*S*)-4-((*tert*-butyldimethylsilyl)oxy)-7-(dimethyl(phenyl)silyl)-2,6,8-trimethyl-1,2,3,5,6,7,8,8a-octahydronaphthalene-1-carbonyl)-5,5-dimethyloxazolidin-2-one *trans-exo*-S6

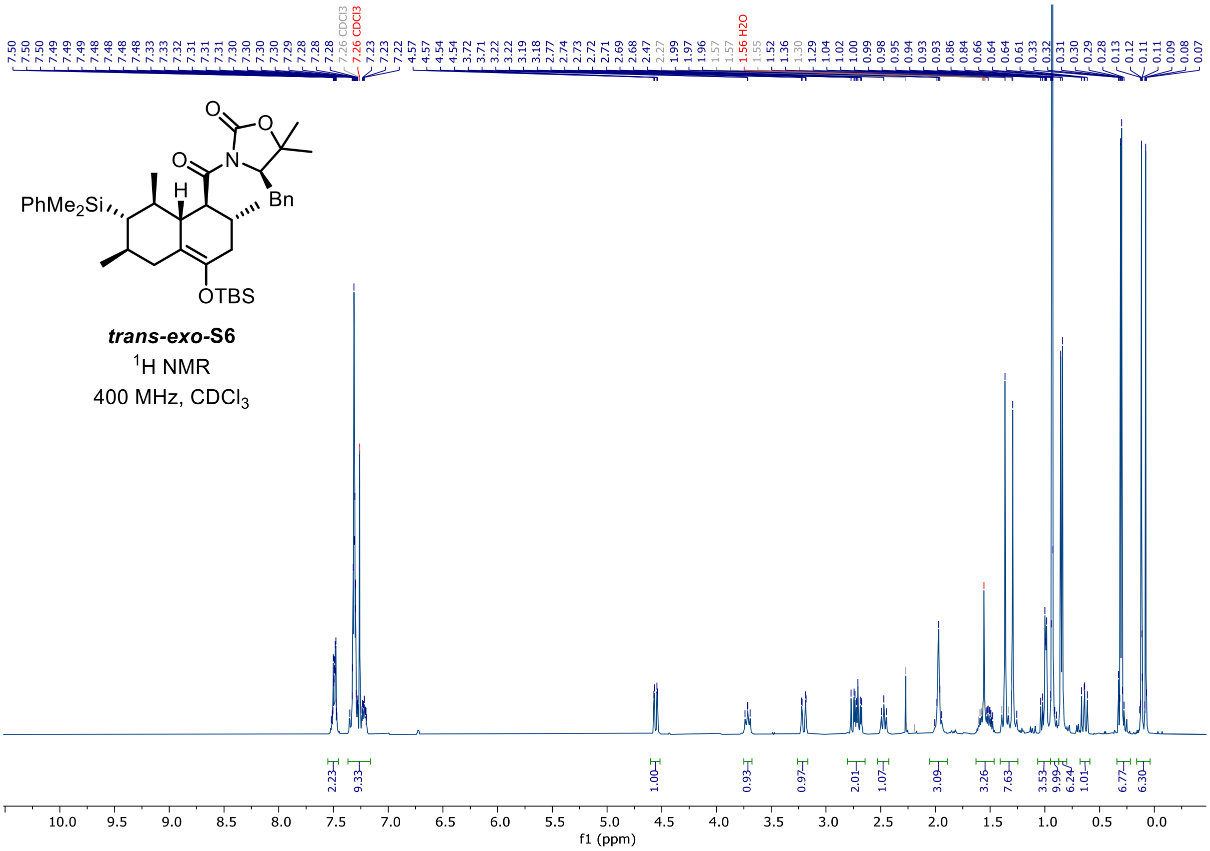

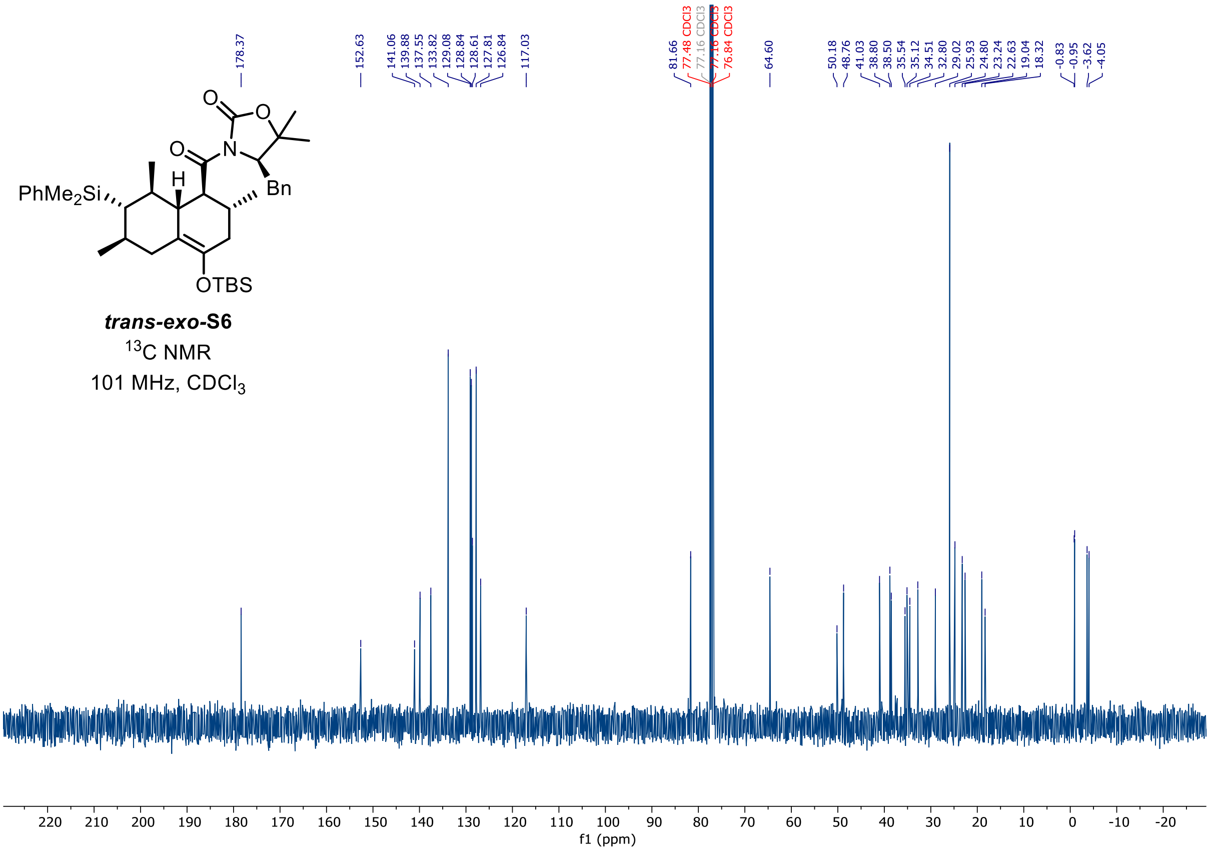

6. X-Ray crystallography Data

(4*R*)-4-Benzyl-3-((1*S*,6*R*,7*S*,8*S*,8a*S*)-4-((tert-butyldimethylsilyl)oxy)-7-(dimethyl(phenyl)silyl)-2,6,8-trimethyl-1,2,3,5,6,7,8,8a-octahydronaphthalene-1-carbonyl)-5,5-dimethyloxazolidin-2-one (*trans-exo*-S6):

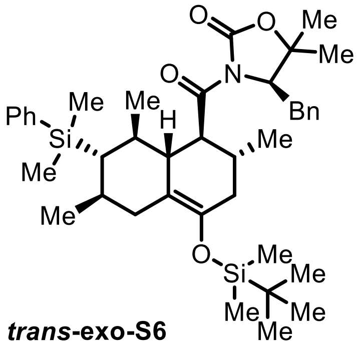

***trans*-exo-S6**

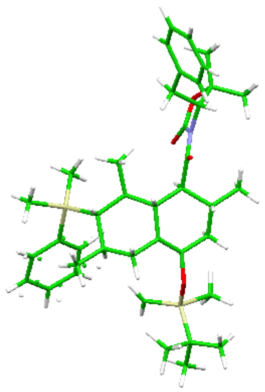

Formula C40 H58 N1 O4 Si2		Recrystallised from pentane by slow evaporation	
Crystal Class	monoclinic	Space Group	P 21
a	10.6497(1)	alpha	90
b	7.2914(1)	beta	101.3318(5)
c	25.9891(1)	gamma	90
Volume	1978.74(3)	Z	2
Radiation type	Cu K\a	Wavelength	1.541840
Dx	1.11	Mr	673.08
Mu	1.108	Temperature (K)	150
Size	0.20x 0.20x 0.80		
Colour	clear pale colourless	Shape	needle
Cell from	35688 Reflections	Theta range	4 to 76
Diffractometer type	multi-scan	Scan type	OMEGA

Absorption type	multi-scan	Transmission range	0.43 0.80
Reflections measured	44082	Independent reflections	8172
Rint	0.0003	Theta max	76.10
Hmin, Hmax	-13 13		
Kmin, Kmax	-9 9		
Lmin, Lmax	-32 32		
Refinement on Fsqd			
R-factor	0.067	Weighted R-factor	0.197
Max shift/su	0.0010		
Delta Rho min	-0.59	Delta Rho max	0.90
Reflections used	8172	sigma(I) limit	-3.00
Number of parameters	480	Goodness of fit	0.959
Flack parameter 0.020(12)			

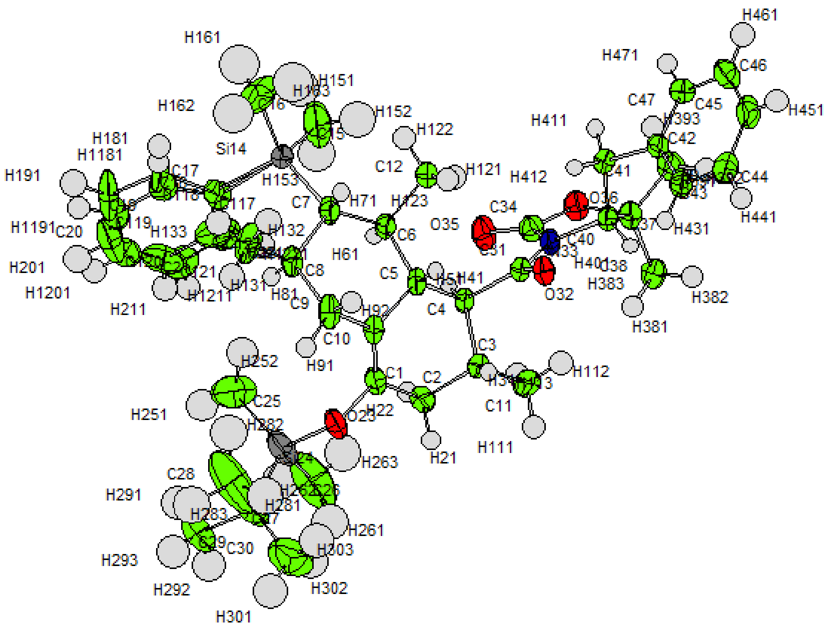

Thermal ellipsoids are drawn at the 50% probability level.

1-((*E*)-1-((3*R*,4*S*,5*S*)-4-(Dimethyl(phenyl)silyl)-3,5-dimethylcyclohexylidene)ethyl)-2-(2,4-dinitrophenyl)hydrazine (14):

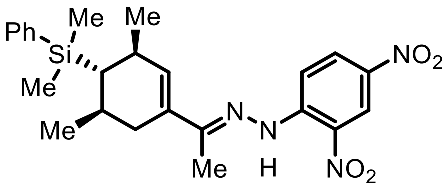

14

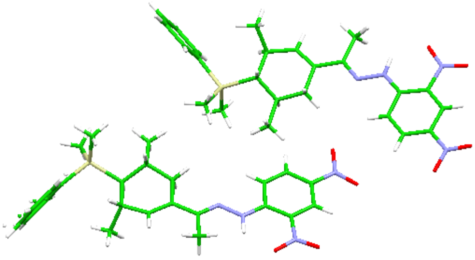

Formula C40 H58 N1 O4 Si2		Recrystallised from $CHCl_3$ by slow evaporation	
Crystal Class	orthorhombic	Space Group	P 21 21 21
a	6.5759(1)	alpha	90
b	15.8853(1)	beta	90
c	47.6568(1)	gamma	90
Volume	4978.24(9)	Z	8
Radiation type	Cu K\a	Wavelength	1.541840
Dx	1.25	Mr	933.22
Mu	1.134	Temperature (K)	100
Size	0.01x 0.10x 0.10		
Colour	translucent pale orange	Shape	plate
Cell from	88128 Reflections	Theta range	2 to 76
Diffractometer type	multi-scan	Scan type	OMEGA
Absorption type	multi-scan	Transmission range	0.64 0.98
Reflections measured	197355	Independent reflections	10295
Rint	0.0007	Theta max	76.28

Hmin, Hmax	-8 7		
Kmin, Kmax	-19 19		
Lmin, Lmax	-59 59		
Refinement on Fsqd			
R-factor	0.089	Weighted R-factor	0.261
Max shift/su	0.0064		
Delta Rho min	-0.40	Delta Rho max	0.74
Reflections used	10293	sigma(I) limit	-3.00
Number of parameters	651	Goodness of fit	0.999
Flack parameter 0.017(59)			

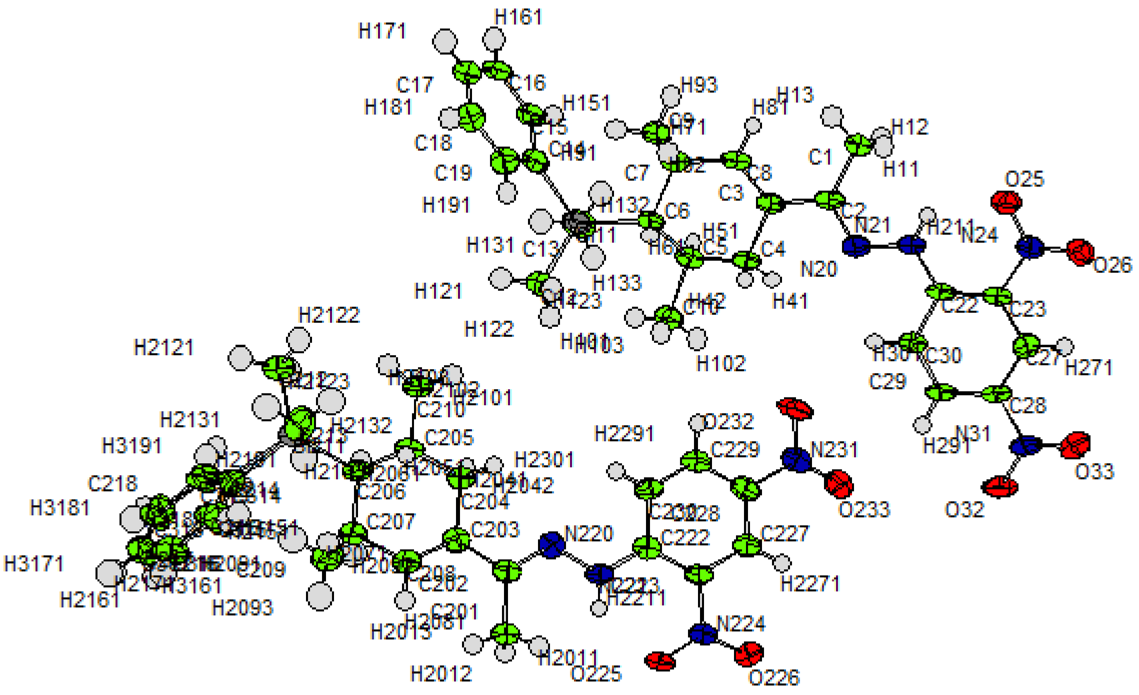

Thermal ellipsoids are drawn at the 50% probability level.

7. References

(1) Cosier, J.; Glazer, A. M. A nitrogen-gas-stream cryostat for general X-ray diffraction studies. *J. Appl. Cryst.* **1986**, *19* (2), 105–107.

(2) Palatinus, L.; Chapuis, G. SUPERFLIP - a computer program for the solution of crystal structures by charge flipping in arbitrary dimensions. *J. Appl. Cryst.* **2007**, *40* (4), 786–790.

(3) Parois, P.; Cooper, R. I.; Thompson, A. L. Crystal structures of increasingly large molecules: meeting the challenges with CRYSTALS software. *Chem. Cent. J.* **2015**, *9* (1), 30.

(4) Cooper, R. I.; Thompson, A. L.; Watkin, D. J. CRYSTALS enhancements: dealing with hydrogen atoms in refinement. *J. Appl. Cryst.* **2010**, *43* (5 Part 1), 1100–1107.

(5) Burke, J. P.; Sabat, M.; Myers, W. H.; Chruma, J. J. Unexpected exo selectivity for an intramolecular Diels–Alder reaction involving a doubly-activated δ-pentenolide dienophile. *Tetrahedron: Asymmetry* **2011**, *22* (1), 31–35.

(6) Shiina, I.; Umezaki, Y.; Murata, T.; Suzuki, K.; Tonoi, T. Asymmetric Total Synthesis of (+)-Coprophilin. *Synthesis* **2018**, *50* (06), 1301–1306.

(7) Ondeyka, J. G.; Giacobbe, R. A.; Bills, G. F.; Cuadrillero, C.; Schmatz, D.; Goetz, M. A.; Zink, D. L.; Singh, S. B. Coprophilin: An anticoccidial agent produced by a dung inhabiting fungus. *Bioorg. Med. Chem. Lett.* **1998**, *8* (24), 3439–3442.

(8) Shiina, I.; Umezaki, Y.; Ohashi, Y.; Yamazaki, Y.; Dan, S.; Yamori, T. Total Synthesis of AMF-26, an Antitumor Agent for Inhibition of the Golgi System, Targeting ADP-Ribosylation Factor 1. *J. Med. Chem.* **2013**, *56* (1), 150–159.

(9) Wu, J.-L.; Yu, Y.-H.; Yao, H.-Z.; Zhao, X.; Yuan, T.; Huang, Y.-H. Trichodermic acids from an endophytic Trichoderma sp. and their antifungal activity against the phytopathogen Botrytis cinerea. *Phytochem. Lett.* **2023**, *56*, 24–29.

(10) Sofian, F. F.; Warahapsari, F. A.; Yoshida, J.; Ito, Y.; Koseki, T.; Shiono, Y. Two new octahydronaphthalene derivatives, trichodermic acids C and D produced by Trichoderma sp. HN-1.1. *Nat. Prod. Res.* **2023**, *37* (3), 484–493.

(11) Liu, Z.; Xu, C.; del Pozo, J.; Torker, S.; Hoveyda, A. H. Ru-Based Catechothiolate Complexes Bearing an Unsaturated NHC Ligand: Effective Cross-Metathesis Catalysts for Synthesis of (Z)-α,β-Unsaturated Esters, Carboxylic Acids, and Primary, Secondary, and Weinreb Amides. *J. Am. Chem. Soc.* **2019**, *141* (17), 7137–7146.

(12) Lemière, G.; Sedehizadeh, S.; Toueg, J.; Fleary-Roberts, N.; Clayden, J. A general synthetic approach to the amnesic shellfish toxins: total synthesis of (−)-isodomoic acid B, (−)-isodomoic acid E and (−)-isodomoic acid F. *Chem. Commun.* **2011**, *47* (13), 3745–3747, 10.1039/C1CC00048A.
